# A Theoretical Study of Positively Curved Circulenes Embedded with Five-Membered Heterocycles: Structures and Inversions

**DOI:** 10.3390/molecules29225335

**Published:** 2024-11-13

**Authors:** Yijian Ma, Tianle Dai, Chengshuo Shen

**Affiliations:** School of Chemistry and Chemical Engineering, Zhejiang Sci-Tech University, Hangzhou 310000, China; 2023211001030@mails.zstu.edu.cn (Y.M.); 2024221002011@mails.zstu.edu.cn (T.D.)

**Keywords:** positive curvature, circulene, polycyclic arene, DFT calculation

## Abstract

Recently, polycyclic arenes with positive curvature have gained increasing significance in the field of material chemistry. This study specifically explores the inversion barriers of a series of positively curved circulenes by using five-membered heterocycles integrated into the backbone of primitive [5]circulenes and [6]circulenes. For hetero[5]circulenes, where one benzenoid ring is replaced by a heterocycle, the inversion barriers exhibit a strong correlation with the rotary angles of the heterocycles, and larger rotary angles result in lower inversion barriers. Additionally, the aromaticity of the circulene undergoes a significant reduction during the inversion process. As the number n of replaced rings increases, the inversion barriers can be adjusted, demonstrating an almost linear relationship with n. In the case of hetero[6]circulenes, molecules bearing heterocycles with small rotary angles also show positive curvatures. Furthermore, we examine the relationship between the radii of the fitted sphere for the circulenes and the inversion barriers, revealing an intriguing inverse proportionality between the fourth power of the radius and the inversion barrier. We anticipate that this research will offer a fresh perspective on studies related to positively curved polycyclic arenes.

## 1. Introduction

Polycyclic arenes with extended *π*-systems have gained increasing prominence in recent material science research, and they have found widespread applications in photoelectronic materials, imaging, and single-molecule devices [1,2,3,4,5,6,7]. For a long period, the primary research focus on polycyclic arenes has been on planar molecules, ranging from smaller compounds like pyrene and perylene to larger ones such as coronene and hexa-peri-hexabenzocoronene (HBC). This had even included expansive nanographenes [8,9,10,11,12]. However, owing to the strong *π*–*π* interactions, planar polycyclic arenes with larger *π*-surfaces always suffer severe solubility issues, which impedes further exploration and practical application [13]. To address this, non-planar elements are strategically integrated into the *π*-surfaces that are composed of sp^2^-hybridized carbon atoms [14,15,16]. One effective approach to this is the introduction of helical elements, giving rise to helicenes and a variety of helicene-derived polycyclic arenes [17,18,19]. These molecules exhibit improved solubility and remarkable chiroptical properties owing to the non-planar nature of the helicene units [20,21,22]. Another innovative method used to render the *π*-surface non-planar is the introduction of positive or negative Gaussian curvatures [23,24]. This can be achieved by incorporating non-benzenoid rings, such as five-, seven- or eight-membered rings, into the *π*-surface [25,26,27,28]. Notable examples include [5]circulene (or corannulene) with positive curvature [7], or [8]circulene with negative curvatures [29,30,31]. These molecules not only exhibit unique geometries, but also display distinct physical and chemical properties owing to their non-benzenoid cycles [32,33].

For these non-planar polycyclic arenes, one of the interesting research focuses is their interconversion process from one stable configuration to another. For instance, helicenes display their interconversion process as the enantiomerization process between *P*- and *M*-handed structures, and a series of primitive carbohelicenes and heterohelicenes have been extensively investigated through theoretical calculations [34,35,36]. In the case of negatively curved polycyclic arenes, their interconversion processes manifest as fluctuations in the *π*-surface [37,38,39,40]. On the other hand, positively curved structures undergo the interconversion process as the inversion of the bowl-like structure, changing from convex to concave [41,42]. Over recent decades, several positively curved polycyclic arenes have been synthesized and their inversion process has been explored [25,43,44,45]. However, the underlying principles of inversion, particularly the relationship between chemical structures, curvatures, and inversion barriers, remain enigmatic. In this study, we embark on a systematic exploration to unravel the influence of positive curvature on the inversion process for DFT calculations. We examine a range of positively curved polycyclic arenes, where single or multiple benzenoid rings of [5] and [6]circulenes are replaced by five-membered heterocycles. The geometries for both the stable structures and interconversion transition states are studied, and the interconversion barriers are provided. These circulenes show a wide range of the curvatures and exhibit vast differences in terms of the inversion barriers. We expect that this study will bring a deep understanding of the interconversion of non-planar polycyclic arenes, thereby introducing novel concepts for designing positively curved polycyclic arenes or larger nanographenes.

## 2. Results and Discussion

### 2.1. Benchmark Study

**Figure 1:**
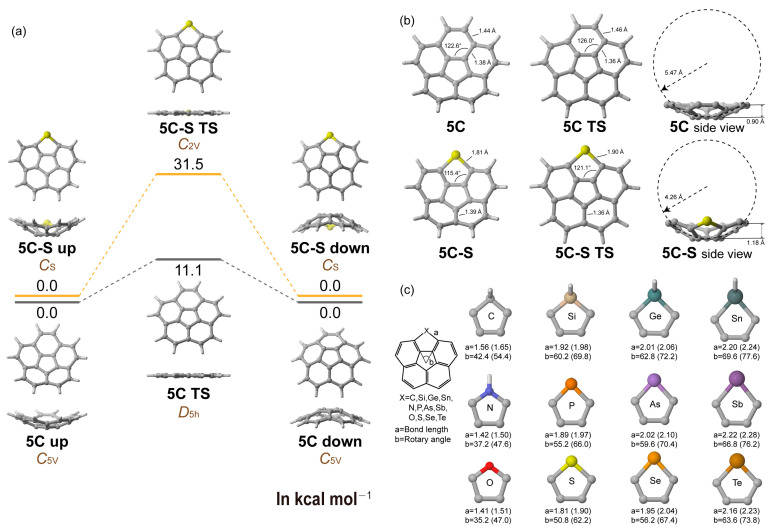
(**a**) Inversion process of **5C** and **5C-S**. (**b**) Geometries of **5C** and **5C-S** in their energy local minima. (**c**) Geometries of the five-membered heterocycle in a series of hetero[5]circulenes (only heterocycles are shown for clarity) in the local energy minimum points. The values given in parenthesis are the values of the transition states.

The inversion of corannulene (or [5]circulene, **5C**) was theoretically studied in 1993 by Schulman et al. [46], and both experimentally and theoretically studied in 2001 by Siegel et al. [47]. Here, we initiated our work by conducting a benchmark study for **5C**, aiming to find appropriate functionals and basis sets for density DFT calculations (Figure 1a). Through a comprehensive benchmark analysis, involving 11 distinct levels of theory in the inversion process, we determined that Grimme’s r^2^SCAN-3c [48], which integrates the r^2^SCAN functional [49], D4 dispersion correction [50,51,52], geometrical counterpoise (gcp) corrections [53], and the mTZVPP basis set [48], is well suited for use in this investigation. Furthermore, several reference studies have also indicated that this method offers a high cost efficiency while maintaining a commendable level of accuracy [54,55]. Details are listed in Figure S1.

### 2.2. Inversion of [5]Circulenes with Single Ring Replaced by Five-Membered Heterocycles

After establishing a suitable calculation method, we proceeded to investigate a series of hetero[5]circulenes (denoted as **5C-X**, where **X** represents the heteroatom of various five-membered heterocycles; e.g., **5C-S** refers to thiophene-replaced [5]circulene), and five-membered heterocycles such as furan (O), thiophene (S), selenophene (Se), tellurophene (Te), pyrrole (N), Phosphole (P), arsole (As), stibole (Sb), cyclopentadiene (C), silole (Si), germole (Ge), and stannole (Sn) are included in this study (Figure 1b). All these molecules exhibit bowl-like geometries similar to those of primitive corannulene **5C** (Figure S1). The geometries of these five-membered cycles, particularly the rotary angle between the two shared C-C bonds with the adjacent cycles (Figure 1c), influence the bowl-like shapes of different depths. Generally, hetero[5]circulenes with smaller rotary angles appear in more curved geometries. For example, in the case of **5C-S**, the rotary angle of the thiophene cycle is found to be 50.8°, which is smaller than that of the primitive **5C** (65.2°). Correspondingly, the bowl depths are 1.18 Å and 0.90 Å for **5C-S** and **5C**, respectively. To better quantify the bowl-like shapes, we fitted a sphere to the bowl-like *π*-surface and measured the radius of the curvature. When the rotary angles have small values, as in the cases of **5C-O**, **5C-N,** and **5C-C**, the curvature radii are smaller (3.59, 3.71, and 3.96 Å compared to the 5.47 Å recorded for **5C**). As the rotary angle of the five-membered heterocycle increases, the curvature radius decreases, and **5C-Sn**, with the largest rotary angle (69.6°, larger than the six-membered benzenoid cycle in **5C**) has the largest radius (5.87 Å) among these molecules. Interestingly, we also observed that the rotary angles in these positively curved polycyclic arenes were larger compared to those of the individual five-membered cycles, with ref. [35] suggesting that there are greater intramolecular stresses within these curved structures.

**Figure 2:**
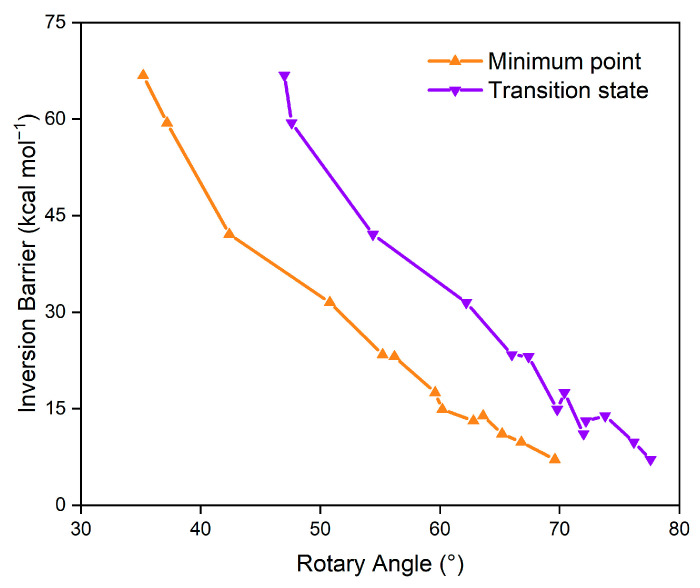
Relationship between the rotary angles (in both local minimum points and transition states) and inversion barriers for **5C-X**.

Subsequently, we conducted an investigation into the inversion of these positive polycyclic arenes (Table 1). For the primitive **5C**, the inversion process pointed to a completely planar geometry in the transition state, with an inversion barrier of 11.1 kcal mol^−1^. Additionally, the rotary angle in this transition state was found to be 72.0°, which was increased by 9.8° compared to the rotary angle at the local energy minimum. In the case of hetero[5]circulene **5C-S**, the transition state exhibits a similar planar structure. However, it possesses a significantly higher inversion barrier of 31.5 kcal mol^−1^. The rotary angle of the thiophene cycle is measured at 62.2°, which is 11.4° larger than the angle at its local energy minimum. This finding suggests that more distortion occurred in **5C-S** compared to **5C**, thus aligning with the higher inversion barrier. When considering [5]circulenes with heterocycles that contain heteroatoms from the second period, where the rotary angles are less than 45°, the inversion barriers are notably elevated. Specifically, for **5C-C**, **5C-N,** and **5C-O**, the inversion barriers are determined to be 41.1, 59.4, and 66.8 kcal mol^−1^, respectively. As the rotary angle increases, the inversion barrier appears to decrease. For instance, **5C-Sn**, which has the largest rotary angle, exhibits an inversion barrier of only 7.1 kcal mol^−1^, which is lower than that of **5C**. Furthermore, a well-correlated negative relationship exists between the rotary angle (in both the local minimum point and the transition state) and the inversion barrier, as illustrated in Figure 2. This finding implies that adjusting the rotary angle of the heterocycle can effectively modulate the inversion barrier of hetero[5]circulenes.

**Figure 3:**
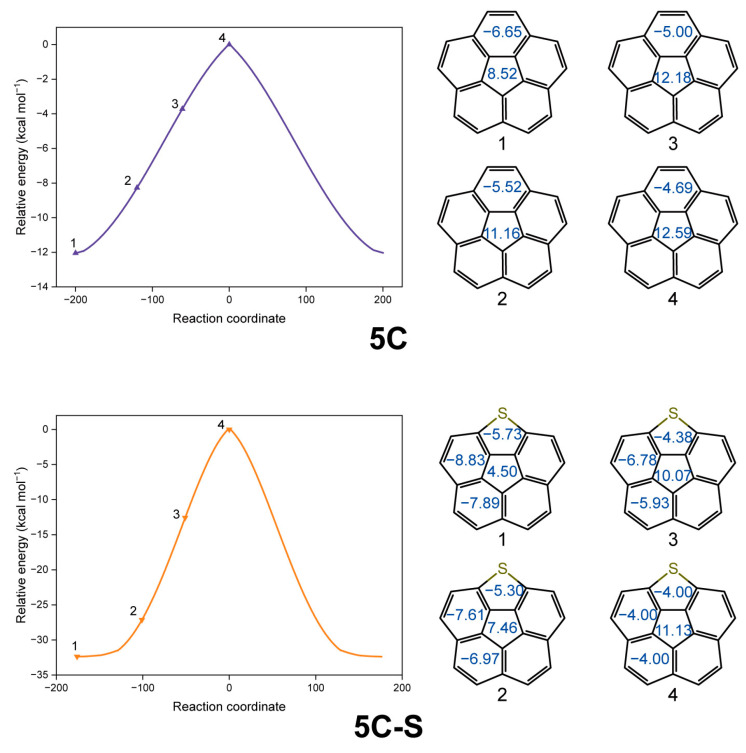
The intermediate structures with the NICS(0) values of **5C** and **5C-S** on the inversion process, according to IRC calculations.

Furthermore, upon investigation using nuclear-independent chemical shift (NICS) analysis, the aromaticity exhibits intriguing variation concurrent with the inversion process (Figure 3). Specifically, at the local energy minimum point, the primitive **5C** demonstrates an NICS(0) value of 8.52 (indicating anti-aromatic character) for the central five-membered ring, whereas the peripheral benzenoid rings exhibit a value of −6.65 (suggesting aromaticity). As the inversion proceeds, the anti-aromaticity of the central ring starts increasing, while the aromaticity of the peripheral benzenoid rings diminishes. Notably, in the transition state, the NICS(0) values shift to 12.59 and −4.69 for the central and the peripheral rings, respectively. Analogously, in the case of **5C-S**, the local minimum point reveals NICS(0) values of 4.50 for the central ring, values of −5.73 for the thiophene ring, and values of −8.83 and −7.89 for the remaining peripheral benzenoid rings. However, in the transition state, the value of the central ring surges to 11.13, while those of both the thiophene ring and the benzenoid ring decline to −4.00. These results imply that the geometric changes during the inversion process impair the aromaticity of the rings within the [5]circulenes.

### 2.3. Inversion of [5]Circulenes with Multiple Rings and Replacement by Five-Membered Heterocycles

**Figure 4:**
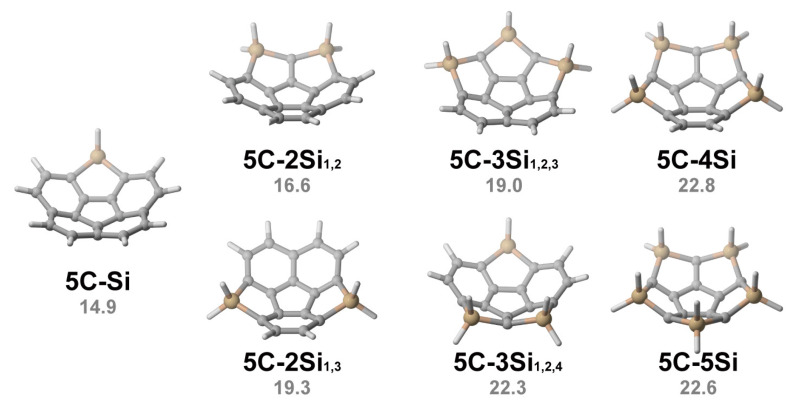
Three-dimensional structures of **5C-*n*Si** and inversion barriers (in kcal mol^−1^).

After investigating the inversion of hetero[5]circulenes via the replacement of a single ring by five-membered heterocycles, we extended our exploration to multiple-ring replacement (e.g., **5C-2X** with two rings replaced by heterocycles, and so forth). The inversion barriers obtained from DFT calculations are summarized in Table 2. Notably, due to the significant intrinsic stresses, compounds **5C-2N_1,2_**, **5C-2O_1,2,_** and **5C-2O_1,3_** underwent decomposition during the examination of the transition states. Here, we take silole-replaced [5]circulenes as examples (Figure 4). With two rings replaced, we identified two isomers, namely **5C-2Si_1,2_** (where the first and the second benzenoid rings are replaced by silole rings) and **5C-2Si_1,3_**. In the case of **5C-2Si_1,2_**, the inversion barrier is calculated to be 16.6 kcal mol^−1^. Comparatively, for **5C-2Si_1,3_**, the barrier increases to 19.3 kcal mol^−1^. This trend is consistent across the other [5]circulenes, where 1,2-isomers exhibit a lower inversion barrier than their 1,3-homologues. It is worth highlighting that, for circulenes incorporating the atoms from the fifth period such as **5C-2Sn_1,2_**, the inversion barriers are notably lower, reaching 3.5 kcal mol^−1^. This reduction can be attributed to the increased rotary angles, which render a more flattened conformation of the bowl-shaped molecules.

**Figure 5:**
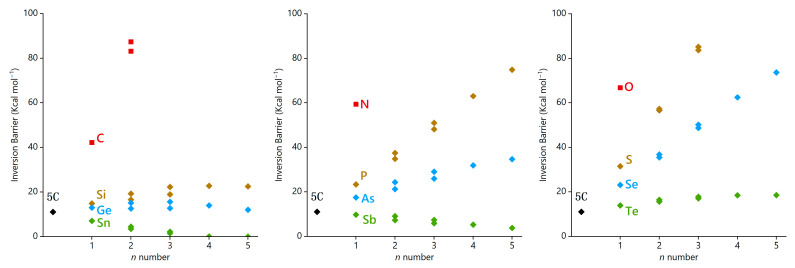
Relationship between the ring number *n* and the inversion barrier for **5C-*n*X**.

When considering hetero[5]circulenes where three rings are replaced by five-membered heterocycles (**5C-3X**), two isomers are observed: one with the first, second, and third rings replaced (**5C-3X_1,2,3_**), and another with the first, second, and fourth rings replaced (**5C-3X_1,2,4_**). It is noteworthy that for all **5C-3X** samples incorporating elements from the second period, we were unable to optimize their transition states. This could be attributed to the small rotary angles, leading to more strained geometries. For **5C-3X** containing the elements from the third and fourth periods, the inversion barriers are noticeably higher compared to those of the corresponding **5C-2X**. This is probably due to the increasing number of heterocycles with smaller rotary angles, which elevates the inversion difficulty. Meanwhile, for **5C-3Sn** and **5C-3Sb** where the heteroatoms from the fifth period, a decrease of the inversion barriers is observed, with **5C-3Sn_1,2,3_** exhibiting a particularly low barrier of merely 1.5 kcal mol^−1^. In the cases of **5C-4X** and **5C-5X**, the hetero[5]circulenes with heteroatoms from the second period as well as **5C-4S** and **5C-5S** have failed to find the transition states owing to the excessive intramolecular stresses. Meanwhile, for **5C-4Sn**, the inversion barrier is reduced to 0.1 kcal mol^−1^, while **5C-5Sn** adopts a fully planar structure. The correlation between the numbers *n* of heterocycles from **5C-*n*X** and the inversion barriers is illustrated in Figure 5. Our findings reveal that for the heterocycles with smaller rotary angles, the inversion barrier increases as the number of heterocycles rises, revealing a nearly linear correlation. Conversely, for the heterocycles with larger rotary angles, it is revealed that the inversion barrier decreases as the number of heterocycles increases.

### 2.4. Inversion of [6]Circulenes with Rings Replaced by Five-Membered Heterocycles

**Figure 6:**
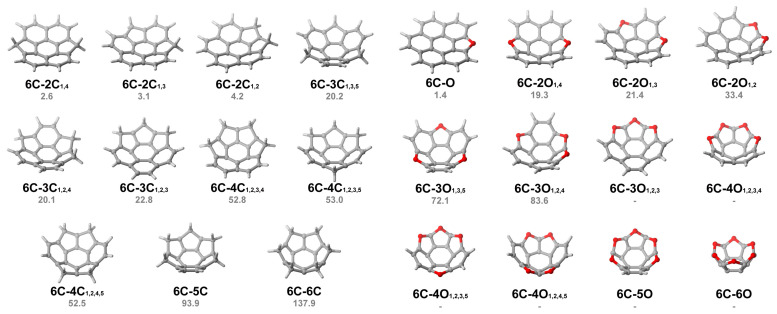

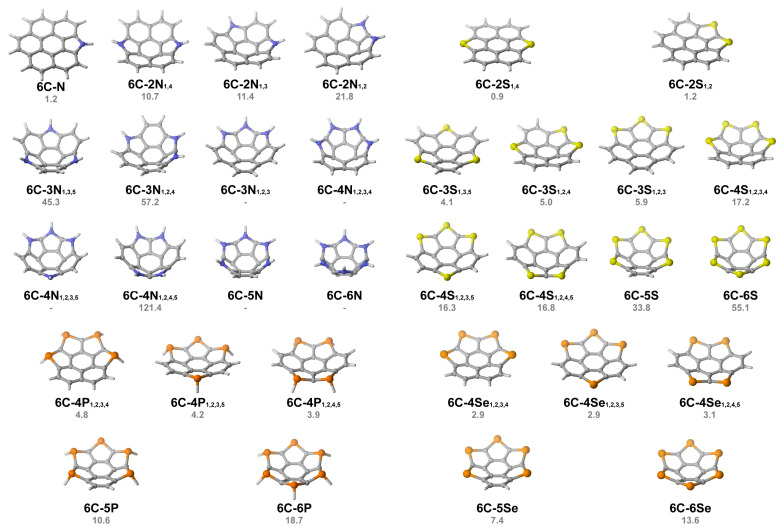
Three-dimensional structures of positively curved hetero[6]circulenes and their inversion barriers (in kcal mol^−1^). Showing circulenes without inversion barriers indicates that their transition states are unable to be optimized.

In the cases of [6]circulenes, despite the fact that primitive [6]circulene (**6C**, or coronene) is revealed to be fully planar, certain hetero[6]circulenes, incorporating five-membered heterocycles, exhibit a positive curvature. Many of these compounds feature five-membered heterocycles with small rotary angles. For example, among one-ring-replaced hetero[6]circulenes, only **6C-N** and **6C-O**, which contain heteroatoms from the second period, display positively curved structures. The fitted spheres for these compounds display radii of 78.62 and 17.91 Å, respectively. Consequently, their inversion barriers are 1.2 and 1.4 kcal mol^−1^, respectively. As the number of heterocycles increases, more hetero[6]circulenes exhibit positive curvatures. In the case of **6C-2X**, three isomers are identified, namely **6C-2X_1,2_**, **6C-2X_1,3,_** and **6C-2X_1,4_**. As the replaced ring’s position shifts from the adjacent (1,2) position to the counterpoint (1,4), the inversion barriers decrease. For example, **6C-2O_1,4_** has an inversion barrier of 19.3 kcal mol^−1^, and the value rises to 21.4 kcal mol^−1^ for **6C-2O_1,3_**, up to the highest value of 33.4 kcal mol^−1^ for **6C-2O_1,2_**. A similar trend is observed for **6C-2C** and **6C-2N**. Additionally, as the number of heterocycles increases, the hetero[6]circulenes with heteroatoms begin to show positive curvatures from the third period. For example, despite the fully planar geometry of **6C-S**, compounds **6C-2S_1,2_** and **6C-2S_1,4_** have fitted spheres with radii of 16.47 and 57.89 Å, respectively, and inversion barriers of 1.2 and 0.9 kcal mol^−1^, respectively. With more heterocycles introduced into the skeleton of the [6]circulenes, the inversion barriers also vary divergently depending on the type of heteroatom. From **6C-3C** to **6C-6C**, the inversion barrier escalates from 20.1 to 137.9 kcal mol^−1^. However, for **6C-4N** to **6C-6N** and **6C-4O** to **6C-6O**, owing to the excessive intramolecular stresses, the transition states are not able to be found from the calculations. Besides, when four selenophenes replace the benzenoid rings in [6]circulenes, the molecules become positively curved, and the inversion barrier reaches 13.6 kcal mol^−1^ in the case of **6C-6Se**. Meanwhile, all the hetero[6]circulenes with heteroatoms from the fifth period remain planar. Figure 6 provides a comprehensive list of the positively curved hetero[6]circulenes and their corresponding inversion barriers.

### 2.5. Discussions on the Relationship Between Curvatures and Inversion Barriers

**Figure 7:**
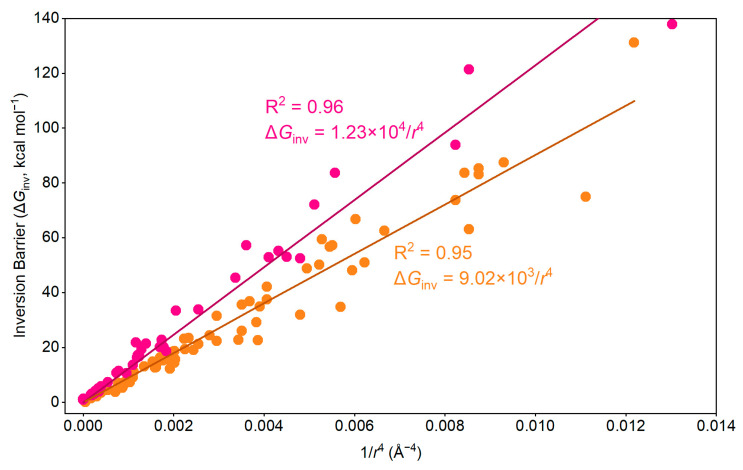
Relationship between the *r* (radii of the sphere fitting for the bowl-like shapes) and the inversion barriers (orange for [5]circulenes and red for [6]circulenes).

Here, in Figure 7, we present the relationship between the curvature radii and the inversion barriers of all the hetero[*n*]circulenes investigated in this study. The curvature radii are calculated using sphere fitting for the bowl-like shapes, the horizontal coordinate is set as the reciprocal of the fourth power of the radii (*r*), and the vertical coordinate is set as the Gibbs free energy of the inversion barriers (Δ*G_inv_*). We found that these two variables show a well-correlated proportional relationship with both positively curved [5]circulenes and [6]circulenes:

Δ*G*_inv_ = *k*/*r*^4^ (1)

This linear correlation indicates that, with the increase in the radius of the curvature of the positively curved [*n*]circulenes, the energy of the inversion increases significantly.

## 3. Methods

Density functional theory (DFT) calculations were performed using the ORCA 5.0.4 program [56,57]. The input files of the ORCA program were prepared with the help of the Multiwfn 3.8-dev program [58,59]. Geometrical optimization calculations were conducted using Grimme’s r^2^SCAN-3c [48] in the gas phase. Harmonic vibration frequency analysis was performed on the optimized geometries to determine the energy local minima with no imaginary frequency and the transition states with only one imaginary frequency. Intrinsic Reaction Coordinate (IRC) analysis was performed for each transition state to ensure the connection between the two energy local minima. Due to the relatively flat potential energy surface of the circulene isomerization process, the step size for the first step of the IRC calculation was specially adjusted to 1.5 mEh (energy difference) to ensure proper progression. The rest SD step size was set to 0.08 Bohr, and the correction step was set as 0.25, which was multiplied by the length of the SD step (0.02 Bohr). For all the calculations in ORCA, the RIJCOSX method with the matching auxiliary basis sets (mTZVPP/J) was applied [60]. The 3D structures were drawn using the CYLview 1.0 program [61]. 

In the benchmark study, we employed and compared the following six functionals: ωB97M-V [62], r^2^SCAN-3c [48], B3LYP-D4 [63], PBE0-D4 [64], M06-2X-D3 [65,66,67], and ωB97X-D3 [68]. These were divided into def2-SVP and def2-TZVPP [69], giving eleven different levels of theory for use in benchmark calculations. Details are listed in Table S1.

Nucleus-independent chemical shift (NICS) [70] values were calculated at the GIAO-B3LYP/def2-TZVP level for the optimized structures (optimized at r^2^SCAN-3c, in ORCA) using the Gaussian 09 E.01 program [71]. The coordinates of the centroid of the corresponding six benzenoid carbon atoms were used for NICS(0). 

## 4. Conclusions

In conclusion, we focused on analyzing the inversion process of a series of positively curved circulenes, incorporating five-membered heterocycles, through DFT calculations. We extensively investigated [5]circulenes and [6]circulenes with one or multiple benzenoid rings replaced by a rich array of five-membered heterocycles. The significance of the heterocycle type, the number, and the position was emphasized. In particular, the rotary angle of the heterocycles largely affected the shapes of the circulenes as well as the inversion barriers. With the increasing rotary angles, the circulenes tended to adopt more flattened structures, thus resulting in lower inversion barriers. Furthermore, we summarized the correlation between the radius of the curvature, determined through sphere fitting, and the inversion barrier. This correlation revealed an inversely proportional relationship between the fourth power of the radius and the inversion barrier. We anticipate that this systematic investigation into the geometrical inversion of positive curvature will provide a valuable research framework for the future exploration of curved polycyclic arenes. 

## Figures and Tables

**Table 1:** Rotary angles (in both local minimum points and transition states) and inversion barriers of **5C-X**.

Label	Rotary Angle	Rotary Angle (TS)	Inversion Barrier ^1^
**5C**	65.2°	72.0°	11.1
**5C-C**	42.4°	54.4°	42.1
**5C-Si**	60.2°	69.8°	14.9
**5C-Ge**	62.8°	72.2°	13.1
**5C-Sn**	69.6°	77.6°	7.1
**5C-N**	37.2°	47.6°	59.4
**5C-P**	55.2°	66.0°	23.4
**5C-As**	59.6°	70.4°	17.5
**5C-Sb**	66.8°	76.2°	9.8
**5C-O**	35.2°	47.0°	66.8
**5C-S**	50.8°	62.2°	31.5
**5C-Se**	56.2°	67.4°	23.1
**5C-Te**	63.6°	73.8°	13.9

^1^ In kcal mol^−1^.

**Table 2:** Inversion barriers of hetero[5]circulenes. Inversion barriers shown in hyphen (-) indicate that the transition states are unable to be optimized.

	Period 2		Period 3		Period 4		Period 5	
	Label	Inversion Barrier ^1^	Label	Inversion Barrier ^1^	Label	Inversion Barrier ^1^	Label	Inversion Barrier ^1^
C group	**5C-** **C**	42.1	**5C-Si**	14.9	**5C-Ge**	13.1	**5C-Sn**	7.1
	**5C-2** **C_1,2_**	83.1	**5C-2Si_1,2_**	16.6	**5C-2Ge_1,2_**	12.6	**5C-2Sn_1,2_**	3.5
	**5C-2** **C_1,3_**	87.4	**5C-2Si_1,3_**	19.3	**5C-2Ge_1,3_**	15.2	**5C-2Sn_1,3_**	4.5
	**5C-3** **C_1,2,3_**	-	**5C-3Si_1,2,3_**	19.0	**5C-3Ge_1,2,3_**	12.8	**5C-3Sn_1,2,3_**	1.5
	**5C-3** **C_1,2,4_**	-	**5C-3Si_1,2,4_**	22.3	**5C-3Ge_1,2,4_**	15.6	**5C-3Sn_1,2,4_**	2.2
	**5C-4** **C**	-	**5C-4Si**	22.8	**5C-4Ge**	14.4	**5C-4Sn**	0.1
	**5C-5** **C**	-	**5C-5Si**	22.6	**5C-5Ge**	12.2	**5C-5Sn**	planar
N group	**5C-** **N**	59.4	**5C-** **P**	23.4	**5C-As**	17.5	**5C-Sb**	9.8
	**5C-2** **N_1,2_**	-	**5C-2** **P_1,2_**	34.8	**5C-2As_1,2_**	21.2	**5C-2Sb_1,2_**	7.3
	**5C-2** **N_1,3_**	131.2	**5C-2** **P_1,3_**	37.5	**5C-2As_1,3_**	24.4	**5C-2Sb_1,3_**	9.1
	**5C-3** **N_1,2,3_**	-	**5C-3** **P_1,2,3_**	48.1	**5C-3As_1,2,3_**	26.0	**5C-3Sb_1,2,3_**	6.0
	**5C-3** **N_1,2,4_**	-	**5C-3** **P_1,2,4_**	50.9	**5C-3As_1,2,4_**	29.1	**5C-3Sb_1,2,4_**	7.4
	**5C-4** **N**	-	**5C-4** **P**	63.0	**5C-4As**	31.9	**5C-4Sb**	5.3
	**5C-5** **N**	-	**5C-5** **P**	74.9	**5C-5As**	34.7	**5C-5Sb**	3.8
O group	**5C-** **O**	66.8	**5C-** **S**	31.5	**5C-Se**	23.1	**5C-Te**	13.9
	**5C-2** **O_1,2_**	-	**5C-2** **S_1,2_**	56.6	**5C-2Se_1,2_**	35.5	**5C-2Te_1,2_**	15.6
	**5C-2** **O_1,3_**	-	**5C-2** **S_1,3_**	57.2	**5C-2Se_1,3_**	36.8	**5C-2Te_1,3_**	16.4
	**5C-3** **O_1,2,3_**	-	**5C-3** **S_1,2,3_**	83.7	**5C-3Se_1,2,3_**	48.7	**5C-3Te_1,2,3_**	17.1
	**5C-3** **O_1,2,4_**	-	**5C-3** **S_1,2,4_**	85.2	**5C-3Se_1,2,4_**	50.1	**5C-3Te_1,2,4_**	17.8
	**5C-4** **O**	-	**5C-4** **S**	-	**5C-4Se**	62.5	**5C-4Te**	18.5
	**5C-5** **O**	-	**5C-5** **S**	-	**5C-5Se**	73.7	**5C-5Te**	18.6

^1^ In kcal mol^−1^.

## Data Availability

Data are contained within the article.

## Supplementary Materials

The following supporting information can be downloaded at: https://www.mdpi.com/article/10.3390/molecules29225335/s1, Figure S1: 3D structures of positively curved hetero[5]circulenes and their inversion barriers (in kcal mol^−1^); Figure S2: Isomers of **5C-P**, **5C-As** and **5C-Sb**; Table S1: Benchmark study for 11 different levels and experimental data; Table S2: Curvature and curvature radius of positively curved hetero[5]circulenes and inversion barriers (in kcal mol^−1^); Table S3: Curvature and curvature radius of positively curved hetero[6]circulenes and inversion barriers (in kcal mol^−1^). References [30,48,49,50,51,52,53,56,57,60] are cited in Supplementary Materials.
