# Supplementary material for: A Theoretical Study of Positively Curved Circulenes Embedded with Five-Membered Heterocycles: Structures and Inversions
Source: Molecules. 2024 Nov 13;29(22):5335. doi: 10.3390/molecules29225335 (PMC11596064; doi:10.3390/molecules29225335)
Supplement: Supplementary file 1 [file molecules-29-05335-s001.zip › molecules-3271781-supplementary.pdf]

A Theoretical Study of Positively Curved Circulenes Embedded with Five-Membered Heterocycles: Structures and Inversions

Yijian Ma, Tianle Dai and Chengshuo Shen

Supplementary Materials 2
1. Benchmarks 2
2. 3D structures of positively curved hetero[5]circulenes and their inversion barriers (in kcal mol^{-1}). 3
3. Isomers of 5C-P, 5C-As and 5C-Sb. 11
4. Curvature and curvature radius of positively curved hetero[5]circulenes and hetero[6]circulenes. 13
5. Cartesian coordinates of the optimized geometries (in Å) 17
6. Reference 57

Supplementary Materials

1. Benchmarks

Firstly, in order to further investigate the influence of different exchange-correlation functionals and basis sets, we conducted a benchmark study with 11 different levels and experimental data on corannulene, details were listed in Tabel S1. The results showed that, Grimme's r^2SCAN-3c [1], which combined with r^2SCAN functional [2], D4 dispersion correction [3–5], geometrical counter-poise (gcp) corrections [6] and mTZVPP basis set [1], has a very good cost-benefit-ratio for this study. This study was performed using ORCA 5.0.4 program [7,8] with the RIJCOSX method [9].

Table S1. Benchmark study for 11 different levels and experimental data.

Optimization level	Single point level	ΔG_{inv} (kcal mol^{-1})	Total time (min)
Exp. [1]		11.5	-
ωB97M-V/def2-TZVPP		12.0	511
r^2SCAN-3c		11.1	21
B3LYP-D4/def2-TZVPP		11.3	86
PBE0-D4/def2-SVP	PBE0-D4/def2-TZVPP	11.7	24
	ωB97M-V/def2-TZVPP	11.3	25
B3LYP-D4/def2-SVP	B3LYP-D4/def2-TZVPP	12.1	24
	ωB97M-V/def2-TZVPP	11.4	25
M06-2X-D3/def2-SVP	M06-2X-D3/def2-TZVPP	11.3	26
	ωB97M-V/def2-TZVPP	11.0	27
ωB97X-D3/ def2-SVP	ωB97X-D3/ def2-TZVPP	10.8	24
	ωB97M-V/def2-TZVPP	10.9	25

[1] Experimental data from Ref. [10].

2. 3D structures of positively curved hetero[5]circulenes and their inversion barriers (in kcal mol^{-1}).

Figure S1. 3D structures of positively curved hetero[5]circulenes and their inversion barriers (in kcal mol^{-1}).

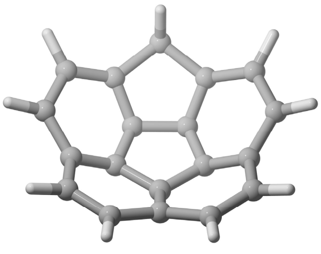

5C-C
42.1

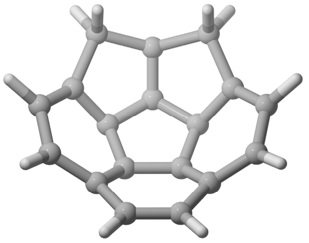

5C-2C$_{1,2}$
83.1

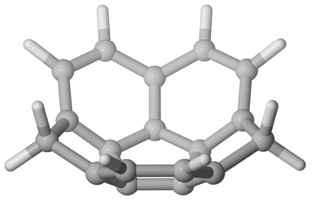

5C-2C$_{1,3}$
87.4

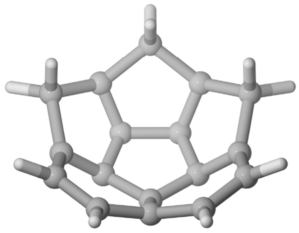

5C-3C$_{1,2,3}$
-

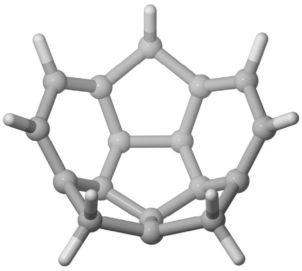

5-3C$_{1,2,4}$
-

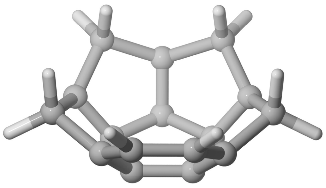

5C-4C
--

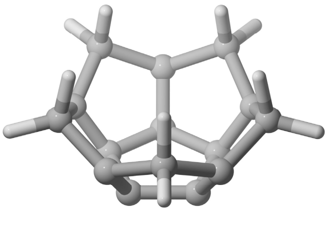

5C-5C
-

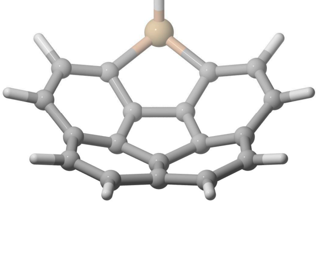

5C-Si
14.9

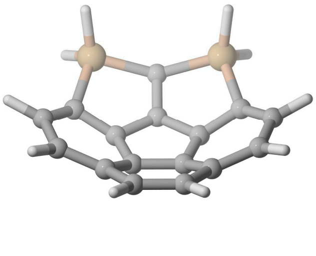

5C-2Si$_{1,2}$
16.6

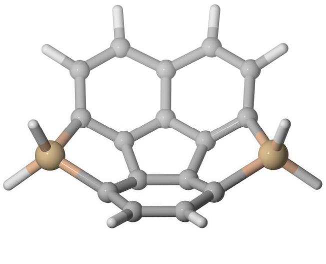

5C-2Si$_{1,3}$
19.3

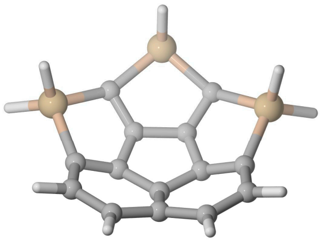

5C-3Si$_{1,2,3}$
19.0

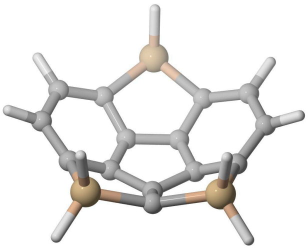

5-3Si$_{1,2,4}$
22.3

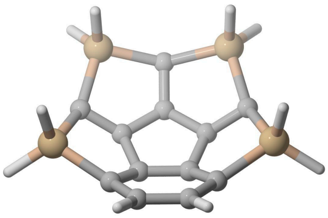

5C-4Si
22.8

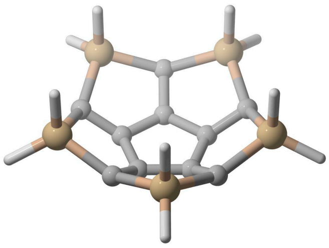

5C-5Si
22.6

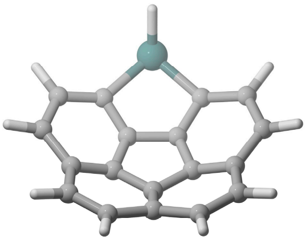

5C-Ge
13.1

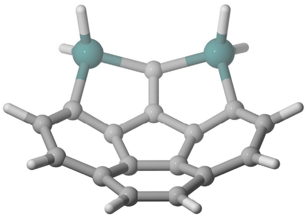

5C-2Ge$_{1,2}$
12.6

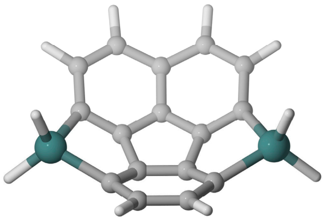

5C-2Ge$_{1,3}$
15.2

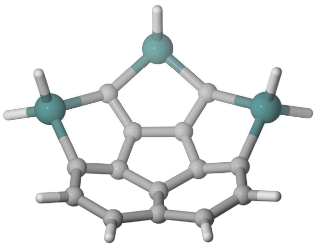

5C-3Ge$_{1,2,3}$
12.8

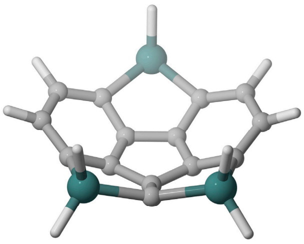

5-3Ge$_{1,2,4}$
15.6

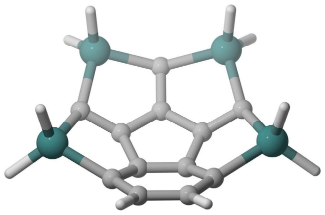

5C-4Ge
14.4

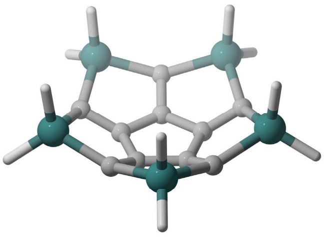

5C-5Ge
12.2

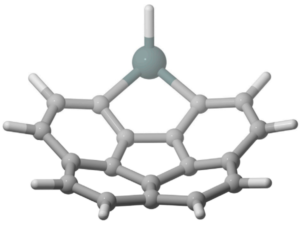

5C-Sn
7.1

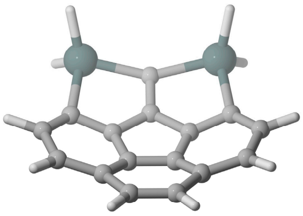

5C-2Sn$_{1,2}$
3.5

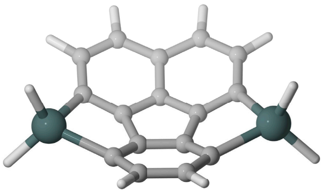

5C-2Sn$_{1,3}$
4.5

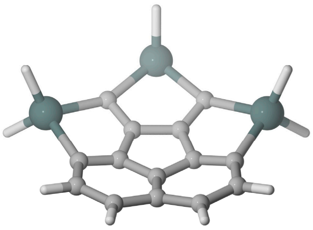

5C-3Sn$_{1,2,3}$
1.5

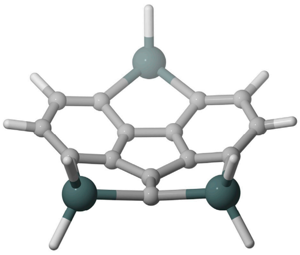

5-3Sn$_{1,2,4}$
2.2

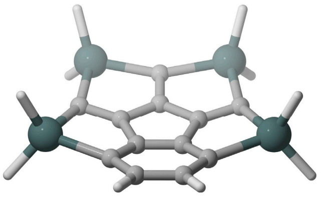

5C-4Sn
0.1

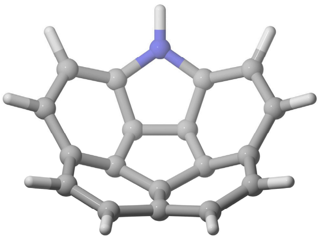

5C-N
59.4

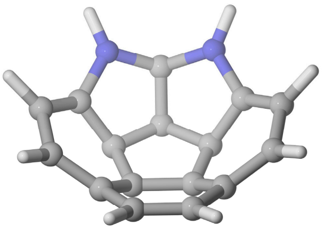

5C-2N$_{1,2}$
-

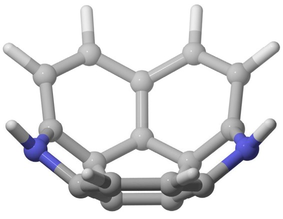

5C-2N$_{1,3}$
131.2

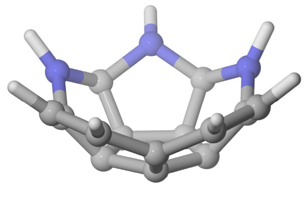

5C-3N$_{1,2,3}$

-

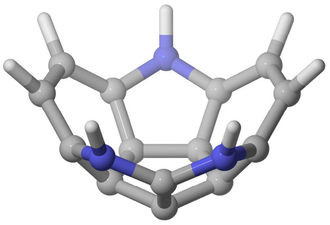

5-3N$_{1,2,4}$

-

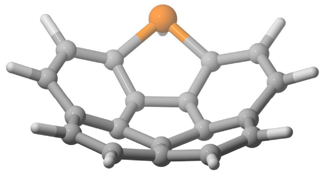

5C-P

23.4

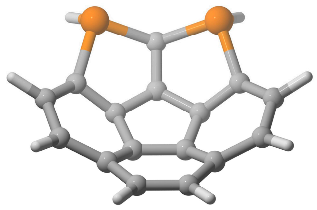

5C-2P$_{1,2}$

34.8

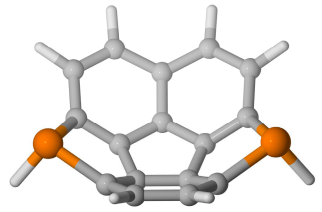

5C-2P$_{1,3}$

37.5

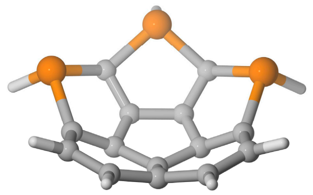

5C-3P$_{1,2,3}$

48.1

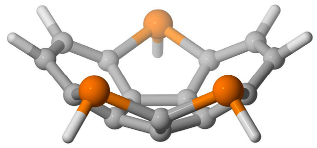

5-3P$_{1,2,4}$

50.9

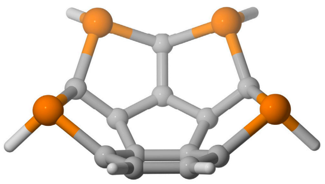

5C-4P

63.0

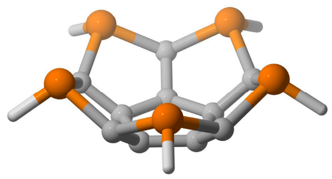

5C-5P

74.9

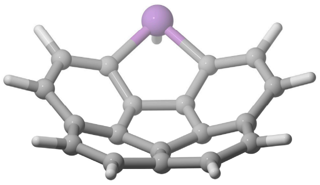

5C-As

17.5

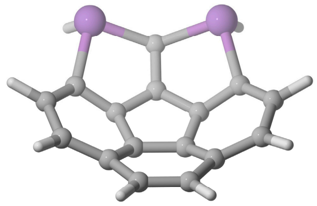

5C-2As$_{1,2}$

21.2

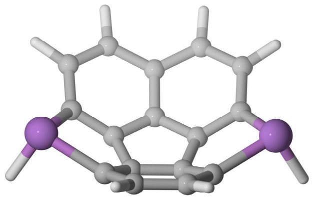

5C-2As$_{1,3}$

24.4

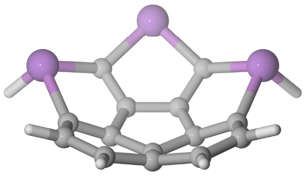

5C-3As$_{1,2,3}$

26.0

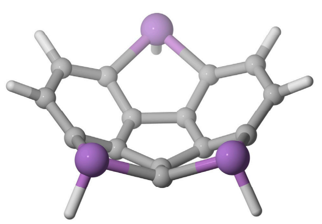

5-3As$_{1,2,4}$

29.1

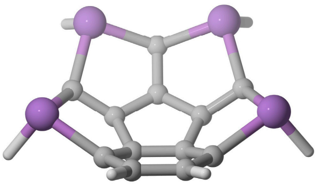

5C-4As

31.9

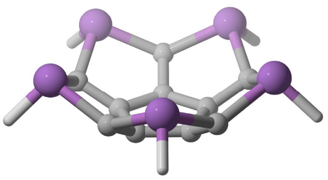

5C-5As

34.7

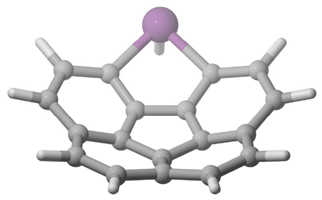

5C-Sb

9.8

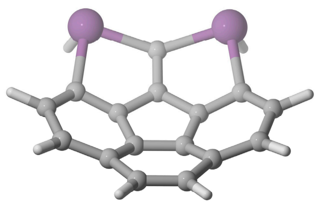

5C-2Sb$_{1,2}$

7.3

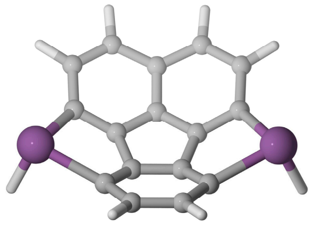

5C-2Sb$_{1,3}$

9.1

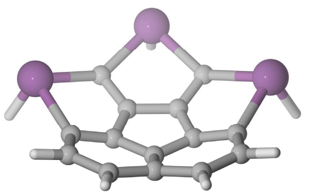

5C-3Sb$_{1,2,3}$

6.0

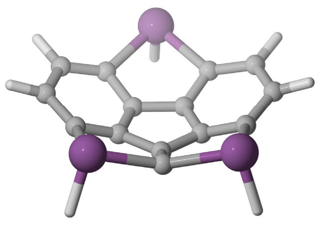

5-3Sb$_{1,2,4}$

7.4

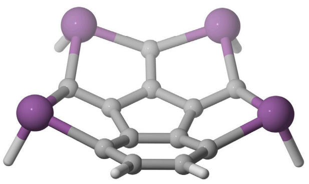

5C-4Sb

5.3

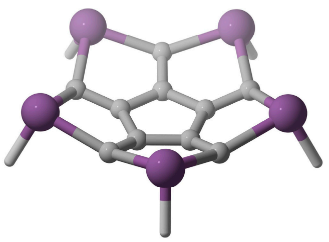

5C-5Sb

3.8

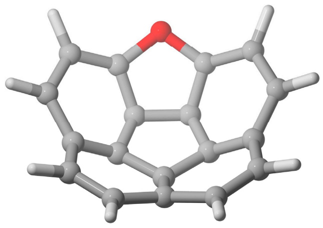

5C-O

66.8

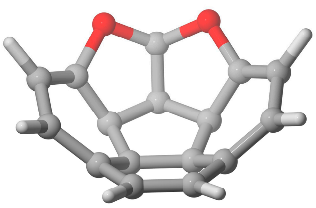

5C-2O$_{1,2}$

-

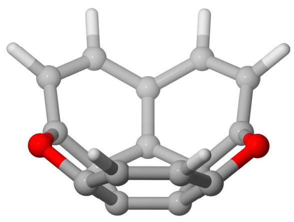

5C-2O$_{1,3}$

-

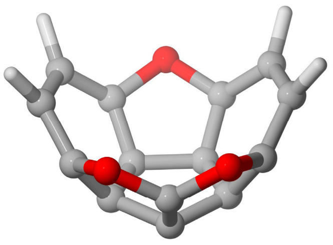

5-3O$_{1,2,4}$

-

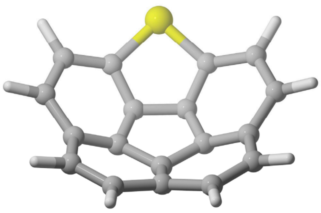

5C-S
31.5

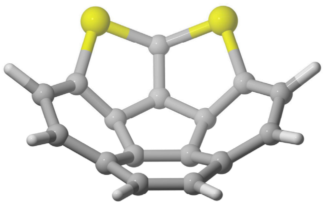

5C-2S$_{1,2}$
56.6

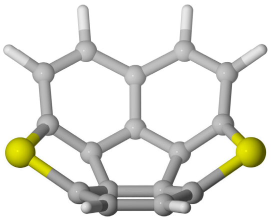

5C-2S$_{1,3}$
57.2

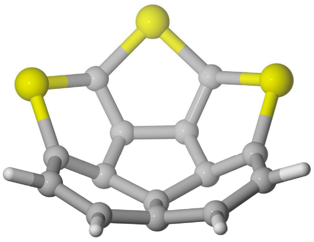

5C-3S$_{1,2,3}$
83.7

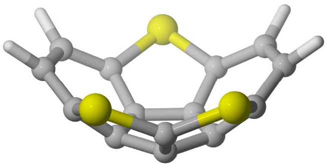

5-3S$_{1,2,4}$
85.2

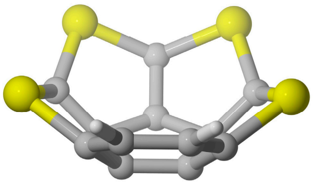

5C-4S
-

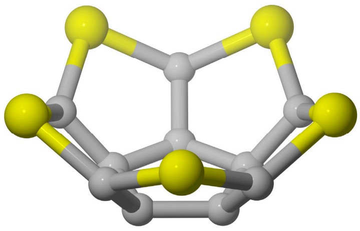

5C-5S
-

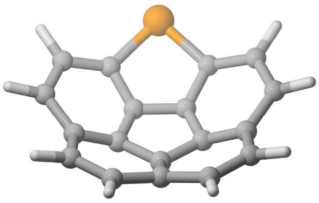

5C-Se
23.1

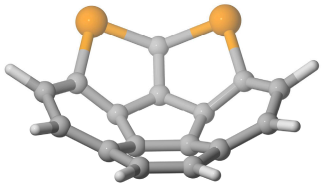

5C-2Se$_{1,2}$
35.5

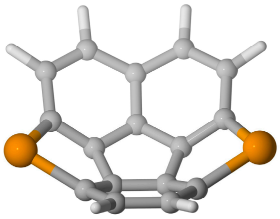

5C-2Se$_{1,3}$
36.8

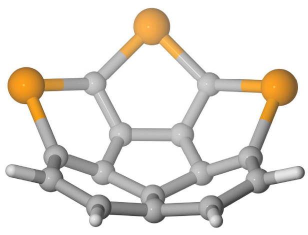

5C-3Se$_{1,2,3}$

48.7

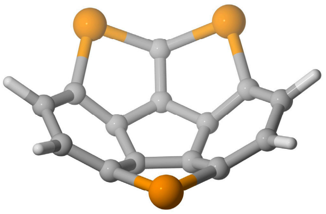

5-3Se$_{1,2,4}$

50.1

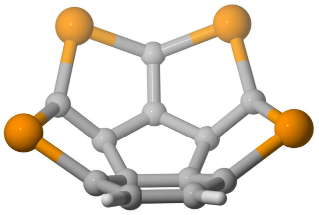

5C-4Se

62.5

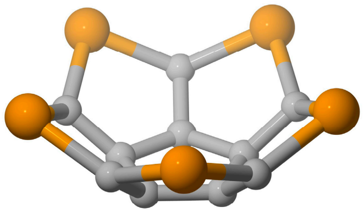

5C-5Se

73.7

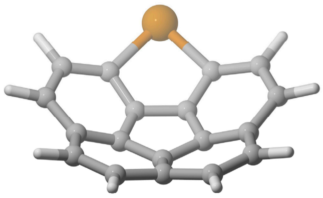

5C-Te

13.9

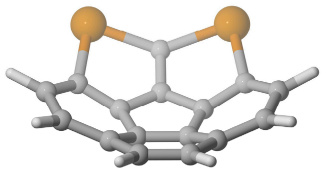

5C-2Te$_{1,2}$

15.6

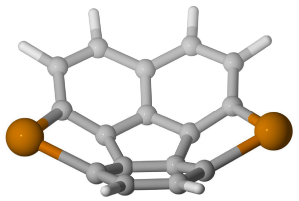

5C-2Te$_{1,3}$

16.4

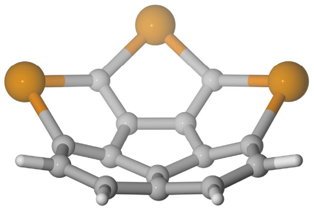

5C-3Te$_{1,2,3}$

17.1

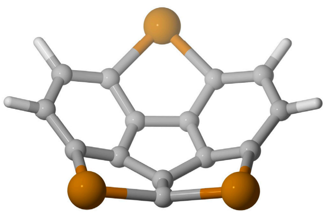

5-3Te$_{1,2,4}$

17.8

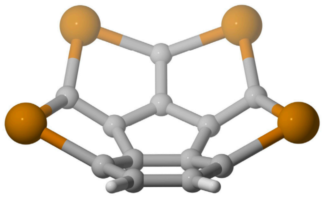

5C-4Te

18.5

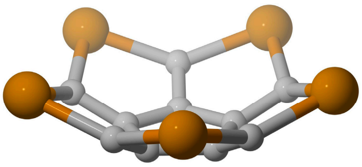

5C-5Se
18.6

3. Isomers of 5C-P, 5C-As and 5C-Sb.

Figure S2. Isomers of **5C-P**, **5C-As** and **5C-Sb**.

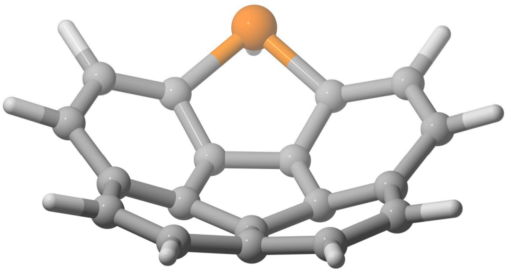

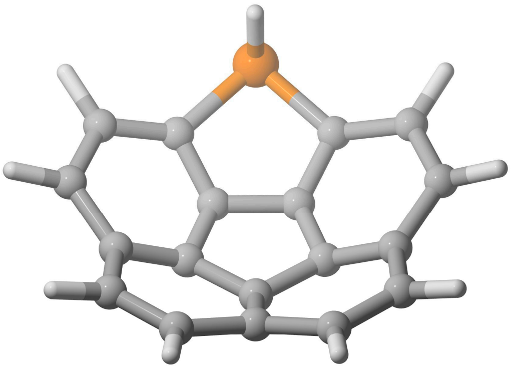

+0.38 kcal mol^{-1}

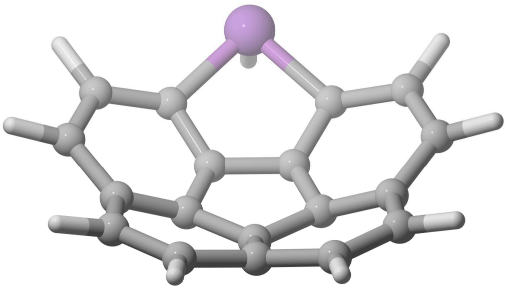

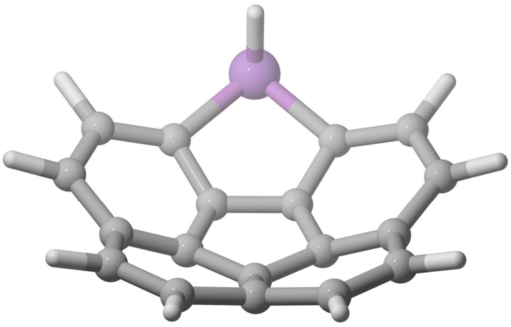

+0.35 kcal mol^{-1}

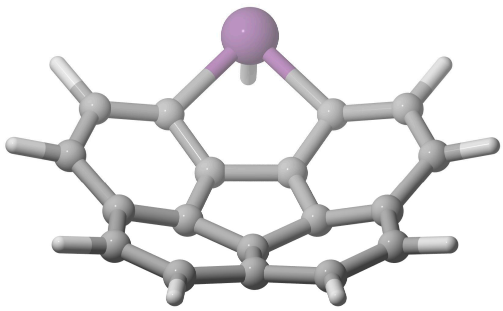

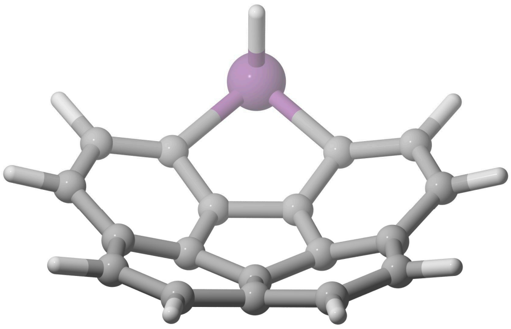

+ 0.22 kcal mol^{-1}

4. Curvature and curvature radius of positively curved hetero[5]circulenes and hetero[6]circulenes.

Table S2. Curvature and curvature radius of positively curved hetero[5]circulenes and inversion barriers (in kcal mol^{-1}).

	R (Å)	K (Å^{-1})	ΔG_{inv} (kcal mol^{-1})
**5C**	5.47	0.183	11.1
**5C-C**	3.96	0.253	42.1
**5C-2C$_{1,2}$**	3.27	0.306	83.1
**5C-2C$_{1,3}$**	3.22	0.311	87.4
**5C-3C$_{1,2,3}$**	2.87	0.348	- [1]
**5C-3C$_{1,2,4}$**	2.78	0.360	-
**5C-4C**	2.52	0.396	-
**5C-5C**	2.32	0.430	-
**5C-Si**	5.05	0.198	14.9
**5C-2Si$_{1,2}$**	4.79	0.209	16.6
**5C-2Si$_{1,3}$**	4.59	0.218	19.3
**5C-3Si$_{1,2,3}$**	4.50	0.222	19.0
**5C-3Si$_{1,2,4}$**	4.29	0.233	22.3
**5C-4Si**	4.13	0.242	22.8
**5C-5Si**	4.01	0.249	22.6
**5C-Ge**	5.22	0.191	13.1
**5C-2Ge$_{1,2}$**	5.01	0.199	12.6
**5C-2Ge$_{1,3}$**	4.88	0.205	15.2
**5C-3Ge$_{1,2,3}$**	4.99	0.200	12.8
**5C-3Ge$_{1,2,4}$**	4.71	0.212	15.6
**5C-4Ge**	4.72	0.212	14.4
**5C-5Ge**	4.78	0.209	12.2
**5C-Sn**	5.99	0.167	7.1
**5C-2Sn$_{1,2}$**	7.07	0.141	3.5
**5C-2Sn$_{1,3}$**	6.54	0.153	4.5
**5C-3Sn$_{1,2,3}$**	8.72	0.115	1.5
**5C-3Sn$_{1,2,4}$**	7.63	0.131	2.2
**5C-4Sn**	11.82	0.085	0.1
**5C-N**	3.71	0.269	59.4
**5C-2N$_{1,2}$**	3.00	0.333	-
**5C-2N$_{1,3}$**	3.01	0.332	131.2
**5C-3N$_{1,2,3}$**	2.60	0.385	-
**5C-3N$_{1,2,4}$**	2.59	0.386	-
**5C-P**	4.55	0.220	23.4
**5C-2P$_{1,2}$**	4.00	0.250	34.8
**5C-2P$_{1,3}$**	3.96	0.253	37.5
**5C-3P$_{1,2,3}$**	3.60	0.278	48.1
**5C-3P$_{1,2,4}$**	3.56	0.281	50.9
**5C-4P**	3.29	0.304	63.0

**5C-5P**	3.08	0.324	74.9
**5C-As**	4.84	0.206	17.5
**5C-2As**$_{1,2}$	4.45	0.224	21.2
**5C-2As**$_{1,3}$	4.35	0.230	24.4
**5C-3As**$_{1,2,3}$	4.11	0.243	26.0
**5C-3As**$_{1,2,4}$	4.02	0.249	29.1
**5C-4As**	3.80	0.263	31.9
**5C-5As**	3.64	0.275	34.7
**5C-Sb**	5.54	0.180	9.8
**5C-2Sb**$_{1,2}$	5.74	0.174	7.3
**5C-2Sb**$_{1,3}$	5.49	0.182	9.1
**5C-3Sb**$_{1,2,3}$	5.80	0.172	6.0
**5C-3Sb**$_{1,2,4}$	5.58	0.179	7.4
**5C-4Sb**	5.82	0.172	5.3
**5C-5Sb**	6.12	0.163	3.8
**5C-O**	3.59	0.279	66.8
**5C-2O**$_{1,2}$	2.85	0.350	-
**5C-2O**$_{1,3}$	2.88	0.347	-
**5C-3O**$_{1,2,4}$	2.46	0.406	-
**5C-S**	4.29	0.233	31.5
**5C-2S**$_{1,2}$	3.68	0.272	56.6
**5C-2S**$_{1,3}$	3.67	0.272	57.2
**5C-3S**$_{1,2,3}$	3.30	0.303	83.7
**5C-3S**$_{1,2,4}$	3.27	0.305	85.2
**5C-4S**	3.02	0.331	-
**5C-5S**	2.82	0.355	-
**5C-Se**	4.60	0.217	23.1
**5C-2Se**$_{1,2}$	4.11	0.243	35.5
**5C-2Se**$_{1,3}$	4.06	0.246	36.8
**5C-3Se**$_{1,2,3}$	3.77	0.265	48.7
**5C-3Se**$_{1,2,4}$	3.72	0.269	50.1
**5C-4Se**	3.50	0.286	62.5
**5C-5Se**	3.32	0.301	73.7
**5C-Te**	5.02	0.199	13.9
**5C-2Te**$_{1,2}$	4.83	0.207	15.6
**5C-2Te**$_{1,3}$	4.92	0.203	16.4
**5C-3Te**$_{1,2,3}$	4.87	0.205	17.1
**5C-3Te**$_{1,2,4}$	4.78	0.209	17.8
**5C-4Te**	4.73	0.212	18.5
**5C-5Te**	4.72	0.212	18.6

[1] Inversion barriers shown in hyphen (-) indicate that the transition states are unable to be optimized.

Table S3. Curvature and curvature radius of positively curved hetero[6]circulenes and inversion barriers (in kcal mol^{-1}).

	R (Å)	K ($Å^{-1}$)	ΔG_{inv} (kcal mol^{-1})
**6C-2$C_{1,4}$**	8.56	0.117	2.6
**6C-2$C_{1,3}$**	7.98	0.125	3.1
**6C-2$C_{1,2}$**	7.49	0.134	4.2
**6C-3$C_{1,3,5}$**	4.87	0.205	20.2
**6C-3$C_{1,2,4}$**	4.93	0.203	20.1
**6C-3$C_{1,2,3}$**	4.90	0.204	22.8
**6C-4$C_{1,2,3,4}$**	3.95	0.253	52.8
**6C-4$C_{1,2,3,5}$**	3.86	0.259	53.0
**6C-4$C_{1,2,4,5}$**	3.80	0.263	52.5
**6C-5C**	3.32	0.301	93.9
**6C-6C**	2.96	0.338	137.9
**6C-N**	78.62	0.013	1.2
**6C-2$N_{1,4}$**	6.08	0.164	10.7
**6C-2$N_{1,3}$**	5.97	0.168	11.4
**6C-2$N_{1,2}$**	5.41	0.185	21.8
**6C-3$N_{1,3,5}$**	4.15	0.241	45.3
**6C-3$N_{1,2,4}$**	4.08	0.245	57.2
**6C-3$N_{1,2,3}$**	3.93	0.254	- [1]
**6C-4$N_{1,2,3,4}$**	3.28	0.305	-
**6C-4$N_{1,2,3,5}$**	3.30	0.303	-
**6C-4$N_{1,2,4,5}$**	3.29	0.304	121.4
**6C-5N**	2.83	0.354	-
**6C-6N**	2.51	0.399	-
**6C-4$P_{1,2,3,4}$**	7.23	0.138	4.8
**6C-4$P_{1,2,3,5}$**	7.28	0.137	4.2
**6C-4$P_{1,2,4,5}$**	7.31	0.137	3.9
**6C-5P**	5.68	0.176	10.6
**6C-6P**	4.83	0.207	18.7
**6C-O**	17.91	0.056	1.4
**6C-2$O_{1,4}$**	5.28	0.189	19.3
**6C-2$O_{1,3}$**	5.18	0.193	21.4
**6C-2$O_{1,2}$**	4.70	0.213	33.4
**6C-3$O_{1,3,5}$**	3.74	0.267	72.1
**6C-3$O_{1,2,4}$**	3.66	0.273	83.6
**6C-3$O_{1,2,3}$**	3.54	0.283	-
**6C-4$O_{1,2,3,4}$**	3.01	0.332	-
**6C-4$O_{1,2,3,5}$**	3.01	0.332	-
**6C-4$O_{1,2,4,5}$**	2.99	0.335	-
**6C-5O**	2.62	0.382	-
**6C-6O**	2.33	0.428	-
**6C-2$S_{1,4}$**	57.89	0.017	0.9

**6C-2S$_{1,2}$**	16.47	0.061	1.2
**6C-3S$_{1,3,5}$**	7.85	0.127	4.1
**6C-3S$_{1,2,4}$**	7.43	0.135	5.0
**6C-3S$_{1,2,3}$**	7.10	0.141	5.9
**6C-4S$_{1,2,3,4}$**	5.36	0.187	17.2
**6C-4S$_{1,2,3,5}$**	5.38	0.186	16.3
**6C-4S$_{1,2,4,5}$**	5.33	0.188	16.8
**6C-5S**	4.45	0.225	33.8
**6C-6S**	3.90	0.256	55.1
**6C-4Se$_{1,2,3,4}$**	8.77	0.114	2.9
**6C-4Se$_{1,2,3,5}$**	8.36	0.120	2.9
**6C-4Se$_{1,2,4,5}$**	8.54	0.117	3.1
**6C-5Se**	6.54	0.153	7.4
**6C-6Se**	5.49	0.182	13.6

[1] Inversion barriers shown in hyphen (-) indicate that the transition states are unable to be optimized.

5. Cartesian coordinates of the optimized geometries (in Å)

5C

C	-0.768930	-1.216880	-0.749260
C	0.642140	-1.292040	-0.751910
C	-1.138110	0.075040	-1.187020
C	1.145020	-0.046980	-1.191880
C	0.044720	0.798000	-1.460640
C	2.378950	0.438810	-0.799700
C	0.118610	2.174580	-1.352340
C	1.447930	2.696790	-1.161460
C	2.526140	1.868800	-0.897790
C	1.345880	-2.118940	0.104460
C	-1.553170	-1.964840	0.109800
C	-2.311720	0.689120	-0.789950
C	-2.305970	2.126810	-0.887320
C	-1.146740	2.835380	-1.155580
C	-2.868990	-1.429060	0.349980
C	-3.230750	-0.163030	-0.079150
C	0.537220	-3.055100	0.843230
C	-0.845620	-2.981730	0.845600
C	2.712070	-1.726070	0.339770
C	3.204820	-0.505920	-0.091440
H	1.597880	3.773220	-1.120280
H	3.484400	2.324470	-0.658960
H	-3.209270	2.682040	-0.645140
H	-1.181090	3.921670	-1.114380
H	-3.566680	-1.985290	0.971880
H	-4.199730	0.229610	0.220590
H	1.019950	-3.776410	1.498660
H	-1.399640	-3.648420	1.502510
H	3.348800	-2.353460	0.959280
H	4.211090	-0.218590	0.204730

5C-C

C	-0.279502	2.983255	-0.408664
C	-1.415294	1.927904	-0.545616
C	0.994979	2.135915	-0.691804
C	-0.857752	0.829614	-1.147336
C	0.565068	0.952234	-1.233256
C	-1.176870	-0.497898	-0.832265
C	1.141003	-0.298032	-0.971554
C	0.062123	-1.209069	-0.732588
C	0.206509	-2.272280	0.145550
C	-2.253218	-0.772682	0.012247
C	2.340869	-0.376158	-0.263437
C	1.548415	-2.535184	0.601378
C	2.568560	-1.624499	0.419405
C	-2.182175	-2.034920	0.703786
C	-1.007269	-2.756172	0.754055
C	-2.611196	1.676247	0.174575
C	-3.036844	0.360466	0.399432
C	2.293730	2.099686	-0.122378
C	2.960364	0.877931	0.038781
H	-0.253119	3.392675	0.608476
H	-0.394657	3.832395	-1.096260
H	1.731687	-3.405589	1.227436
H	3.519787	-1.798344	0.917401
H	-3.023653	-2.363084	1.309843
H	-0.963504	-3.638222	1.389033
H	-3.148748	2.479000	0.674149
H	-3.900783	0.207876	1.042607
H	2.741776	2.987404	0.318184
H	3.907511	0.881531	0.573821

5C-2$C_{1,2}$

C	4.601624	1.916271	0.589064
C	4.578519	0.366811	0.358365
C	3.402098	2.399749	-0.270189
C	3.681690	0.136711	-0.666176
C	3.068292	1.369227	-1.063614
C	2.667133	-0.843730	-0.582987
C	1.652617	1.162842	-1.144803
C	1.399397	-0.202720	-0.882465
C	0.990259	2.180932	-0.488310
C	0.242793	-0.563321	-0.180114
C	2.743615	-1.827244	0.411002
C	4.772994	-0.702973	1.257855
C	3.910117	-1.807671	1.228404
C	1.488987	-2.443818	0.785951
C	0.291204	-1.838514	0.502777
C	-0.649200	0.496904	0.151289
C	-0.260847	1.841565	0.069481
C	2.089838	3.185692	-0.003113
H	4.418033	2.118262	1.651497
H	5.551678	2.403450	0.333899
H	5.477132	-0.627378	2.083414
H	4.015985	-2.558976	2.007739
H	1.497737	-3.316274	1.435269
H	-0.620059	-2.246585	0.934021
H	-1.565006	0.262540	0.689169
H	-0.869793	2.580806	0.585033
H	2.058329	4.170161	-0.487710
H	1.959934	3.358781	1.072352

5C-2$C_{1,3}$

C	4.474208	0.492753	-3.794138
C	3.229024	0.265116	-2.896369
C	5.620137	-0.006805	-2.870284
C	3.592527	-0.602874	-1.895942
C	5.027802	-0.810663	-1.907474
C	3.144335	-0.465997	-0.555671
C	5.459731	-0.829530	-0.587190
C	4.296568	-0.633002	0.256906
C	6.821382	0.612730	-2.492130
C	6.513309	-0.045607	-0.140411
C	7.263959	0.593468	-1.139434
C	4.601779	0.207020	1.299617
C	6.138586	0.421971	1.293979
C	2.208297	0.530073	-0.235814
C	2.362634	1.146406	1.049857
C	3.543965	1.024445	1.778999
C	2.099298	1.085786	-2.637246
C	1.578233	1.179915	-1.348180
H	4.420387	-0.061689	-4.740642
H	4.592598	1.553083	-4.040038
H	7.324933	1.300922	-3.167537
H	8.074758	1.268621	-0.874629
H	6.388097	1.476887	1.447763
H	6.649601	-0.155704	2.075834
H	1.636129	1.890094	1.369400
H	3.690822	1.683420	2.631756
H	1.719818	1.767456	-3.395068
H	0.810206	1.925975	-1.157554

5C-3C$_{1,2,3}$

C	3.300777	-0.351060	0.138844
C	4.738274	-0.530671	0.247542
C	2.919883	0.770865	0.845019
C	4.208567	1.358870	1.497650
C	5.328689	0.549906	0.793752
C	2.910225	-0.529310	-1.227257
C	4.099669	-0.799668	-1.976804
C	5.236848	-0.810767	-1.073124
C	6.178160	0.072897	-1.457366
C	4.410319	-0.066911	-3.103477
C	1.934355	0.328951	-1.777505
C	6.441848	0.979291	-0.213906
C	5.884229	0.417074	-2.940650
C	2.096918	0.680865	-3.156520
C	3.331382	0.548393	-3.785457
C	1.749113	1.437642	0.406220
C	1.228599	1.168644	-0.856153
H	4.277027	2.425758	1.254941
H	4.241523	1.279982	2.592423
H	7.467222	0.967943	0.177212
H	6.238997	2.017965	-0.510115
H	6.594818	-0.044128	-3.639375
H	5.926898	1.498771	-3.113396
H	1.333674	1.285790	-3.640285
H	3.486628	1.073377	-4.725534
H	1.340428	2.279573	0.960841
H	0.422140	1.797116	-1.226506

5C-3C$_{1,2,4}$

C	1.583849	0.192466	-1.202054
C	2.782256	-0.396144	-0.633911
C	0.782664	0.678028	-0.169090
C	1.414836	0.171962	1.170107
C	2.822722	-0.266971	0.705568
C	1.993173	1.027551	-2.248033
C	3.462173	1.032735	-2.292895
C	3.939808	0.201124	-1.273687
C	4.798939	0.691860	-0.291085
C	3.909438	2.345363	-2.436801
C	1.528516	2.336537	-2.365024
C	4.253597	0.181033	1.083631
C	2.700904	3.197015	-2.904280
C	0.128702	1.893764	-0.398197
C	0.494530	2.713314	-1.499800
H	1.477567	1.000056	1.885133
H	0.847897	-0.631534	1.658884
H	4.854128	-0.620094	1.534825
H	4.229987	1.007478	1.802866
H	-0.473850	2.352929	0.382487
H	0.125464	3.736850	-1.500471
C	4.991363	2.729225	-1.635682
C	5.429190	1.912031	-0.558911
H	5.352407	3.755021	-1.657786
H	6.075370	2.375466	0.183452
H	2.710444	4.196599	-2.460620
H	2.667395	3.310116	-3.996176

5C-4C

C	4.138546	0.142260	0.664353
C	2.712005	0.359605	0.914506
C	4.821982	1.342818	0.927505
C	3.803076	2.320195	1.583546
C	2.461482	1.663486	1.180410
C	4.294717	-0.506085	-0.572991
C	2.973681	-0.729037	-1.165846
C	2.000622	-0.122202	-0.264170

C	1.415415	0.920843	-0.883552
C	2.915051	-0.219582	-2.418959
C	5.136045	0.040031	-1.558609
C	1.451287	2.130972	0.099956
C	4.370263	-0.034104	-2.912262
C	5.876938	1.682330	0.088572
C	6.035400	1.024612	-1.166323
H	3.917635	2.414861	2.671571
H	3.913413	3.323828	1.160017
H	1.876878	3.002619	-0.415144
H	0.462354	2.443383	0.461500
H	6.397253	2.629308	0.215521
H	6.664079	1.519058	-1.903968
C	1.768616	0.825564	-2.399742
H	4.702444	-0.852002	-3.565302
H	4.499423	0.898082	-3.471694
H	0.907847	0.615909	-3.048782
H	2.178076	1.788337	-2.733613

5C-5C

C	0.334738	1.649808	1.345101
C	-0.935481	0.904044	1.331609
C	1.435908	0.674160	1.270238
C	-0.619334	-0.532382	1.249185
C	0.846610	-0.674627	1.211223
C	0.390677	2.504466	0.299802
C	-1.691280	1.281023	0.276725
C	2.195497	0.904410	0.176450
C	1.229421	-1.306946	0.079830
C	-1.173364	-1.073448	0.141727
C	-2.128085	-0.010750	-0.464529
C	-0.054453	-1.850495	-0.602632
C	2.338819	-0.443930	-0.579121
C	1.744016	2.263647	-0.421645
C	-1.018716	2.531542	-0.350514
H	-1.918948	0.096425	-1.535898
H	-0.053555	-1.563944	-1.661383
H	2.102337	-0.294433	-1.639710
H	1.568164	2.148742	-1.498231
H	-0.923130	2.389980	-1.433931
H	-3.197930	-0.250546	-0.386249
H	-0.160333	-2.944404	-0.584756
H	3.345389	-0.884751	-0.552305
H	2.473954	3.079107	-0.319348
H	-1.572754	3.471200	-0.215270

5C-Si

Si	-0.138422	-2.937289	0.482962
C	-1.632747	-1.747205	0.248364
C	1.016932	-1.983625	-0.725225
C	-1.213389	-0.970990	-0.807060
C	0.144856	-1.092058	-1.305993
C	-1.636390	0.348613	-1.031229
C	0.523698	0.156146	-1.824647
C	-0.566189	1.048185	-1.647945
C	-0.382532	2.408352	-1.477846
C	-2.552394	0.973430	-0.192376
C	1.847318	0.581422	-1.808758
C	0.959441	2.879602	-1.716826
C	2.023342	2.011053	-1.871959
C	-2.502240	2.414212	-0.209713
C	-1.469224	3.095867	-0.824664
C	-2.706606	-1.183656	1.000163
C	-3.162451	0.118443	0.777416
C	2.393235	-1.638264	-0.874070
C	2.794802	-0.412495	-1.411583
H	-0.404931	-4.347126	0.099392
H	0.375551	-2.915634	1.876689
H	1.163519	3.946676	-1.664929
H	3.027748	2.423483	-1.933164
H	-3.230585	2.981175	0.365422
H	-1.422627	4.176981	-0.715015
H	-3.131347	-1.729239	1.839171
H	-3.911918	0.525262	1.452416
H	3.166305	-2.290380	-0.475112
H	3.854344	-0.167241	-1.400981

5C-2Si$_{1,2}$

Si	-5.505425	-0.891212	0.292863
C	-3.824017	-1.732471	0.724112
C	-5.026770	-0.391667	-1.487318
C	-3.195089	-1.714696	-0.500928
C	-3.836514	-1.031894	-1.615055
C	-1.806818	-1.599799	-0.673625
C	-2.772074	-0.533295	-2.473818
C	-1.548239	-0.878703	-1.879039
C	-2.982174	0.617153	-3.200753
C	-0.416779	-0.083287	-2.035698
C	-0.942791	-1.552177	0.416405
C	-2.941385	-1.869178	1.831692
C	-1.551761	-1.805856	1.680879
C	0.347490	-0.958994	0.142846
C	0.596846	-0.262622	-1.019747
C	-0.553325	0.982830	-2.973249
C	-1.794351	1.334137	-3.515790
Si	-4.837749	0.971205	-2.811800

H	-6.700576	-1.767022	0.396231
H	-5.719530	0.302139	1.152778
H	-3.335043	-1.920254	2.843933
H	-0.936052	-1.813108	2.577498
H	1.109334	-0.965591	0.918989
H	1.546934	0.256842	-1.121710
H	0.296751	1.632154	-3.170038
H	-1.844294	2.244687	-4.108007
H	-5.765600	0.858139	-3.965935
H	-4.987630	2.325430	-2.217314

5C-2Si$_{1,3}$

Si	4.614702	0.711533	-4.264928
C	3.172383	0.358955	-3.042333
C	5.990192	0.255384	-3.002465
C	3.807782	-0.273844	-1.999404
C	5.262946	-0.339820	-1.982211
C	3.348631	-0.231731	-0.665169
C	5.672781	-0.331527	-0.649863
C	4.478101	-0.261158	0.181056
C	7.265658	0.720315	-2.615495
C	6.845352	0.273146	-0.221336
C	7.681126	0.729123	-1.263560
C	4.535781	0.385102	1.393939
Si	6.414903	0.746460	1.590961
C	2.195116	0.473637	-0.314978
C	2.156614	0.942466	1.037631
C	3.288632	0.914175	1.848598
C	1.883284	0.886991	-2.723985
C	1.402168	0.927829	-1.417531
H	4.596188	-0.122608	-5.493198
H	4.667479	2.141568	-4.661975
H	7.913250	1.211023	-3.338274
H	8.620845	1.226547	-1.035238
H	6.676029	2.180160	1.877034
H	7.092572	-0.075613	2.625184
H	1.271847	1.461323	1.399198
H	3.228211	1.407906	2.815678
H	1.288568	1.370527	-3.495450
H	0.465698	1.446243	-1.225396

5C-3Si$_{1,2,3}$

C	4.362085	-0.960367	-1.115948
C	5.780113	-1.011454	-0.779840
C	3.981701	-0.299266	-2.260175
Si	5.692774	0.159677	-3.026631
C	6.731906	-0.402079	-1.531343
C	3.617824	-0.941497	0.084505
C	4.499867	-1.007468	1.185574
C	5.867823	-1.042572	0.681521
C	6.903555	-0.462911	1.339432
C	4.259617	-0.395377	2.393254
C	2.398847	-0.255408	0.171213
Si	7.956409	0.107309	-0.152623
Si	6.050518	0.035259	2.969385
C	2.028933	0.155014	1.490917
C	2.933435	0.106635	2.550517
C	2.648512	0.207628	-2.237425
C	1.876706	0.210008	-1.076572
H	6.015455	-0.514396	-4.309675
H	5.775526	1.629301	-3.230077
H	8.069399	1.590064	-0.127179
H	9.320669	-0.470468	-0.247459
H	6.160803	1.495743	3.220587
H	6.522070	-0.689103	4.176640
H	1.074099	0.651356	1.647478
H	2.633261	0.569005	3.488057
H	2.241129	0.709171	-3.112249
H	0.911824	0.711936	-1.097047

5C-3Si$_{1,2,4}$

C	4.776392	-0.966226	1.134126
C	3.532382	-1.143431	1.880078
C	5.928302	-0.658218	1.844032
Si	5.329442	-0.850517	3.668461
C	3.484736	-1.029830	3.233612
C	4.460783	-0.388073	-0.094982
C	3.012711	-0.196058	-0.161249
C	2.448623	-0.658353	1.027521
C	1.356983	-0.053734	1.634647
C	2.510406	0.898455	-0.855510
C	5.289820	0.530208	-0.728698
Si	1.714712	-0.380562	3.501376
Si	4.059605	1.582793	-1.761982
C	6.862140	0.126018	1.136681
C	6.548758	0.702918	-0.115902
H	5.920608	-1.988469	4.417197
H	5.591546	0.400982	4.425742
H	0.786992	-1.330722	4.166162
H	1.707733	0.896081	4.261930
H	7.794658	0.429969	1.606241
H	7.265840	1.407785	-0.530832
C	1.289743	1.398976	-0.356137
C	0.726113	0.937595	0.855962
H	0.820564	2.261100	-0.824986
H	-0.133931	1.478504	1.243555

H	4.088939	1.306008	-3.220856
H	4.243881	3.040562	-1.548221

5C-4Si

C	3.633610	-1.275188	-2.708874
C	5.038661	-0.880973	-2.762929
C	2.677762	-0.276760	-2.890248
Si	3.765246	1.236915	-3.395338
C	5.421920	0.405078	-2.979856
C	3.476640	-2.312328	-1.791132
C	4.772294	-2.642838	-1.204200
C	5.756126	-1.735507	-1.808254
C	6.789909	-1.248309	-1.080893
C	4.898792	-3.058621	0.083774
C	2.350722	-2.438413	-0.978932
Si	6.962173	0.494124	-1.859158
Si	3.082799	-3.279962	0.597942
C	1.463682	-0.498480	-2.221749
C	1.303711	-1.555836	-1.286949
H	3.479896	2.390664	-2.503610
H	3.605941	1.679962	-4.804146
H	6.740780	1.517982	-0.803570
H	8.258802	0.772274	-2.526799
H	0.661606	0.233707	-2.282743
H	0.390048	-1.560416	-0.696621
Si	6.553479	-2.230962	0.547037
H	2.645752	-4.682225	0.818194
H	2.743788	-2.483978	1.805950
H	6.329991	-1.273727	1.662830
H	7.671298	-3.137054	0.913648

5C-5Si

C	0.324073	1.680269	0.920515
C	-0.943855	0.938081	0.908084
C	1.421401	0.705823	0.848996
C	-0.630271	-0.495068	0.828767
C	0.831622	-0.638769	0.792445
C	0.427947	2.891537	0.318690
C	-2.042292	1.445724	0.294961
C	2.565539	0.993318	0.179457
C	1.416778	-1.626067	0.069267
C	-1.431057	-1.346275	0.140104
Si	-2.843696	-0.160442	-0.358399
Si	-0.129774	-2.571166	-0.534813
Si	3.006203	-0.734830	-0.504662
Si	2.230220	2.809167	-0.309458
Si	-1.385141	3.164292	-0.218420
H	-2.915198	-0.093932	-1.841941
H	-0.163838	-2.540709	-2.020934
H	3.016518	-0.677016	-1.990309
H	2.228576	2.915405	-1.792460
H	-1.436674	3.276606	-1.700015
H	-4.193027	-0.484977	0.168175
H	-0.257360	-3.980534	-0.086677
H	4.291184	-1.317599	-0.043598
H	3.166061	3.822971	0.238002
H	-2.077240	4.337591	0.371223

5C-Ge

C	-1.655067	-1.735653	0.255608
C	1.040140	-1.976017	-0.734653
C	-1.205506	-0.963479	-0.786339
C	0.153508	-1.084611	-1.285514
C	-1.626711	0.357455	-1.012157
C	0.530133	0.165291	-1.804429
C	-0.558518	1.056777	-1.629207
C	-0.379620	2.418737	-1.472448
C	-2.553888	0.985428	-0.188527
C	1.852854	0.592821	-1.807552
C	0.960270	2.890920	-1.721348
C	2.024427	2.022980	-1.879254
C	-2.505697	2.426520	-0.215018
C	-1.470893	3.107411	-0.828105
C	-2.733076	-1.172745	0.996995
C	-3.178763	0.133649	0.773217
C	2.412767	-1.631240	-0.893832
C	2.806750	-0.399699	-1.426020
H	-0.418053	-4.458976	0.091312
H	0.397311	-3.003823	1.957764
H	1.162979	3.958726	-1.679445
H	3.027302	2.437237	-1.952973
H	-3.240584	2.995649	0.349667
H	-1.429554	4.189600	-0.726950
H	-3.173646	-1.721525	1.825561
H	-3.936615	0.541245	1.438481
H	3.190504	-2.288450	-0.512988
H	3.866447	-0.154457	-1.428353
Ge	-0.132100	-3.016073	0.519408

5C-2Ge$_{1,2}$,

C	-3.798731	-1.742457	0.752617
C	-5.036828	-0.388890	-1.487462
C	-3.190081	-1.677014	-0.477571
C	-3.835089	-0.995841	-1.593409
C	-1.802525	-1.563117	-0.652409
C	-2.766992	-0.495565	-2.450070

C	-1.544492	-0.842958	-1.855092
C	-2.948005	0.632845	-3.212854
C	-0.398582	-0.072766	-2.028178
C	-0.925599	-1.543459	0.427802
C	-2.911319	-1.897991	1.850403
C	-1.521880	-1.822233	1.691612
C	0.369382	-0.964435	0.144376
C	0.618957	-0.268093	-1.018512
C	-0.518730	0.977882	-2.983410
C	-1.754858	1.330613	-3.539377
H	-6.796139	-1.861213	0.425692
H	-5.845279	0.302752	1.246843
H	-3.298376	-1.982125	2.862878
H	-0.899571	-1.850332	2.583301
H	1.139535	-0.987218	0.912034
H	1.577355	0.234355	-1.128132
H	0.340412	1.610519	-3.194942
H	-1.792066	2.223767	-4.158191
H	-5.829561	0.856067	-4.086318
H	-5.074644	2.430121	-2.293117
Ge	-4.892550	1.028730	-2.887031
Ge	-5.590374	-0.919057	0.356917

5C-2Ge$_{1,3}$

C	3.143281	0.368620	-3.040160
C	6.011727	0.260604	-3.002908
C	3.806946	-0.233369	-2.000960
C	5.261417	-0.297275	-1.982952
C	3.350737	-0.191939	-0.666108
C	5.671629	-0.289078	-0.649043
C	4.478148	-0.220203	0.182324
C	7.294908	0.701501	-2.625170
C	6.863406	0.277534	-0.233175
C	7.711210	0.709699	-1.271473
C	4.510757	0.395536	1.408147
C	2.182084	0.484268	-0.311061
C	2.131534	0.934970	1.046787
C	3.258941	0.904643	1.865656
C	1.848847	0.876684	-2.721419
C	1.375971	0.919746	-1.411032
H	4.587039	-0.168753	-5.602050
H	4.666341	2.196364	-4.785247
H	7.956942	1.163247	-3.353728
H	8.666689	1.177122	-1.045929
H	6.746062	2.235678	1.978366
H	7.145399	-0.121230	2.721537
H	1.237162	1.433605	1.413326
H	3.186240	1.375142	2.843205
H	1.238053	1.336677	-3.494463
H	0.428406	1.417296	-1.217513
Ge	4.611655	0.725751	-4.358785
Ge	6.465308	0.761271	1.670317

5C-3Ge$_{1,2,3}$

C	4.361164	-0.888527	-1.113765
C	5.780936	-0.936738	-0.780035
C	3.939312	-0.280207	-2.268613
C	6.740926	-0.384773	-1.551835
C	3.620011	-0.875104	0.085673
C	4.498581	-0.936240	1.186755
C	5.868514	-0.967524	0.684808
C	6.914973	-0.446241	1.359383
C	4.218557	-0.377011	2.407594
C	2.374989	-0.239055	0.173112
C	1.985778	0.140436	1.495831
C	2.881863	0.089991	2.564110
C	2.594740	0.189581	-2.245586
C	1.832253	0.193580	-1.077186
H	6.004340	-0.596921	-4.440153
H	5.795549	1.677692	-3.408724
H	8.295422	1.625767	-0.140759
H	9.452237	-0.595967	-0.257019
H	6.203134	1.535083	3.399159
H	6.524845	-0.780466	4.303611
H	1.014377	0.602211	1.656606
H	2.558419	0.513866	3.511914
H	2.161857	0.651486	-3.129811
H	0.849443	0.658571	-1.102270
Ge	5.688516	0.169426	-3.151492
Ge	8.092578	0.104231	-0.160904
Ge	6.061543	0.038594	3.094432

5C-3Ge$_{1,2,4}$

C	4.781715	-0.901799	1.162670
C	3.538768	-1.082708	1.910557
C	5.963389	-0.640965	1.834142
C	3.484283	-1.022818	3.259989
C	4.466279	-0.325377	-0.068178
C	3.021261	-0.134975	-0.134761
C	2.457694	-0.595679	1.055492
C	1.327928	-0.030280	1.620515
C	2.483164	0.917442	-0.859242
C	5.321236	0.543310	-0.728636
C	6.910008	0.106155	1.109503
C	6.593607	0.684567	-0.143834
H	5.989913	-2.114173	4.463283

H	5.701082	0.372745	4.574651
H	0.684271	-1.422552	4.212945
H	1.589485	0.907714	4.390322
H	7.866718	0.369480	1.554060
H	7.330907	1.351890	-0.584271
C	1.243313	1.389952	-0.389862
C	0.676178	0.927734	0.822590
H	0.746637	2.220071	-0.886957
H	-0.216004	1.434683	1.182138
H	4.095185	1.301919	-3.345128
H	4.259657	3.141705	-1.650315
Ge	4.070017	1.634291	-1.849539
Ge	1.620907	-0.390960	3.575344
Ge	5.410883	-0.887609	3.750490

5C-4Ge

C	3.619160	-1.213370	-2.641710
C	5.021820	-0.814410	-2.692230
C	2.628950	-0.268960	-2.883260
C	5.419120	0.446200	-2.975510
C	3.462680	-2.251090	-1.725100
C	4.755760	-2.578860	-1.133750
C	5.739730	-1.667280	-1.734460
C	6.817900	-1.228790	-1.053990
C	4.890290	-3.060780	0.122010
C	2.303770	-2.425390	-0.978530
C	1.397640	-0.526760	-2.265140
C	1.237970	-1.585570	-1.329890
H	3.402380	2.547560	-2.606860
H	3.567750	1.698280	-4.956410
H	6.973230	1.671750	-0.841150
H	8.400150	0.740700	-2.678460
H	0.564950	0.163580	-2.379920
H	0.293850	-1.634240	-0.791960
H	2.583270	-4.828510	0.809410
H	2.627740	-2.591130	1.932530
H	6.514390	-1.362620	1.839330
H	7.790670	-3.299840	0.891990
Ge	3.707260	1.322410	-3.477250
Ge	7.093040	0.565360	-1.897840
Ge	6.661040	-2.297560	0.631270
Ge	3.000910	-3.361540	0.660250

5C-5Ge

C	0.321900	1.684910	0.801900
C	-0.944950	0.943020	0.790030
C	1.418500	0.711350	0.730110
C	-0.631330	-0.489050	0.710830
C	0.829360	-0.632240	0.673850
C	0.430940	2.929710	0.290270
C	-2.076770	1.461190	0.266820
C	2.601620	1.002610	0.148190
C	1.435470	-1.656980	0.036830
C	-1.455880	-1.373560	0.110050
Ge	-3.006860	-0.198680	-0.340230
Ge	-0.145380	-2.738970	-0.527780
Ge	3.160240	-0.803100	-0.496690
Ge	2.341800	2.933610	-0.290390
Ge	-1.469490	3.307230	-0.193780
H	-3.215360	-0.160290	-1.859930
H	-0.194220	-2.841810	-2.057970
H	3.295500	-0.798410	-2.025130
H	2.431130	3.146030	-1.807400
H	-1.591890	3.540280	-1.705450
H	-4.331970	-0.527360	0.355270
H	-0.266790	-4.136540	0.088430
H	4.429710	-1.386090	0.132790
H	3.267020	3.923050	0.425610
H	-2.148000	4.453920	0.562780

5C-Sn

C	-1.714034	-1.716297	0.303085
C	1.117875	-1.968570	-0.737210
C	-1.173085	-0.938145	-0.690888
C	0.194521	-1.060053	-1.193335
C	-1.589757	0.383620	-0.932127
C	0.558003	0.192254	-1.721197
C	-0.528009	1.079796	-1.551759
C	-0.368352	2.447928	-1.448779
C	-2.557304	1.021683	-0.167368
C	1.874808	0.626824	-1.795632
C	0.962942	2.922576	-1.736541
C	2.029358	2.057327	-1.902594
C	-2.518805	2.462500	-0.231203
C	-1.477523	3.139959	-0.839422
C	-2.824964	-1.140533	0.984763
C	-3.239297	0.175291	0.757810
C	2.478897	-1.612871	-0.963499
C	2.848894	-0.367085	-1.478785
H	-0.463850	-4.777528	0.044150
H	0.464196	-3.262854	2.202874
H	1.159034	3.992429	-1.733718
H	3.024922	2.478145	-2.023516
H	-3.277257	3.039388	0.293023
H	-1.455724	4.225340	-0.772058
H	-3.329931	-1.698045	1.769710

H	-4.032338	0.583188	1.380616
H	3.275378	-2.286301	-0.656793
H	3.907700	-0.124566	-1.536349
Sn	-0.119201	-3.201700	0.599639

5C-2Sn$_{1,2}$

C	-3.751215	-1.730669	0.867879
C	-5.110965	-0.308319	-1.455017
C	-3.187642	-1.486256	-0.360074
C	-3.848849	-0.788400	-1.472247
C	-1.805451	-1.388034	-0.555128
C	-2.763581	-0.300857	-2.335754
C	-1.549528	-0.672567	-1.747420
C	-2.870597	0.730839	-3.235404
C	-0.349261	-0.020789	-1.995851
C	-0.879024	-1.502032	0.472472
C	-2.820981	-1.997316	1.909658
C	-1.435736	-1.893968	1.723572
C	0.435285	-0.993842	0.142886
C	0.685214	-0.294920	-1.021540
C	-0.415664	0.957593	-3.028857
C	-1.634419	1.319573	-3.618782
H	-7.030287	-2.194516	0.467960
H	-6.245368	0.273915	1.538528
H	-3.175694	-2.221063	2.912704
H	-0.787607	-2.031777	2.586174
H	1.239798	-1.095567	0.867749
H	1.676936	0.126713	-1.169033
H	0.478593	1.507942	-3.312710
H	-1.618694	2.131325	-4.341827
H	-5.969866	0.756772	-4.465181
H	-5.367572	2.737316	-2.575477
Sn	-5.026594	1.143667	-3.094226
Sn	-5.797861	-1.011871	0.503351

5C-2Sn$_{1,3}$

C	3.057845	0.439981	-3.053515
C	6.086413	0.317684	-3.028325
C	3.811750	-0.029669	-2.008333
C	5.269156	-0.085252	-1.989066
C	3.367697	0.000231	-0.672501
C	5.680729	-0.077060	-0.650697
C	4.485519	-0.016168	0.183442
C	7.409004	0.630353	-2.659050
C	6.939335	0.334107	-0.254464
C	7.825077	0.638337	-1.306398
C	4.447196	0.467788	1.466170
C	2.139962	0.540529	-0.298410
C	2.041924	0.898120	1.083619
C	3.155059	0.867208	1.923497
C	1.730801	0.839172	-2.710409
C	1.281583	0.883369	-1.390668
H	4.553813	-0.371212	-5.890504
H	4.664762	2.339314	-5.190663
H	8.128195	0.959453	-3.405235
H	8.838235	0.972929	-1.096773
H	6.973484	2.383345	2.311595
H	7.278901	-0.320680	2.980859
H	1.107446	1.295661	1.473049
H	3.029211	1.233907	2.939332
H	1.054802	1.195213	-3.484016
H	0.288479	1.280051	-1.192270
Sn	4.601368	0.739362	-4.594461
Sn	6.589092	0.778038	1.870686

5C-3Sn$_{1,2,3}$

C	4.372569	-0.538214	-1.105643
C	5.799246	-0.558444	-0.781878
C	3.833835	-0.159216	-2.306942
C	6.803035	-0.235349	-1.623002
C	3.643272	-0.550506	0.090931
C	4.509661	-0.586057	1.191823
C	5.887691	-0.589373	0.700244
C	6.985205	-0.299245	1.428759
C	4.118471	-0.258543	2.463336
C	2.307270	-0.156224	0.178847
C	1.852095	0.066080	1.515803
C	2.727030	0.020649	2.604709
C	2.436160	0.122199	-2.270245
C	1.696972	0.120285	-1.084144
H	5.962155	-0.956013	-4.736720
H	5.858710	1.750360	-4.036478
H	9.032974	1.658908	-0.184342
H	9.724700	-1.047435	-0.282623
H	6.339599	1.582497	4.017510
H	6.518547	-1.150286	4.587793
H	0.817722	0.350584	1.694278
H	2.320580	0.267255	3.582709
H	1.916980	0.408193	-3.181922
H	0.649434	0.409413	-1.126437
Sn	5.683283	0.149034	-3.463414
Sn	8.424529	0.059302	-0.181633
Sn	6.093135	0.005885	3.403518

5C-3Sn$_{1,2,4}$

C	4.811938	-0.607750	1.291650

C	3.571156	-0.787672	2.053394
C	6.076027	-0.555076	1.844511
C	3.487414	-0.954238	3.392208
C	4.496382	-0.036330	0.058365
C	3.055697	0.153177	-0.008113
C	2.493115	-0.302706	1.184648
C	1.240712	0.080971	1.621333
C	2.411189	1.030128	-0.862106
C	5.419955	0.634319	-0.723263
C	7.058878	0.029792	1.023733
C	6.737285	0.611220	-0.227192
H	6.162727	-2.519093	4.536465
H	6.036571	0.242561	5.018735
H	0.407359	-1.764487	4.268182
H	1.194711	0.876863	4.796624
H	8.086291	0.119719	1.368175
H	7.539041	1.111364	-0.765678
C	1.093246	1.353727	-0.487613
C	0.520582	0.889880	0.722007
H	0.499943	2.037509	-1.090406
H	-0.475617	1.246032	0.973093
H	4.095525	1.177513	-3.707164
H	4.315006	3.430136	-2.046952
Sn	4.094395	1.735885	-2.092577
Sn	1.386595	-0.462990	3.750572
Sn	5.602359	-1.016692	3.945337

5C-4Sn

C	3.541298	-0.867502	-2.259244
C	4.934176	-0.443353	-2.296311
C	2.480684	-0.165080	-2.804215
C	5.410493	0.675808	-2.880780
C	3.385722	-1.901465	-1.347082
C	4.666954	-2.217393	-0.731110
C	5.656530	-1.295681	-1.328184
C	6.906559	-1.084535	-0.875402
C	4.858005	-2.993366	0.356253
C	2.153964	-2.335701	-0.889345
C	1.214961	-0.628378	-2.411619
C	1.055426	-1.688261	-1.476600
H	3.154976	3.030516	-3.127157
H	3.533854	1.528960	-5.457518
H	7.750109	2.065552	-1.067054
H	8.698185	0.479450	-3.168122
H	0.309798	-0.147977	-2.776051
H	0.039003	-1.947082	-1.188899
H	2.503023	-5.296609	0.571921
H	2.232246	-3.110251	2.298105
H	7.167507	-1.756385	2.310790
H	8.055515	-3.798582	0.616604
Sn	3.581639	1.497137	-3.749969
Sn	7.429949	0.697657	-2.042429
Sn	6.945339	-2.510156	0.791740
Sn	2.809512	-3.628191	0.775052

5C-5Sn

C	0.293621	1.735328	-0.175817
C	-0.974691	0.991554	-0.189140
C	1.392621	0.760985	-0.245667
C	-0.659602	-0.442528	-0.267182
C	0.803496	-0.585018	-0.302387
C	0.425653	3.075230	-0.107592
C	-2.209585	1.529619	-0.135244
C	2.709193	1.050673	-0.252790
C	1.485031	-1.746095	-0.371152
C	-1.554819	-1.450049	-0.297947
Sn	-3.412429	-0.297378	-0.201456
Sn	-0.188502	-3.155966	-0.406256
Sn	3.532589	-0.973849	-0.368681
Sn	2.608358	3.233528	-0.137746
Sn	-1.684381	3.651616	-0.035699
H	-4.401173	-0.436002	-1.589199
H	-0.310058	-4.066930	-1.847753
H	4.407305	-1.297553	-1.801402
H	3.236896	4.039763	-1.507966
H	-2.207978	4.571274	-1.378324
H	-4.341915	-0.581564	1.204924
H	-0.256771	-4.197665	0.947588
H	4.456677	-1.434701	0.993829
H	3.284592	3.894508	1.286532
H	-2.152231	4.424218	1.415824

5C-N

N	-0.104408	2.218578	-1.869563
C	0.968913	1.302854	-1.981211
C	-1.302149	1.485709	-1.692114
C	0.400486	0.053058	-2.145207
C	-0.996423	0.165535	-1.967594
C	0.858432	-1.075863	-1.438112
C	-1.436181	-0.891410	-1.146738
C	-0.289588	-1.695237	-0.847766
C	-0.192230	-2.384234	0.355491
C	2.086032	-1.038942	-0.781564
C	-2.440648	-0.675120	-0.206628

C	-1.387606	-2.429039	1.155693
C	-2.458351	-1.595783	0.899060
C	2.232734	-1.972402	0.303765
C	1.141987	-2.632122	0.834719
C	2.304518	1.337170	-1.484569
C	2.850144	0.168690	-0.954759
C	-2.449176	1.719669	-0.879220
C	-3.023558	0.640990	-0.208218
H	0.003296	3.192228	-1.639597
H	-1.388304	-3.012998	2.073134
H	-3.264867	-1.543716	1.627121
H	3.192847	-2.062075	0.807136
H	1.278611	-3.227327	1.734546
H	2.860741	2.265456	-1.386799
H	3.832695	0.237221	-0.493356
H	-2.810416	2.721688	-0.664319
H	-3.837530	0.854024	0.481068

5C-2N$_{1,2}$

N	2.930697	0.662181	0.065315
C	2.347524	0.057477	1.215199
C	2.632450	-0.140780	-1.067835
C	1.820748	-1.162337	0.805437
C	2.077939	-1.302925	-0.581944
C	0.439654	-1.428116	1.034132
C	0.847480	-1.553881	-1.240773
C	-0.172755	-1.675023	-0.252817
C	0.700561	-0.604438	-2.245451
C	-1.459933	-1.204771	-0.523861
C	-0.249662	-0.716940	2.019460
C	1.772019	0.672580	2.360337
C	0.522550	0.223489	2.781975
C	-1.677043	-0.611860	1.845494
C	-2.259710	-0.846900	0.621370
C	-1.674791	-0.661374	-1.835802
C	-0.620305	-0.290166	-2.667057
N	1.853754	0.229954	-2.196789
H	2.183747	1.573729	2.806310
H	0.033401	0.774841	3.581803
H	-2.279846	-0.176350	2.639081
H	-3.307042	-0.590026	0.480178
H	-2.671002	-0.315215	-2.101735
H	-0.822211	0.363376	-3.511193
H	3.522414	1.477212	0.080938
H	2.104163	0.907761	-2.898574

5C-2N$_{1,3}$

N	6.917688	-0.047180	-1.323430
C	5.908698	0.808410	-1.811920
C	6.337937	-1.336403	-1.132461
C	4.857880	0.006855	-2.234836
C	5.149652	-1.337697	-1.846120
C	3.515682	0.318986	-1.871795
C	3.975684	-1.889593	-1.301124
C	2.958889	-0.886057	-1.352999
C	6.405295	-2.166153	0.022434
C	3.970245	-2.449890	-0.033252
C	5.233423	-2.716993	0.566596
C	2.295702	-0.890596	-0.134056
N	2.775903	-1.994510	0.599988
C	3.221084	1.545029	-1.264723
C	2.185485	1.521216	-0.264778
C	1.748496	0.342149	0.327365
C	5.614797	2.160265	-1.468603
C	4.288871	2.510499	-1.242000
H	7.312565	-2.232308	0.617672
H	5.305384	-3.175508	1.549605
H	1.874062	2.458628	0.190946
H	1.135750	0.398464	1.222853
H	6.396888	2.872939	-1.221846
H	4.092274	3.502181	-0.840319
H	7.660921	0.294501	-0.736067
H	2.639697	-2.067193	1.595109

5C-3N$_{1,2,3}$

C	5.461531	-1.003557	-1.090841
C	4.979156	-1.562961	0.132147
C	6.201261	0.134198	-0.759648
N	6.135541	0.279419	0.637129
C	5.235676	-0.689107	1.163104
C	4.349137	-0.823464	-1.991017
C	3.177467	-1.302658	-1.302642
C	3.566237	-1.754566	-0.000643
C	2.931374	-0.972250	0.930878
C	2.127211	-0.399305	-1.133942
C	4.272380	0.337924	-2.772113
N	3.946468	-0.332090	1.738983
N	1.890045	-0.291303	0.256102
C	2.958062	0.890791	-2.979352
C	1.897672	0.581880	-2.142222
C	6.367008	1.152939	-1.746052
C	5.428163	1.201051	-2.762818
H	6.583588	1.017195	1.155123
H	3.830505	-0.218822	2.737891
H	1.304666	0.419457	0.661801
H	2.842759	1.739261	-3.649812

H	1.008038	1.204765	-2.157830
H	7.066302	1.975342	-1.626903
H	5.448883	2.060321	-3.429533

5C-3N$_{1,2,3}$

C	5.461531	-1.003557	-1.090841
C	4.979156	-1.562961	0.132147
C	6.201261	0.134198	-0.759648
N	6.135541	0.279419	0.637129
C	5.235676	-0.689107	1.163104
C	4.349137	-0.823464	-1.991017
C	3.177467	-1.302658	-1.302642
C	3.566237	-1.754566	-0.000643
C	2.931374	-0.972250	0.930878
C	2.127211	-0.399305	-1.133942
C	4.272380	0.337924	-2.772113
N	3.946468	-0.332090	1.738983
N	1.890045	-0.291303	0.256102
C	2.958062	0.890791	-2.979352
C	1.897672	0.581880	-2.142222
C	6.367008	1.152939	-1.746052
C	5.428163	1.201051	-2.762818
H	6.583588	1.017195	1.155123
H	3.830505	-0.218822	2.737891
H	1.304666	0.419457	0.661801
H	2.842759	1.739261	-3.649812
H	1.008038	1.204765	-2.157830
H	7.066302	1.975342	-1.626903
H	5.448883	2.060321	-3.429533

5C-P

C	-1.556782	1.953947	-0.495858
C	1.133959	2.185870	-0.657258
C	-0.855921	0.885653	-0.997267
C	0.571335	1.008665	-1.083032
C	-1.164392	-0.455779	-0.711643
C	1.135866	-0.257426	-0.849797
C	0.065217	-1.166510	-0.620233
C	0.209863	-2.270968	0.201234
C	-2.276840	-0.793327	0.051466
C	2.372404	-0.392327	-0.228052
C	1.560599	-2.560211	0.614219
C	2.592374	-1.663426	0.415593
C	-2.198704	-2.076586	0.703714
C	-1.012897	-2.782140	0.769067
C	-2.789895	1.624209	0.138490
C	-3.151192	0.294889	0.367917
C	2.471953	2.078040	-0.177737
C	3.079569	0.832374	-0.006724
H	-0.431397	4.070311	-1.391311
H	1.753838	-3.452621	1.205158
H	3.561329	-1.876621	0.861018
H	-3.057175	-2.447276	1.259020
H	-0.979257	-3.688168	1.369872
H	-3.424254	2.404405	0.551316
H	-4.056678	0.102037	0.938674
H	3.008376	2.959260	0.164672
H	4.066231	0.802651	0.449917
P	-0.296613	3.330403	-0.185911

5C-2P$_{1,2}$

C	4.712889	0.245730	0.420698
C	3.495865	2.598367	-0.242328
C	3.659468	0.193869	-0.462578
C	3.045951	1.446048	-0.804451
C	2.643741	-0.784082	-0.402660
C	1.635247	1.217500	-0.940938
C	1.392900	-0.151681	-0.698230
C	0.803831	2.222012	-0.502389
C	0.204596	-0.572221	-0.108578
C	2.731227	-1.849624	0.488248
C	4.908508	-0.926507	1.204115
C	3.967157	-1.957392	1.198881
C	1.470297	-2.462598	0.839078
C	0.267699	-1.854583	0.555051
C	-0.783666	0.444314	0.076570
C	-0.485040	1.799998	-0.069757
H	6.041615	2.437281	0.043008
H	5.730359	-0.988462	1.912881
H	4.110183	-2.784899	1.890020
H	1.469316	-3.359635	1.453947
H	-0.644986	-2.290842	0.954327
H	-1.746141	0.175744	0.505985
H	-1.219853	2.524951	0.270924
H	1.930966	4.518039	-0.921357
P	4.928383	2.073505	0.847379
P	1.946620	3.579624	0.145406

5C-2P$_{1,3}$

C	3.233344	0.305019	-3.020971
C	5.924112	0.214169	-2.968299
C	3.806372	-0.406415	-1.996483
C	5.249552	-0.476690	-1.979247
C	3.337930	-0.348609	-0.661269
C	5.660365	-0.468883	-0.643382
C	4.475733	-0.393624	0.180892

C	7.162829	0.772476	-2.583099
C	6.771642	0.230825	-0.211928
C	7.578004	0.780652	-1.232685
C	4.574600	0.331293	1.342212
C	2.224194	0.424402	-0.323672
C	2.220698	0.948938	1.010527
C	3.365570	0.933653	1.801870
C	1.972616	0.905947	-2.728907
C	1.469894	0.933672	-1.431285
H	4.599666	-0.234057	-5.099405
H	7.749713	1.352471	-3.290790
H	8.459123	1.366424	-0.983409
H	6.874050	-0.191956	2.298995
H	1.363169	1.521198	1.356524
H	3.353110	1.498359	2.730723
H	1.438361	1.460225	-3.496463
H	0.563865	1.504612	-1.242438
P	4.621534	0.852278	-4.183002
P	6.374386	0.885694	1.518362

5C-3P$_{1,2,3}$

C	3.333653	-0.120643	0.099274
C	4.767620	-0.260349	0.200114
C	2.761584	0.835112	0.904083
C	5.469562	0.584116	1.006895
C	2.954968	-0.308393	-1.256984
C	4.130461	-0.568130	-2.011016
C	5.265425	-0.539973	-1.118441
C	6.425773	0.047127	-1.525818
C	4.336235	-0.049466	-3.266698
C	1.899119	0.424547	-1.811110
C	1.992353	0.671960	-3.220105
C	3.181455	0.481078	-3.915499
C	1.543334	1.401801	0.423756
C	1.107643	1.169351	-0.876276
H	4.075075	0.940680	3.119125
H	8.026429	0.528040	0.480313
H	6.757047	-0.567336	-3.984138
H	1.179800	1.190383	-3.723503
H	3.247742	0.867918	-4.929361
H	1.001550	2.130922	1.021127
H	0.240167	1.719009	-1.233867
P	6.136495	0.522634	-3.317461
P	6.876104	1.139171	-0.083380
P	4.174695	1.625108	1.878551

5C-3P$_{1,2,4}$

C	1.596832	0.372020	-1.044439
C	2.787332	-0.183625	-0.439898
C	0.576004	0.710167	-0.171637
C	2.829053	-0.356591	0.907709
C	2.005653	1.224798	-2.080850
C	3.458177	1.229891	-2.125096
C	3.935027	0.380167	-1.115663
C	5.004666	0.725125	-0.306441
C	4.059557	2.444090	-2.408503
C	1.380082	2.434759	-2.327135
C	-0.198812	1.832374	-0.536376
C	0.196824	2.675137	-1.595893
H	0.499997	-1.079949	1.686539
H	5.206556	-1.062806	1.544507
H	-1.008167	2.176975	0.102285
H	-0.340661	3.612576	-1.715726
C	5.283288	2.692458	-1.750372
C	5.748426	1.852476	-0.717206
H	5.806029	3.633572	-1.902376
H	6.592896	2.202718	-0.129089
H	2.657641	3.378954	-4.303800
P	2.699075	3.661257	-2.910478
P	4.538575	0.187601	1.445943
P	1.151420	0.175503	1.549066

5C-4P

C	3.656887	-1.379549	-2.832345
C	5.060723	-1.016545	-2.888325
C	2.763368	-0.319970	-2.897683
C	5.400286	0.300228	-2.971601
C	3.498903	-2.425930	-1.907232
C	4.798273	-2.755354	-1.351892
C	5.763752	-1.857082	-1.945264
C	6.698257	-1.282425	-1.135185
C	4.897018	-3.037623	-0.022828
C	2.441042	-2.454679	-1.009709
C	1.568982	-0.470738	-2.165911
C	1.410334	-1.521527	-1.236721
H	3.735578	1.353489	-4.586165
H	7.912837	0.656936	-2.361943
H	0.830440	0.326984	-2.147706
H	0.559669	-1.466547	-0.561785
H	2.857841	-4.453217	0.549867
H	7.377945	-2.905845	0.771581
P	6.694010	0.516927	-1.647584
P	3.792266	1.247368	-3.169901
P	6.320336	-1.983095	0.558445
P	3.142229	-3.063618	0.641663

5C-5P

C	-1.219481	-1.264043	0.738424
C	-1.573363	0.121698	0.973475
C	0.195486	-1.417741	1.012662
C	-0.377238	0.824504	1.393184
C	0.716134	-0.126814	1.417199
C	-1.740938	-1.891082	-0.355845
C	0.942295	-2.182179	0.163973
C	1.929428	0.265752	0.931088
C	-0.143834	2.069569	0.885583
C	-2.412071	0.736782	0.089865
P	-2.803420	-0.586571	-1.172768
P	-0.282192	-2.780721	-1.117037
P	2.476115	-1.158982	-0.150343
P	1.660363	2.036815	0.393157
P	-1.603309	2.390861	-0.239129
H	-4.125627	-0.981242	-0.837123
H	-0.498783	-4.137190	-0.756930
H	3.469707	-1.804926	0.632080
H	2.294228	2.791477	1.415586
H	-2.399301	3.300433	0.506297

5C-As

C	-1.618034	-1.741873	0.239638
C	1.001145	-1.975445	-0.722516
C	-1.217168	-0.988053	-0.831771
C	0.129174	-1.107945	-1.326143
C	-1.643346	0.334624	-1.048861
C	0.515356	0.142328	-1.841612
C	-0.575426	1.035744	-1.669971
C	-0.390420	2.395524	-1.496199
C	-2.548711	0.959365	-0.198572
C	1.838328	0.568497	-1.810265
C	0.952332	2.867378	-1.728033
C	2.016333	1.998100	-1.874036
C	-2.500291	2.400479	-0.215002
C	-1.473021	3.083361	-0.837122
C	-2.666694	-1.184413	1.023265
C	-3.136126	0.111596	0.791281
C	2.378107	-1.634074	-0.830503
C	2.782936	-0.415965	-1.383723
H	-0.500763	-4.125403	-0.215869
H	1.156401	3.934309	-1.673803
H	3.021819	2.409258	-1.925745
H	-3.223541	2.965864	0.368111
H	-1.424976	4.164209	-0.725743
H	-3.058309	-1.722107	1.882903
H	-3.873487	0.521783	1.477581
H	3.140811	-2.274593	-0.395181
H	3.841196	-0.166152	-1.357127
As	-0.072078	-2.967062	0.671226

5C-2As$_{1,2}$

C	-3.816064	-1.711552	0.705444
C	-5.009545	-0.414017	-1.496827
C	-3.205865	-1.735006	-0.524125
C	-3.836667	-1.068603	-1.637500
C	-1.811528	-1.631562	-0.694016
C	-2.785324	-0.560152	-2.484726
C	-1.553042	-0.909686	-1.898787
C	-2.984361	0.611701	-3.171309
C	-0.423417	-0.111311	-2.050960
C	-0.948566	-1.577911	0.396346
C	-2.947385	-1.828735	1.822805
C	-1.558159	-1.799342	1.667219
C	0.343337	-0.989226	0.123539
C	0.592741	-0.292768	-1.038624
C	-0.564604	0.976491	-2.963507
C	-1.809988	1.348939	-3.478610
H	-6.470788	-1.871741	0.200274
H	-3.345246	-1.837292	2.834388
H	-0.941757	-1.797467	2.563339
H	1.103877	-0.993762	0.900914
H	1.541670	0.228944	-1.139377
H	0.284687	1.629102	-3.152485
H	-1.871697	2.280531	-4.035430
H	-5.589573	0.587893	-3.905551
As	-4.864490	1.179686	-2.706866
As	-5.534953	-0.693139	0.419106

5C-2As$_{1,3}$

C	3.188362	0.339460	-3.028967
C	5.974298	0.250921	-2.982977
C	3.814703	-0.316406	-2.002044
C	5.259091	-0.377984	-1.983905
C	3.351424	-0.267122	-0.666337
C	5.670292	-0.370488	-0.646756
C	4.485198	-0.304365	0.179082
C	7.228238	0.768636	-2.604440
C	6.821180	0.266943	-0.228926
C	7.643975	0.776544	-1.252205
C	4.541480	0.364265	1.373593
C	2.207880	0.455236	-0.319067
C	2.178997	0.944748	1.026734
C	3.314603	0.924575	1.832982
C	1.913790	0.899342	-2.726004

C	1.426924	0.931269	-1.421286
H	4.590629	-0.411344	-5.161606
H	7.848697	1.298650	-3.322710
H	8.558973	1.312272	-1.012292
H	6.904696	-0.362111	2.361145
H	1.301891	1.479668	1.383581
H	3.271975	1.444031	2.786999
H	1.341440	1.409706	-3.496554
H	0.499403	1.465779	-1.229500
As	4.621929	0.871468	-4.344968
As	6.464370	0.908377	1.649798

5C-3As$_{1,2,3}$

C	4.383505	-0.992169	-1.115518
C	5.788613	-1.046254	-0.772793
C	4.003278	-0.300180	-2.235947
C	6.722281	-0.407618	-1.515267
C	3.628249	-0.983015	0.082945
C	4.520729	-1.040175	1.181462
C	5.874971	-1.076371	0.671936
C	6.891547	-0.466421	1.323781
C	4.277777	-0.396517	2.366999
C	2.413121	-0.293912	0.169712
C	2.048909	0.138773	1.484940
C	2.960920	0.118710	2.537101
C	2.676968	0.218652	-2.226763
C	1.896544	0.192428	-1.073858
H	5.951881	-0.734095	-4.050320
H	9.110967	-0.588443	-0.235060
H	6.428532	-0.900495	3.916615
H	1.095800	0.639746	1.637261
H	2.678111	0.609425	3.465147
H	2.287109	0.747086	-3.093268
H	0.933071	0.696514	-1.090861
As	5.714148	0.388041	-3.052182
As	7.946742	0.385254	-0.146465
As	6.075358	0.261668	3.001907

5C-3As$_{1,2,4}$

C	4.766747	-0.991918	1.134366
C	3.527945	-1.183413	1.862466
C	5.901374	-0.642571	1.841371
C	3.483392	-1.050904	3.206877
C	4.451876	-0.419653	-0.105720
C	3.014713	-0.230248	-0.171965
C	2.451878	-0.687056	1.027605
C	1.386996	-0.048003	1.633239
C	2.520461	0.872228	-0.843947
C	5.272753	0.509618	-0.717039
C	6.827424	0.169616	1.160142
C	6.516723	0.733835	-0.098152
H	5.764453	-2.102687	4.108273
H	0.935398	-1.464359	3.887472
H	7.737370	0.506406	1.650371
H	7.211592	1.464757	-0.504398
C	1.326009	1.417500	-0.337344
C	0.767818	0.967761	0.880882
H	0.883136	2.298119	-0.796160
H	-0.064628	1.534067	1.290835
H	4.025937	0.883922	-3.035633
As	4.081198	1.741468	-1.779518
As	5.362354	-0.671496	3.786304
As	1.722403	-0.190854	3.618193

5C-4As

C	3.650253	-1.287524	-2.726994
C	5.050403	-0.908379	-2.774935
C	2.699312	-0.292026	-2.879001
C	5.420659	0.378939	-2.971348
C	3.492608	-2.331668	-1.803919
C	4.786798	-2.654123	-1.231964
C	5.756266	-1.748120	-1.823048
C	6.776387	-1.250317	-1.085600
C	4.903403	-3.048386	0.057832
C	2.376523	-2.429486	-0.989524
C	1.484223	-0.488005	-2.202247
C	1.325056	-1.541756	-1.270996
H	3.706532	1.304255	-4.795941
H	8.108313	0.560214	-2.596891
H	0.695867	0.259193	-2.251307
H	0.424089	-1.539348	-0.662269
H	2.807872	-4.650330	0.465217
H	7.547814	-3.153845	0.678713
As	6.908006	0.659370	-1.669085
As	3.725515	1.399207	-3.277719
As	6.500105	-2.053244	0.726802
As	3.034970	-3.176435	0.765997

5C-5As

C	-1.184779	-1.232603	0.581274
C	-1.540543	0.156179	0.820165
C	0.234548	-1.386091	0.854295
C	-0.341213	0.861166	1.240745
C	0.755923	-0.091808	1.261577
C	-1.776905	-1.936222	-0.414065
C	0.991904	-2.235282	0.118366

C	2.008830	0.289142	0.912986
C	-0.131422	2.148485	0.873056
C	-2.471309	0.772992	0.052223
As	-3.031184	-0.628601	-1.258932
As	-0.288985	-3.011664	-1.206066
As	2.708938	-1.248505	-0.155691
As	1.819384	2.222912	0.441860
As	-1.729242	2.606828	-0.238315
H	-4.339151	-1.073650	-0.623975
H	-0.560042	-4.356101	-0.549554
H	3.571524	-1.926651	0.896934
H	2.346049	2.856198	1.719968
H	-2.544327	3.382677	0.784250

5C-Sb

C	-1.669803	-1.733485	0.325210
C	1.096122	-1.980126	-0.691268
C	-1.174277	-0.976144	-0.704963
C	0.180393	-1.096865	-1.202579
C	-1.602012	0.342183	-0.951870
C	0.548006	0.150668	-1.741580
C	-0.542850	1.037793	-1.581905
C	-0.380462	2.405514	-1.471420
C	-2.551042	0.981850	-0.165075
C	1.865343	0.588385	-1.787397
C	0.952536	2.882238	-1.747330
C	2.021594	2.018431	-1.897531
C	-2.516019	2.422671	-0.230495
C	-1.483129	3.099188	-0.852336
C	-2.758895	-1.159664	1.039824
C	-3.197306	0.145044	0.794643
C	2.461620	-1.624857	-0.878387
C	2.836261	-0.392588	-1.421981
H	-0.576402	-4.340479	-0.369107
H	1.146642	3.952410	-1.740272
H	3.019158	2.438935	-2.001752
H	-3.265454	2.998676	0.307458
H	-1.457193	4.184348	-0.783254
H	-3.223049	-1.703719	1.858603
H	-3.973575	0.558224	1.434800
H	3.251378	-2.280686	-0.520536
H	3.894512	-0.143351	-1.455602
Sb	-0.050547	-3.185259	0.782314

5C-2Sb$_{1,2}$

C	-3.804325	-1.692569	0.828354
C	-5.111166	-0.290988	-1.444138
C	-3.237719	-1.544892	-0.412512
C	-3.883159	-0.847691	-1.514737
C	-1.849528	-1.465912	-0.608259
C	-2.816021	-0.366557	-2.379067
C	-1.592849	-0.749250	-1.805065
C	-2.939388	0.723649	-3.203126
C	-0.409063	-0.056080	-2.023919
C	-0.937083	-1.529504	0.437394
C	-2.890187	-1.912801	1.893382
C	-1.504247	-1.851581	1.704298
C	0.373854	-1.005848	0.121285
C	0.623615	-0.309064	-1.042764
C	-0.492889	0.970189	-3.008974
C	-1.719184	1.355976	-3.563629
H	-6.606719	-2.216466	0.139650
H	-3.254627	-2.055705	2.907654
H	-0.860476	-1.945956	2.575927
H	1.167260	-1.081606	0.861352
H	1.605066	0.139411	-1.179076
H	0.392606	1.548501	-3.262946
H	-1.723626	2.217490	-4.226768
H	-5.677981	0.407433	-4.213357
Sb	-5.049656	1.350020	-2.926095
Sb	-5.809212	-0.770186	0.599810

5C-2Sb$_{1,3}$

C	3.112194	0.416329	-3.046595
C	6.061387	0.324418	-3.014408
C	3.829829	-0.121744	-2.010883
C	5.277219	-0.170208	-1.991567
C	3.377149	-0.087377	-0.674398
C	5.689359	-0.161442	-0.651559
C	4.502574	-0.107363	0.177120
C	7.357099	0.729679	-2.643719
C	6.910884	0.341971	-0.251770
C	7.773247	0.738029	-1.291016
C	4.488835	0.445650	1.430535
C	2.175500	0.516606	-0.308900
C	2.098711	0.925935	1.060354
C	3.219296	0.904935	1.889204
C	1.802542	0.874574	-2.718278
C	1.341133	0.909459	-1.403380
H	4.574642	-0.651077	-5.322865
H	8.041995	1.141785	-3.380850
H	8.752700	1.155764	-1.070864
H	6.975197	-0.610743	2.498294
H	1.181497	1.371663	1.438483
H	3.120827	1.330425	2.884907
H	1.159989	1.288410	-3.491609

H	0.368159	1.353760	-1.206217
Sb	4.621748	0.903088	-4.601178
Sb	6.610723	0.939545	1.864528

5C-3Sb$_{1,2,3}$

C	4.428072	-0.707730	-1.112504
C	5.841728	-0.725955	-0.777986
C	3.935366	-0.199643	-2.285052
C	6.822440	-0.272996	-1.589186
C	3.686275	-0.718844	0.084653
C	4.565187	-0.756277	1.184586
C	5.929046	-0.756857	0.684079
C	7.000415	-0.336079	1.391341
C	4.216400	-0.298890	2.427617
C	2.391317	-0.202905	0.172824
C	1.965208	0.110204	1.501673
C	2.850959	0.079172	2.579242
C	2.562714	0.181448	-2.257097
C	1.811325	0.164943	-1.081635
H	5.932342	-1.104277	-4.228350
H	9.319030	-1.054764	-0.259036
H	6.429084	-1.275265	4.082795
H	0.958045	0.484437	1.670043
H	2.485523	0.428646	3.541892
H	2.086423	0.570488	-3.153874
H	0.792328	0.543623	-1.113243
Sb	5.758595	0.389983	-3.404910
Sb	8.380050	0.378294	-0.172101
Sb	6.161261	0.249876	3.345735

5C-3Sb$_{1,2,4}$

C	4.792309	-0.740052	1.262775
C	3.555908	-0.919438	2.009683
C	6.018910	-0.564173	1.870691
C	3.487266	-0.969412	3.357758
C	4.476795	-0.174462	0.021672
C	3.042652	0.014365	-0.044512
C	2.480731	-0.435609	1.156099
C	1.291362	0.058306	1.652495
C	2.448070	0.983738	-0.829135
C	5.370038	0.599040	-0.694287
C	6.983545	0.116744	1.105642
C	6.663967	0.687347	-0.149436
H	5.862092	-2.486582	4.077142
H	0.745017	-1.809618	3.846003
H	7.975629	0.310540	1.506156
H	7.431881	1.282622	-0.637918
C	1.176890	1.409551	-0.402796
C	0.608551	0.955960	0.811316
H	0.636029	2.176670	-0.952028
H	-0.332065	1.403874	1.122418
H	4.011970	0.695310	-3.259150
Sb	4.108404	1.866137	-2.009609
Sb	5.581638	-0.795180	4.033938
Sb	1.457732	-0.249542	3.843600

5C-4Sb

C	3.625502	-1.051136	-2.459260
C	5.018998	-0.648983	-2.497401
C	2.590109	-0.207220	-2.817052
C	5.455877	0.556028	-2.925221
C	3.468415	-2.091225	-1.539883
C	4.753650	-2.405054	-0.944335
C	5.731267	-1.491834	-1.536832
C	6.905591	-1.158413	-0.959250
C	4.914135	-3.026440	0.244755
C	2.264834	-2.360202	-0.914361
C	1.333758	-0.534107	-2.281930
C	1.174185	-1.590079	-1.349059
H	3.690036	1.144282	-5.166485
H	8.384468	0.307928	-3.021619
H	0.461730	0.079392	-2.495916
H	0.189747	-1.720712	-0.905816
H	2.768089	-4.993158	0.252393
H	7.793802	-3.587212	0.417081
Sb	7.319569	0.841896	-1.788290
Sb	3.624175	1.627235	-3.522140
Sb	6.852484	-2.257508	0.951443
Sb	2.860554	-3.425291	0.940951

5C-5Sb

C	-1.094749	-1.138519	0.151750
C	-1.451937	0.255408	0.391681
C	0.329871	-1.292425	0.425587
C	-0.248237	0.963016	0.814067
C	0.853067	0.006512	0.834842
C	-1.824151	-1.994708	-0.598047
C	1.090348	-2.309435	-0.037802
C	2.160522	0.347890	0.799148
C	-0.092412	2.304599	0.757040
C	-2.554938	0.856880	-0.107221
Sb	-3.476621	-0.732048	-1.321201
Sb	-0.327276	-3.465732	-1.263022
Sb	3.114487	-1.442969	-0.055532

Sb	2.092649	2.541992	0.630365
Sb	-1.979750	2.981579	-0.152309
H	-4.618109	-1.227995	-0.141005
H	-0.684116	-4.646218	-0.070880
H	3.617788	-2.116183	1.439571
H	2.342289	2.863752	2.296493
H	-2.750725	3.414005	1.317574

5C-O

O	-0.198771	3.345261	-0.295127
C	-1.252431	2.526077	0.168868
C	0.979767	2.720070	0.170500
C	-0.760636	1.628135	1.078342
C	0.648857	1.750648	1.079501
C	-1.100925	0.262730	1.040720
C	1.219593	0.464231	1.042475
C	0.131789	-0.470758	1.058502
C	0.238650	-1.696117	0.411404
C	-2.184338	-0.152938	0.267081
C	2.359492	0.241510	0.270184
C	1.552382	-2.054639	-0.055453
C	2.565207	-1.122181	-0.143609
C	-2.151432	-1.531860	-0.146366
C	-0.993233	-2.275787	-0.056623
C	-2.447361	2.196501	-0.514021
C	-2.923535	0.888184	-0.393237
C	2.214241	2.600989	-0.511153
C	2.908843	1.394494	-0.389387
H	1.705221	-3.035272	-0.500062
H	3.480501	-1.391975	-0.665538
H	-3.005992	-1.955674	-0.668905
H	-0.974616	-3.268473	-0.500415
H	-2.916419	2.874168	-1.220351
H	-3.790500	0.614414	-0.990257
H	2.560139	3.349184	-1.217323
H	3.811007	1.274677	-0.985048

5C-2$O_{1,2}$

O	0.806058	3.040663	-0.492927
C	-0.431860	2.624527	0.121875
C	1.763234	2.305979	0.179328
C	-0.143743	1.824021	1.210517
C	1.271993	1.722940	1.296166
C	-0.694690	0.508951	1.242851
C	1.618533	0.346565	1.214188
C	0.419511	-0.425222	1.243960
C	2.461513	0.200969	0.128841
C	0.344023	-1.596216	0.480133
C	-1.834205	0.228566	0.478893
C	-1.646930	2.464113	-0.564407
C	-2.378212	1.304282	-0.291344
C	-2.035397	-1.157057	0.119436
C	-0.984399	-2.037140	0.119030
C	1.500649	-1.945832	-0.285614
C	2.517629	-1.024719	-0.555150
O	2.657303	1.491262	-0.487340
H	-1.936946	3.101306	-1.393524
H	-3.266706	1.119665	-0.890460
H	-2.979953	-1.460836	-0.325478
H	-1.117967	-3.020769	-0.324837
H	1.476067	-2.854855	-0.881876
H	3.199195	-1.201660	-1.380762

5C-2$O_{1,3}$

O	4.313102	-0.238278	2.187703
C	3.181908	-0.244509	1.357721
C	5.225863	-1.131232	1.564625
C	3.253428	-1.292451	0.471193
C	4.543981	-1.893084	0.643756
C	3.003471	-1.081155	-0.918038
C	5.083095	-2.113742	-0.636644
C	4.123832	-1.649753	-1.595779
C	6.577002	-0.798056	1.310850
C	6.299338	-1.571950	-0.983712
C	7.116713	-1.019479	0.029850
C	4.818772	-0.916811	-2.528082
O	6.196891	-1.011845	-2.286012
C	2.492480	0.151087	-1.346310
C	3.002052	0.652384	-2.596645
C	4.177911	0.175801	-3.164811
C	2.416639	0.898835	1.016276
C	2.039987	1.047259	-0.313957
H	7.137746	-0.175879	2.001521
H	8.069828	-0.558731	-0.210494
H	2.588453	1.574830	-2.997683
H	4.660858	0.740847	-3.955408
H	2.273628	1.720184	1.710892
H	1.578763	1.988946	-0.602743

5C-3$O_{1,2,4}$

C	2.411785	-0.752153	-1.343357
C	3.485255	-1.267606	-0.533147
C	1.626466	0.047753	-0.530943
O	2.143120	-0.023826	0.824933
C	3.354881	-0.652874	0.667207
C	3.000933	-0.155785	-2.492203

C	4.446587	-0.180071	-2.322778
C	4.734047	-0.791774	-1.071409
C	5.335857	-0.015171	-0.095774
C	4.888031	1.102169	-2.585488
C	2.675122	1.139248	-2.845147
O	4.517700	-0.064600	1.103599
O	3.849055	1.848355	-3.187120
C	1.080341	1.249677	-1.020873
C	1.591090	1.789054	-2.212529
H	0.463314	1.884363	-0.392930
H	1.334113	2.813192	-2.463501
C	5.816226	1.718526	-1.715776
C	6.019036	1.166782	-0.440535
H	6.156933	2.733040	-1.896112
H	6.493366	1.783562	0.316133

5C-S

S	-0.239491	3.761505	-0.496960
C	-1.436647	2.539119	0.097379
C	1.147380	2.761356	0.100379
C	-0.769336	1.629761	0.879492
C	0.643191	1.751483	0.881371
C	-1.101189	0.261487	0.907198
C	1.203835	0.459948	0.910475
C	0.122652	-0.468152	0.941898
C	0.231866	-1.726866	0.370554
C	-2.228337	-0.213938	0.247071
C	2.397512	0.184145	0.253401
C	1.558542	-2.111915	-0.036737
C	2.592233	-1.197569	-0.103277
C	-2.183068	-1.608771	-0.109176
C	-1.008569	-2.332985	-0.039478
C	-2.693474	2.126572	-0.428477
C	-3.083468	0.792706	-0.315498
C	2.457572	2.569627	-0.422171
C	3.069330	1.322114	-0.307514
H	1.722764	-3.112909	-0.428633
H	3.533802	-1.506761	-0.551015
H	-3.056812	-2.074532	-0.558821
H	-0.998626	-3.347574	-0.430699
H	-3.307472	2.802044	-1.016315
H	-4.001605	0.490765	-0.813823
H	2.948388	3.339933	-1.009199
H	4.026527	1.181608	-0.804024

5C-2$S_{1,2}$

S	0.765057	3.673135	-0.572682
C	-0.644092	2.777327	0.159851
C	1.928643	2.502778	0.134254
C	-0.145648	1.822007	1.017196
C	1.270625	1.719445	1.030972
C	-0.681353	0.509086	1.050357
C	1.618422	0.342808	1.019201
C	0.419046	-0.413641	1.051460
C	2.647571	0.017772	0.164047
C	0.336834	-1.635443	0.386440
C	-1.869337	0.214237	0.384194
C	-1.946610	2.571169	-0.361891
C	-2.555076	1.327446	-0.199071
C	-2.048186	-1.168593	0.013456
C	-0.993186	-2.053236	0.014646
C	1.553439	-2.117062	-0.194706
C	2.672648	-1.301348	-0.355857
S	3.284910	1.560509	-0.569349
H	-2.425547	3.308557	-0.999173
H	-3.503993	1.159525	-0.702932
H	-2.997478	-1.486455	-0.411313
H	-1.139952	-3.043664	-0.409716
H	1.554311	-3.081530	-0.697050
H	3.483652	-1.645326	-0.990837

5C-2$S_{1,3}$

S	4.811802	-0.238354	2.676561
C	3.514937	-0.717805	1.514390
C	6.099516	-0.844396	1.551825
C	4.066148	-1.508868	0.533613
C	5.492080	-1.600798	0.572841
C	3.644154	-1.419577	-0.818825
C	5.957172	-1.593113	-0.753066
C	4.818921	-1.495830	-1.612371
C	7.351141	-0.259960	1.232514
C	7.040662	-0.829356	-1.130191
C	7.816227	-0.252504	-0.092934
C	4.998396	-0.692126	-2.713824
S	6.735275	-0.206136	-2.806496
C	2.585935	-0.587537	-1.184946
C	2.668345	-0.024508	-2.504198
C	3.845823	-0.043584	-3.243012
C	2.281590	-0.071217	1.214835
C	1.823553	-0.039679	-0.097481
H	7.893201	0.334906	1.961557
H	8.693060	0.347601	-0.317360
H	1.847731	0.586020	-2.873374
H	3.905602	0.552838	-4.148388
H	1.759656	0.514666	1.965352
H	0.949153	0.569238	-0.314555

5C-3$S_{1,2,3}$

C	5.485937	-0.893613	-0.739314
C	5.107342	-1.264979	0.590117
C	6.471090	0.068718	-0.801262
S	7.010001	0.459881	0.887999
C	5.661398	-0.507211	1.576479
C	4.305233	-0.768093	-1.533955
C	3.193783	-1.056286	-0.683450
C	3.685028	-1.366017	0.624588
C	3.077786	-0.691081	1.639210
C	2.080313	-0.243682	-0.694032
C	4.210113	0.233295	-2.506344
S	4.357029	-0.038735	2.735874
S	1.573622	0.072275	1.020830
C	2.895514	0.759869	-2.742835
C	1.858952	0.569859	-1.838606
C	6.519605	0.901042	-1.952764
C	5.424111	0.939360	-2.804969
H	2.751300	1.489860	-3.535471
H	0.955515	1.162971	-1.943674
H	7.324163	1.614961	-2.099419
H	5.425666	1.679458	-3.601283

5C-3$S_{1,2,4}$

C	5.403709	-2.702441	-0.377186
C	4.175446	-3.124647	-0.978942
C	6.371874	-2.291998	-1.273256
S	5.769678	-2.648946	-2.961430
C	4.128011	-3.003189	-2.334950
C	5.070648	-1.896728	0.735563
C	3.636090	-1.780146	0.796209
C	3.085856	-2.514270	-0.279192
C	2.124510	-1.948180	-1.093914
C	3.179146	-0.511533	1.098035
C	5.749884	-0.720579	0.990398
S	2.517968	-2.386110	-2.824274
S	4.580324	0.495043	1.652358
C	7.236078	-1.244654	-0.878659
C	6.931917	-0.471484	0.252645
H	8.032092	-0.896401	-1.530006
H	7.514859	0.429251	0.419851
C	1.994740	-0.070846	0.460376
C	1.475997	-0.778007	-0.635734
H	1.580408	0.910724	0.669997
H	0.694004	-0.302600	-1.220237

5C-4S

C	4.452123	-0.998274	-0.915742
C	3.012612	-0.982108	-0.852739
C	5.025546	0.240894	-1.151045
S	3.698386	1.440290	-1.493327
C	2.463386	0.264389	-0.924802
C	4.933483	-1.811683	0.144517
C	3.799942	-2.311849	0.879936
C	2.611987	-1.762119	0.286388
C	1.804325	-1.102496	1.159544
C	3.897736	-2.158065	2.231756
C	5.975759	-1.364345	0.940381
S	1.263757	0.451665	0.406347
S	5.593109	-1.759206	2.676704
C	6.281311	0.521257	-0.568858
C	6.757060	-0.282526	0.478135
H	6.786720	1.465260	-0.749413
H	7.605269	0.083913	1.048599
S	2.372510	-1.422767	2.847359

5C-5S

C	-1.194994	-1.243058	0.647730
C	-1.549131	0.137722	0.885352
C	0.216264	-1.395527	0.919955
C	-0.356895	0.838998	1.304537
C	0.734325	-0.108469	1.325491
C	-1.586475	-1.721222	-0.567501
C	0.919445	-1.991294	-0.084685
C	1.839055	0.293631	0.635226
C	-0.098329	1.976218	0.598506
C	-2.215453	0.731017	-0.145499
S	-2.671738	-0.518803	-1.367265
S	-0.226753	-2.642299	-1.319840
S	2.444637	-1.070594	-0.381980
S	1.651450	2.023632	0.151328
S	-1.511210	2.365249	-0.457456

5C-Se

C	-1.588714	-1.712336	0.186124
C	0.949386	-1.938833	-0.746215
C	-1.238437	-0.977379	-0.916921
C	0.092157	-1.095888	-1.405613
C	-1.661862	0.353279	-1.103344
C	0.497101	0.160962	-1.896175
C	-0.591537	1.058338	-1.719272
C	-0.394123	2.413680	-1.510708
C	-2.543800	0.965083	-0.221095
C	1.820992	0.576225	-1.824598
C	0.953443	2.879890	-1.715558
C	2.012912	2.003788	-1.858502

C	-2.486941	2.404641	-0.205730
C	-1.463954	3.095123	-0.827647
C	-2.628248	-1.186787	1.001998
C	-3.107052	0.103933	0.777106
C	2.335350	-1.629330	-0.821904
C	2.750860	-0.418225	-1.375446
H	1.165242	3.943415	-1.633468
H	3.022862	2.406597	-1.878576
H	-3.194578	2.960815	0.404723
H	-1.404384	4.172227	-0.689816
H	-2.990304	-1.731098	1.869148
H	-3.832587	0.505194	1.480869
H	3.079664	-2.272228	-0.361479
H	3.810064	-0.176220	-1.327571
Se	-0.107033	-2.928404	0.566487

5C-2Se$_{1,2}$

C	-3.781530	-1.690837	0.640688
C	-4.894484	-0.441239	-1.488345
C	-3.172005	-1.827206	-0.583347
C	-3.786614	-1.196588	-1.703145
C	-1.769053	-1.709666	-0.730184
C	-2.754912	-0.662726	-2.527890
C	-1.510179	-0.986966	-1.936782
C	-2.975538	0.559306	-3.116015
C	-0.413473	-0.138291	-2.057636
C	-0.936453	-1.598389	0.379688
C	-2.958108	-1.771173	1.791510
C	-1.569771	-1.766418	1.650777
C	0.342625	-0.976941	0.130862
C	0.592046	-0.280638	-1.031495
C	-0.586514	0.979779	-2.932264
C	-1.840122	1.351335	-3.419975
H	-3.379525	-1.718001	2.790989
H	-0.968090	-1.724661	2.555587
H	1.081172	-0.944608	0.928337
H	1.519775	0.280280	-1.116290
H	0.244309	1.662043	-3.095428
H	-1.938938	2.307066	-3.926144
Se	-5.473567	-0.742308	0.334053
Se	-4.824345	1.071052	-2.694910

5C-2Se$_{1,3}$

C	3.216652	0.322001	-3.003497
C	5.921033	0.219762	-2.948242
C	3.802650	-0.402634	-1.995130
C	5.231772	-0.473161	-1.977875
C	3.332845	-0.333019	-0.659782
C	5.644896	-0.465444	-0.634670
C	4.471981	-0.389973	0.181838
C	7.175010	0.761584	-2.583742
C	6.757799	0.236022	-0.226936
C	7.588459	0.769639	-1.238976
C	4.550987	0.348079	1.336859
C	2.216932	0.431522	-0.321444
C	2.210878	0.951673	1.015965
C	3.347931	0.937068	1.817530
C	1.949399	0.909720	-2.731892
C	1.458957	0.936829	-1.430135
H	7.778889	1.310445	-3.300312
H	8.488739	1.324273	-0.991484
H	1.348296	1.513115	1.367099
H	3.330863	1.485624	2.754572
H	1.407097	1.448315	-3.503300
H	0.546226	1.497388	-1.242433
Se	6.415508	0.853615	1.597458
Se	4.610773	0.818164	-4.272025

5C-3Se$_{1,2,3}$

C	4.347147	-1.109675	-1.113526
C	5.734525	-1.190096	-0.763989
C	4.011463	-0.312610	-2.182118
C	6.604136	-0.395500	-1.440647
C	3.577353	-1.088830	0.083792
C	4.484025	-1.158127	1.178948
C	5.819890	-1.220229	0.663710
C	6.765832	-0.452564	1.264751
C	4.279400	-0.406777	2.311982
C	2.399599	-0.341358	0.169444
C	2.062334	0.139908	1.477825
C	2.985638	0.144025	2.516834
C	2.704212	0.243645	-2.208608
C	1.910959	0.193773	-1.068424
H	1.127326	0.674912	1.624677
H	2.743873	0.684527	3.427204
H	2.357395	0.821367	-3.060282
H	0.966395	0.732075	-1.080429
Se	5.676042	0.347712	-2.969064
Se	7.865807	0.314280	-0.142447
Se	6.027587	0.224142	2.922367

5C-3Se$_{1,2,4}$

C	4.748373	-1.093622	1.083125
C	3.516125	-1.295249	1.786375
C	5.835424	-0.658875	1.812526
C	3.493815	-1.004275	3.114231

C	4.434972	-0.516211	-0.165839
C	3.010290	-0.327636	-0.231139
C	2.446837	-0.789233	0.977682
C	1.448856	-0.078815	1.611615
C	2.552086	0.824075	-0.838411
C	5.228885	0.469911	-0.715779
C	6.767028	0.179074	1.164666
C	6.468664	0.732261	-0.093551
H	7.651016	0.542613	1.680263
H	7.141694	1.489272	-0.484817
C	1.371844	1.406036	-0.327196
C	0.828512	0.964129	0.892427
H	0.955777	2.306629	-0.768741
H	0.026026	1.550344	1.330357
Se	4.069148	1.661877	-1.742624
Se	5.308470	-0.700659	3.706449
Se	1.774940	-0.232328	3.544395

5C-4Se

C	3.656444	-1.368757	-2.827335
C	5.051657	-1.015770	-2.878157
C	2.741824	-0.333727	-2.879605
C	5.356720	0.309451	-2.900555
C	3.497257	-2.422494	-1.895586
C	4.790690	-2.743934	-1.350229
C	5.750976	-1.848128	-1.936688
C	6.637415	-1.245291	-1.103543
C	4.863081	-2.960768	-0.009533
C	2.424780	-2.431991	-1.023803
C	1.520783	-0.491125	-2.192169
C	1.362703	-1.537066	-1.267331
H	0.756883	0.280288	-2.217191
H	0.485127	-1.517710	-0.627950
Se	3.737735	1.313941	-3.251003
Se	6.850489	0.594727	-1.698559
Se	6.449693	-2.064536	0.651581
Se	3.064924	-3.141878	0.688533

5C-5Se

C	-1.199249	-1.241867	0.640060
C	-1.554861	0.137114	0.875427
C	0.209179	-1.393110	0.918301
C	-0.366479	0.838271	1.299421
C	0.723847	-0.107350	1.325394
C	-1.676356	-1.823778	-0.492827
C	0.947672	-2.105626	0.025798
C	1.906625	0.290371	0.784440
C	-0.125061	2.052403	0.736321
C	-2.339143	0.745754	-0.054205
Se	-2.945169	-0.603257	-1.314052
Se	-0.267050	-2.927272	-1.248352
Se	2.652402	-1.203490	-0.208411
Se	1.778498	2.184351	0.371414
Se	-1.681754	2.555683	-0.311729

5C-Te

C	-1.656348	-1.720868	0.239601
C	1.033063	-1.960900	-0.748919
C	-1.206114	-0.971641	-0.817931
C	0.132367	-1.090889	-1.309551
C	-1.627445	0.357847	-1.023221
C	0.523570	0.166239	-1.813285
C	-0.562249	1.059911	-1.640015
C	-0.380545	2.421789	-1.475675
C	-2.549610	0.981969	-0.194464
C	1.845237	0.590396	-1.809036
C	0.960273	2.891959	-1.715767
C	2.022043	2.019615	-1.871877
C	-2.499886	2.422457	-0.211009
C	-1.467295	3.108146	-0.824128
C	-2.731502	-1.176707	0.995443
C	-3.174468	0.126828	0.768788
C	2.409950	-1.635043	-0.894096
C	2.799570	-0.405610	-1.426337
H	1.166724	3.958562	-1.666082
H	3.027119	2.429966	-1.936364
H	-3.231244	2.987654	0.362081
H	-1.424048	4.189308	-0.714726
H	-3.164562	-1.730835	1.823115
H	-3.934515	0.530556	1.433712
H	3.180616	-2.296398	-0.508910
H	3.860082	-0.164466	-1.430032
Te	-0.094302	-3.153384	0.655502

5C-2Te$_{1,2}$

C	-3.779931	-1.745549	0.726461
C	-5.014308	-0.437122	-1.511406
C	-3.190158	-1.710916	-0.514197
C	-3.815522	-1.067076	-1.631814
C	-1.792348	-1.586725	-0.666976
C	-2.772055	-0.543153	-2.463320
C	-1.535124	-0.868594	-1.865842
C	-2.937975	0.606042	-3.198093
C	-0.402425	-0.080672	-2.029356
C	-0.928119	-1.548002	0.420233
C	-2.917045	-1.890459	1.843233

C	-1.531182	-1.817006	1.686648
C	0.358919	-0.949706	0.151712
C	0.608713	-0.252639	-1.012074
C	-0.530063	0.978461	-2.979116
C	-1.764010	1.329808	-3.531045
H	-3.317197	-1.962643	2.850458
H	-0.914801	-1.838457	2.582315
H	1.118892	-0.954154	0.929532
H	1.557310	0.269411	-1.113311
H	0.323462	1.618976	-3.188342
H	-1.817323	2.226767	-4.141369
Te	-5.744906	-0.889182	0.448151
Te	-5.016099	1.146794	-2.949843

5C-2Te$_{1,3}$

C	3.139986	0.370429	-3.024775
C	6.009811	0.250956	-2.982168
C	3.815011	-0.249732	-2.003071
C	5.247303	-0.316654	-1.984472
C	3.354921	-0.192508	-0.667399
C	5.660899	-0.308536	-0.639745
C	4.486153	-0.236781	0.179656
C	7.302467	0.688603	-2.623367
C	6.850143	0.267696	-0.249187
C	7.716446	0.696798	-1.277093
C	4.499393	0.396961	1.397192
C	2.186978	0.477372	-0.312488
C	2.135916	0.929619	1.046629
C	3.257778	0.907243	1.871123
C	1.844915	0.879729	-2.725233
C	1.379879	0.914792	-1.413185
H	7.966721	1.135487	-3.357396
H	8.676867	1.149425	-1.048042
H	1.239005	1.421717	1.415491
H	3.189271	1.377511	2.847726
H	1.238236	1.339854	-3.499722
H	0.429092	1.406059	-1.219964
Te	6.562508	0.871370	1.806836
Te	4.614874	0.833195	-4.527901

5C-3Te$_{1,2,3}$

C	4.363124	-0.949830	-1.110438
C	5.754750	-1.046644	-0.766573
C	3.944528	-0.291358	-2.240480
C	6.715192	-0.459073	-1.528999
C	3.606110	-0.916594	0.085585
C	4.499812	-0.997993	1.180603
C	5.840524	-1.076843	0.669897
C	6.886186	-0.519401	1.336718
C	4.219863	-0.388249	2.378773
C	2.384390	-0.250066	0.172298
C	2.005668	0.155525	1.493427
C	2.899634	0.109593	2.559265
C	2.613715	0.210821	-2.241788
C	1.852823	0.209849	-1.076374
H	1.042969	0.635160	1.652750
H	2.598167	0.552927	3.503951
H	2.203429	0.693052	-3.124383
H	0.879078	0.693124	-1.100048
Te	5.744303	0.247762	-3.298326
Te	8.262474	0.121742	-0.170391
Te	6.134197	0.111097	3.236535

5C-3Te$_{1,2,4}$

C	4.764674	-0.932190	1.151481
C	3.533470	-1.138000	1.862587
C	5.945627	-0.659642	1.810052
C	3.481888	-1.057489	3.219870
C	4.454734	-0.331545	-0.084418
C	3.031911	-0.143479	-0.149706
C	2.466885	-0.628679	1.045994
C	1.342679	-0.051515	1.598871
C	2.478894	0.919330	-0.834185
C	5.323569	0.543344	-0.703762
C	6.912090	0.088255	1.108142
C	6.605942	0.678517	-0.133596
H	7.871435	0.328957	1.557064
H	7.347680	1.338932	-0.573249
C	1.229669	1.389313	-0.379837
C	0.670499	0.913246	0.821982
H	0.727249	2.214234	-0.876500
H	-0.229985	1.399790	1.185611
Te	4.099926	1.829033	-1.935114
Te	5.496308	-0.898096	3.913552
Te	1.523638	-0.372998	3.731173

5C-4Te

C	3.631095	-1.236588	-2.677334
C	5.027864	-0.897060	-2.751811
C	2.647327	-0.286599	-2.863850
C	5.428946	0.380373	-2.989344
C	3.472681	-2.285487	-1.749961
C	4.766370	-2.629171	-1.220782
C	5.734744	-1.751968	-1.830699
C	6.825929	-1.313531	-1.148270
C	4.908549	-3.068023	0.058353

C	2.327100	-2.406142	-0.989524
C	1.403594	-0.515293	-2.241215
C	1.245285	-1.562988	-1.314722
H	0.578613	0.181010	-2.360402
H	0.306952	-1.617197	-0.770438
Te	3.680575	1.502537	-3.498405
Te	7.261862	0.632727	-1.922346
Te	6.810670	-2.356336	0.719868
Te	2.941025	-3.395034	0.831759

5C-5Te

C	-1.158472	-1.199943	0.459735
C	-1.513890	0.177933	0.694900
C	0.248753	-1.351087	0.737943
C	-0.326550	0.878570	1.118611
C	0.762903	-0.066288	1.144825
C	-1.775029	-1.937859	-0.502952
C	1.017508	-2.237617	0.049051
C	2.037822	0.311725	0.856638
C	-0.124106	2.187066	0.805013
C	-2.480876	0.796519	-0.035641
Te	-3.354406	-0.716278	-1.269814
Te	-0.322568	-3.346567	-1.195767
Te	2.982833	-1.396079	-0.017823
Te	1.993190	2.438967	0.637539
Te	-1.924011	2.859139	-0.135256

6C-2C$_{1,4}$

C	-3.240018	-1.271243	-3.999640
C	-3.273958	-0.056292	-4.745296
C	-2.039055	-1.839117	-3.571297
C	-2.107991	0.630736	-5.087110
C	-0.871171	-1.179940	-3.958544
C	-0.905260	0.035102	-4.704003
C	-4.467903	-1.544110	-3.440524
C	-5.440275	-0.452034	-3.928245
C	-4.525405	0.515487	-4.704601
C	-2.154268	1.992793	-5.442945
C	-2.021327	-2.773704	-2.517599
C	0.353889	-1.358007	-3.356893
C	0.296373	0.701325	-4.621317
C	1.255677	-0.193806	-3.812260
C	-4.613178	1.851606	-5.161631
C	-3.465126	2.558154	-5.532960
C	-4.490255	-2.559377	-2.455535
C	-3.305567	-3.163110	-2.022547
C	-0.728854	-3.063299	-1.977473
C	0.420007	-2.369470	-2.369860
C	0.297253	2.040451	-5.077924
C	-0.888371	2.657225	-5.489490
H	-5.928665	0.051921	-3.084845
H	-6.242540	-0.861250	-4.557928
H	2.108085	-0.539324	-4.413540
H	1.674028	0.345374	-2.953155
H	-5.565868	2.376101	-5.174401
H	-3.572739	3.600218	-5.823965
H	-5.420307	-2.846804	-1.970503
H	-3.363544	-3.898049	-1.223401
H	-0.642186	-3.792321	-1.175497
H	1.351977	-2.584779	-1.852235
H	1.206894	2.636311	-5.059140
H	-0.851455	3.704632	-5.778794

6C-2C$_{1,3}$

C	2.964511	0.691569	-1.773920
C	3.624376	-0.342400	-1.074029
C	3.673559	1.708238	-2.424672
C	4.989886	-0.366268	-1.029602
C	5.099659	1.683202	-2.378535
C	5.729221	0.643267	-1.684152
C	1.642009	0.772016	-1.365711
C	1.404533	-0.351718	-0.325336
C	2.820650	-0.938711	-0.113448
C	5.708512	-0.988940	-0.019633
C	2.970101	2.865963	-2.815445
C	5.866934	2.815165	-2.721811
C	7.024675	0.678161	-1.191411
C	3.505311	-1.649012	0.878185
C	4.936641	-1.673898	0.924706
C	0.928162	1.897436	-1.798349
C	1.581932	2.900568	-2.534293
C	3.769510	3.953236	-3.320684
C	5.140290	3.929204	-3.276307
C	7.154912	-0.451592	-0.138595
H	2.972038	-2.124584	1.697879
H	5.398725	-2.166904	1.776688
H	1.013980	3.782071	-2.822342
H	3.267292	4.844539	-3.690043
H	5.696157	4.801959	-3.611567
C	7.234378	2.801634	-2.351765
C	7.803948	1.777243	-1.576210
H	0.693822	-1.106410	-0.688258
H	0.991453	0.055067	0.605123
H	7.861518	-1.230719	-0.454835
H	7.519989	-0.057689	0.817142
H	7.850212	3.662403	-2.601557

H	-0.114626	2.047061	-1.528702
H	8.831832	1.890645	-1.239915

6C-2C$_{1,2}$

C	-0.563379	2.587000	0.376169
C	0.824806	2.590331	0.566754
C	-1.242595	1.376631	0.252134
C	1.584563	1.414647	0.505259
C	-0.489634	0.152374	0.296665
C	0.945349	0.182579	0.384189
C	-0.958816	3.811975	-0.127626
C	0.336080	4.671231	-0.282723
C	1.457257	3.704291	0.164202
C	2.870986	1.721569	0.103753
C	-2.567871	1.435986	-0.229958
C	-1.122602	-1.046490	-0.055294
C	1.751123	-0.921510	0.031700
C	2.911530	3.265759	-0.126381
C	-2.291191	3.882976	-0.571472
C	-3.076480	2.721964	-0.571518
C	-3.216437	0.179928	-0.460268
C	-2.523438	-1.001020	-0.359750
H	-2.716473	4.800113	-0.972255
H	-4.095995	2.788775	-0.943487
H	-4.257410	0.166347	-0.774315
H	-3.030709	-1.935156	-0.590410
C	-0.304629	-2.212245	-0.225030
C	1.067079	-2.158187	-0.200271
H	0.299877	5.609486	0.286691
H	0.458864	4.950691	-1.337810
H	-0.794114	-3.156085	-0.454590
H	1.643435	-3.054743	-0.415751
C	3.134489	-0.668221	-0.195115
C	3.686858	0.619985	-0.209528
H	3.664234	3.772319	0.492251
H	3.179748	3.467689	-1.172156
H	3.772285	-1.506052	-0.466289
H	4.721706	0.740064	-0.521473

6C-3C$_{1,3,5}$

C	4.208211	-1.337242	-1.417330
C	5.329442	-0.573699	-1.864298
C	2.924532	-0.852941	-1.599705
C	5.132489	0.650231	-2.479616
C	2.721195	0.410033	-2.234798
C	3.807816	1.149847	-2.668116
C	4.554368	-2.120297	-0.314932
C	6.447474	-0.831225	-1.069355
C	1.897674	-1.117663	-0.692058
C	6.039689	1.702182	-2.342515
C	1.554497	1.014880	-1.764209
C	3.803261	2.545717	-2.661078
C	6.086720	-2.015293	-0.143996
C	5.278316	3.006899	-2.668751
C	0.819893	-0.028175	-0.891775
C	3.514789	-2.500244	0.532993
C	2.192497	-2.001558	0.345218
C	1.483532	2.403398	-1.869276
C	2.602868	3.165269	-2.315428
C	7.424984	0.162146	-1.024505
C	7.221971	1.423110	-1.658067
H	6.598939	-2.941105	-0.440545
H	5.574575	3.424793	-3.640885
H	3.713547	-3.073138	1.435711
H	1.456933	-2.222034	1.115276
H	0.624868	2.947833	-1.483377
H	2.535421	4.248382	-2.244990
H	8.302560	0.051487	-0.391870
H	7.955713	2.204073	-1.472675
H	-0.076696	-0.422730	-1.389616
H	0.499010	0.400681	0.063473
H	6.362357	-1.811267	0.896125
H	5.462353	3.779351	-1.914430

6C-3C$_{1,2,4}$

C	4.729903	-1.578740	-1.350262
C	6.106522	-1.501676	-0.921759
C	3.659520	-1.457819	-0.431726
C	6.418063	-1.373242	0.412502
C	3.991027	-1.353010	0.932601
C	5.353597	-1.373872	1.348951
C	4.633374	-1.169217	-2.664607
C	6.903075	-0.935561	-1.912370
C	2.417777	-0.937793	-0.871532
C	7.454599	-0.566896	0.875117
C	3.252853	-0.626921	1.848663
C	5.599408	-0.622729	2.439429
C	6.051585	-0.876611	-3.192959
C	7.050722	-0.106272	2.311015
C	3.352133	-0.817999	-3.147635
C	2.271375	-0.730992	-2.277493
C	4.232538	-0.136648	2.949043
C	8.061922	-0.277824	-1.483718
C	8.328125	-0.074432	-0.099797
H	3.212808	-0.519933	-4.184322
H	1.318815	-0.372459	-2.660130

H	9.150000	0.587766	0.162757
C	1.542215	-0.429825	0.135761
C	1.956274	-0.235921	1.453631
H	6.098542	0.110231	-3.667657
H	6.378327	-1.610786	-3.942821
H	7.727885	-0.477536	3.092192
H	7.077842	0.989508	2.366431
H	4.201803	0.960359	2.993873
H	3.974391	-0.497065	3.954320
H	0.567717	-0.050954	-0.162896
H	8.710155	0.222921	-2.199060
H	1.299304	0.305349	2.130758

6C-3C$_{1,2,3}$

C	3.529673	-0.186226	-1.106710
C	2.850329	0.767609	-1.887877
C	4.978454	-0.259711	-1.183535
C	3.551477	1.585216	-2.812787
C	5.664036	0.625631	-2.036538
C	4.954906	1.514835	-2.886187
C	2.856481	-0.674355	0.034939
C	1.717356	1.454190	-1.469392
C	5.715705	-0.819563	-0.116862
C	3.087993	2.846642	-2.898214
C	6.897910	1.192826	-1.743191
C	5.530461	2.724143	-3.025105
C	4.351580	3.741505	-3.072428
C	3.616797	-1.474083	0.958048
C	4.980235	-1.543586	0.885525
C	6.938888	2.598306	-2.410485
C	1.747239	2.859948	-2.136851
H	3.099649	-1.948883	1.788684
H	5.531436	-2.072828	1.659439
C	7.080709	-0.450258	0.016541
C	7.655184	0.579125	-0.734848
H	7.655890	-0.884731	0.830664
C	1.557256	-0.170219	0.309708
C	1.012890	0.915116	-0.383028
H	4.339655	4.406414	-3.945888
H	4.429108	4.392656	-2.189713
H	7.098824	3.352829	-1.629038
H	7.757132	2.717178	-3.133468
H	0.872724	3.064937	-2.768901
H	1.749017	3.622853	-1.347230
H	1.030873	-0.548421	1.182802
H	8.644101	0.940446	-0.462607
H	0.099733	1.373258	-0.010364

6C-4C$_{1,2,3,4}$

C	-2.394335	2.480777	-0.322607
C	-2.512949	2.353888	-1.751670
C	-1.120957	2.600868	0.303563
C	-1.359123	2.381650	-2.581164
C	0.031235	2.611513	-0.533155
C	-0.083712	2.483862	-1.962329
C	-3.507990	3.162550	0.142244
C	-3.515237	3.101843	-2.259023
C	-1.049693	3.285441	1.548469
C	-1.494415	3.243085	-3.609758
C	1.129460	3.412013	-0.259972
C	0.731871	3.328958	-2.627367
C	-0.097315	3.896155	-3.811987
C	-3.476458	3.665303	1.458455
C	-2.277608	3.676443	2.160378
C	1.730549	3.886724	-1.606836
C	-2.967538	3.740835	-3.564626
H	-4.345794	4.153027	1.893660
H	-2.243920	4.156971	3.135231
C	0.216329	3.810281	1.944143
C	1.271276	3.920176	1.046848
H	0.299595	4.293251	2.914788
H	2.145787	4.501134	1.331042
C	-4.377859	3.559231	-1.077009
H	-0.192415	4.981137	-3.662687
H	0.342560	3.761758	-4.809338
H	2.766472	3.557332	-1.768762
H	-3.554743	3.544574	-4.471840
H	-2.967568	4.831769	-3.429692
H	-5.385746	3.121416	-1.062384
H	1.747243	4.984437	-1.619863
H	-4.511903	4.648999	-1.076054

6C-4C$_{1,2,3,5}$

C	5.289818	1.019383	-2.610534
C	5.938927	0.047712	-1.857588
C	3.832219	1.041781	-2.690910
C	5.167349	-0.997532	-1.250990
C	3.074698	0.091604	-2.015985
C	3.742381	-0.975976	-1.329758
C	5.776870	2.327943	-2.657034
C	7.008589	0.367700	-1.015042
C	3.393363	2.364529	-2.787958
C	5.596994	-1.308468	-0.009908
C	1.929029	0.445191	-1.295680
C	3.169123	-1.272625	-0.144440
C	4.328357	-1.567712	0.838737

C	4.617463	3.256104	-3.057616
C	1.824846	-0.524058	-0.083532
C	6.949108	-0.601812	0.199786
C	2.133110	2.672739	-2.269765
C	1.408971	1.722467	-1.506424
H	1.764667	3.695683	-2.290342
C	6.981507	2.598319	-2.003123
C	7.587654	1.627857	-1.165944
H	4.286547	-2.546868	1.335083
H	4.294121	-0.817479	1.642229
H	4.598338	4.168434	-2.452446
H	4.680049	3.563222	-4.111273
H	1.735880	0.058768	0.841415
H	0.947359	-1.184135	-0.125591
H	7.804733	-1.289277	0.252550
H	6.954537	-0.020620	1.129967
H	7.381384	3.609383	-1.981554
H	0.533874	2.068318	-0.960887
H	8.407537	1.947925	-0.526944

6C-4C$_{1,2,4,5}$

C	4.306576	-0.337712	-0.010975
C	3.058648	-0.181590	-0.710705
C	5.561059	-0.085979	-0.670084
C	3.072726	0.024407	-2.080351
C	5.575577	0.120142	-2.039648
C	4.335403	0.077968	-2.768966
C	4.266465	0.188093	1.231132
C	2.153871	0.488207	0.119941
C	6.384932	0.649631	0.188601
C	2.182784	0.911680	-2.695144
C	6.414739	1.073556	-2.626317
C	4.319113	0.946763	-3.801362
C	2.878823	1.455989	-3.971281
C	5.663942	0.737254	1.560164
C	5.721510	1.563814	-3.925619
C	7.358789	1.456770	-0.399995
C	7.373420	1.668689	-1.805973
H	8.006357	2.081896	0.210717
C	2.821417	0.629189	1.513896
C	1.140762	1.219152	-0.500956
C	1.155394	1.430824	-1.906928
H	2.397993	1.127965	-4.903554
H	2.850484	2.552965	-3.972671
H	6.180732	0.186897	2.358924
H	5.605787	1.783210	1.886789
H	5.666970	2.659716	-3.930125
H	6.255689	1.271232	-4.840479
H	2.789700	1.676338	1.840223
H	2.322209	0.040846	2.296613
H	0.427467	1.792043	0.087392
H	8.031170	2.444237	-2.191956
H	0.452792	2.154006	-2.314903

6C-5C

C	0.138816	-1.827258	-0.783003
C	-1.177770	-1.316128	-1.031638
C	1.286693	-1.039758	-1.151095
C	-1.332650	-0.023250	-1.646177
C	-0.169088	0.804205	-1.907891
C	1.113049	0.306812	-1.664788
C	0.257393	-2.297188	0.478339
C	-1.141348	-2.180029	1.135570
C	-1.967931	-1.432648	0.058472
C	2.095306	1.127292	-1.083447
C	-0.491892	2.131147	-1.574680
H	-1.055422	-1.531713	2.018212
H	-1.575698	-3.124741	1.491288
C	-2.321525	0.683509	-1.053362
C	2.238413	-1.086214	-0.191473
C	1.692570	-1.969510	0.953466
C	-2.888226	-0.189933	0.088776
C	-1.985870	2.174520	-1.168722
C	3.019120	0.232434	-0.220615
H	2.307521	-2.842618	1.212527
H	1.640956	-1.353017	1.862144
H	-2.735235	0.349411	1.034454
H	-3.964258	-0.405589	0.032027
H	-2.095211	2.690740	-0.207624
H	-2.617970	2.711088	-1.890359
H	3.123960	0.663422	0.782116
H	4.034308	0.131935	-0.629882
C	0.540572	2.997065	-1.231111
C	1.843900	2.491269	-0.983458
H	0.328725	4.022562	-0.937041
H	2.565691	3.154580	-0.512226

6C-6C

C	0.152402	-1.871105	-0.857274
C	-1.176897	-1.348754	-1.111282
C	1.319697	-1.107562	-1.255849
C	-1.338526	-0.063182	-1.764190
C	-0.171099	0.699444	-2.164294
C	1.158391	0.176972	-1.910587
C	0.266544	-2.283657	0.427236
C	-1.122951	-2.157261	1.086490

C	-1.938127	-1.415934	0.006301
C	1.936049	1.115223	-1.320543
C	-0.269386	1.981604	-1.740578
C	1.130689	2.430562	-1.272346
H	-1.039096	-1.515384	1.973705
H	-1.561955	-3.102899	1.435393
H	1.066427	2.771846	-0.230324
H	1.566424	3.265734	-1.839193
C	-2.205787	0.716410	-1.076479
C	2.202301	-1.015170	-0.233432
C	1.671512	-1.884205	0.925954
C	-2.790932	-0.131600	0.072275
C	-1.664460	2.161189	-1.106158
C	2.798993	0.407750	-0.254429
H	2.320314	-2.723183	1.215307
H	1.566352	-1.257869	1.821988
H	-2.598840	0.377629	1.026343
H	-3.877781	-0.290975	0.027057
H	-1.544270	2.518506	-0.074489
H	-2.314329	2.892845	-1.607195
H	2.625201	0.884159	0.719870
H	3.883840	0.455567	-0.426179

6C-N

C	-1.902781	1.869495	-0.164245
C	-0.525340	1.903121	-0.153899
C	-2.617016	0.695172	-0.078840
C	0.243503	0.762316	-0.091266
C	-1.871443	-0.508136	-0.001719
C	-0.443394	-0.473591	0.011140
C	-2.398956	3.155804	-0.310485
C	-0.090717	3.210938	-0.305891
C	-4.020503	0.775970	-0.136332
C	1.641743	0.910779	-0.135835
C	0.312369	-1.659629	0.066741
C	-2.569854	-1.728338	0.062500
C	-3.804071	3.264436	-0.358386
C	-4.568907	2.089277	-0.267689
C	-4.701922	-0.479373	-0.066791
C	-4.002593	-1.664434	0.030522
C	-1.790628	-2.916535	0.137219
C	-0.409430	-2.883700	0.138716
C	1.740471	-1.527423	0.036041
C	2.382338	-0.310395	-0.062298
C	1.307536	3.385872	-0.359563
C	2.127317	2.248501	-0.268078
H	-4.315105	4.216461	-0.468925
H	-5.650311	2.192969	-0.311251
H	-5.788723	-0.505615	-0.096489
H	-4.557420	-2.598635	0.078354
H	-2.299007	-3.876652	0.188442
H	0.143992	-3.818588	0.189514
H	2.339271	-2.434022	0.084674
H	3.469239	-0.284297	-0.088265
H	1.772428	4.360816	-0.474030
H	3.202651	2.403131	-0.310634
N	-1.263996	3.993761	-0.393903
H	-1.287449	4.992823	-0.504192

6C-2N$_{1,4}$

C	5.514402	-1.312510	2.113127
C	5.598251	-1.276357	0.706428
C	4.299611	-1.197060	2.792894
C	4.472682	-1.123131	-0.106291
C	3.166711	-1.158110	1.976793
C	3.250615	-1.122281	0.570094
C	6.721636	-0.870422	2.640777
N	7.579658	-0.654328	1.546914
C	6.858443	-0.811691	0.349252
C	4.585148	-0.542214	-1.384630
C	4.257273	-0.681768	4.103088
C	1.974568	-0.558540	2.365127
C	2.111335	-0.500234	0.073550
N	1.283810	-0.240699	1.181354
C	7.034176	-0.359395	-0.982494
C	5.920795	-0.254686	-1.817740
C	6.736172	-0.486244	4.004899
C	5.531008	-0.420402	4.706018
C	2.967330	-0.252557	4.557253
C	1.852794	-0.165892	3.721549
C	2.150874	-0.039109	-1.266129
C	3.357148	-0.086759	-1.966755
H	8.484999	-0.220686	1.612364
H	0.441072	0.307231	1.144939
H	7.998017	-0.013699	-1.345771
H	6.072287	0.161099	-2.811054
H	7.648802	-0.162052	4.497363
H	5.561793	-0.055933	5.730077
H	2.866410	0.120119	5.573834
H	0.939424	0.277705	4.108149
H	1.288728	0.426356	-1.735702
H	3.376429	0.337153	-2.967979

6C-2N$_{1,3}$

C	3.202434	0.396845	-3.506691
C	2.333731	0.158742	-2.435462

C	4.598635	0.363829	-3.367299
C	2.850115	-0.141757	-1.202385
C	5.137698	0.050230	-2.079646
C	4.237932	-0.205958	-1.033468
C	2.526814	1.210417	-4.419322
N	1.233620	1.428101	-3.901854
C	1.157373	0.881198	-2.597083
C	2.236715	0.252480	-0.019100
C	5.377558	1.018514	-4.343214
C	6.465344	0.385578	-1.744231
C	4.507347	0.057299	0.311563
C	0.433080	1.174609	-1.418780
C	0.967569	0.863207	-0.142218
C	3.287745	1.796833	-5.448955
C	4.678296	1.654508	-5.410272
C	6.777537	1.158433	-4.054599
C	7.295509	0.856988	-2.817232
N	3.266289	0.243527	0.954113
H	0.416847	1.209175	0.727944
H	2.838638	2.416141	-6.220372
H	5.259863	2.154741	-6.181066
H	7.428464	1.594567	-4.808938
C	6.793290	0.424003	-0.357485
C	5.847173	0.307415	0.665573
H	0.586430	2.098781	-4.278862
H	3.169914	0.597894	1.890049
H	7.814891	0.668947	-0.076666
H	6.150914	0.488720	1.692799
H	-0.494058	1.739950	-1.447975
H	8.345093	1.061247	-2.618566

6C-2N$_{1,2}$

C	3.729946	-0.755579	-2.547626
C	4.982078	-0.383035	-3.017628
C	3.586024	-1.863116	-1.708235
C	6.135520	-0.925693	-2.467577
C	4.770404	-2.565126	-1.292873
C	6.066466	-2.038393	-1.625668
C	2.953079	0.399639	-2.452373
C	5.082269	0.984008	-3.126666
C	7.059074	0.109365	-2.315798
C	2.359856	-1.982559	-1.016304
C	4.669140	-3.509628	-0.255619
C	7.215423	-2.325790	-0.855084
C	1.696988	0.278689	-1.823699
C	1.410706	-0.926010	-1.176841
C	2.262461	-3.055556	-0.078958
C	3.366932	-3.800190	0.262016
H	0.992801	1.102640	-1.751606
H	1.316930	-3.238954	0.426161
H	3.274912	-4.567561	1.027115
C	5.880626	-3.978145	0.345124
C	7.098982	-3.397860	0.081207
H	5.813666	-4.747515	1.110799
H	7.974001	-3.710563	0.646373
C	8.312298	-1.414098	-0.947791
C	8.241519	-0.184039	-1.606381
H	9.048280	0.533031	-1.484287
N	6.396916	1.310851	-2.693652
H	6.887170	2.160382	-2.927366
N	3.800881	1.494304	-2.780081
H	3.451645	2.403635	-3.040435
H	0.458722	-1.010105	-0.658591
H	9.206684	-1.628829	-0.368257

6C-3N$_{1,3,5}$

C	5.536661	-0.839075	0.712999
C	4.327002	-1.567209	0.830136
C	5.617033	0.241389	-0.161959
C	3.218652	-1.202833	0.070155
C	4.489254	0.612386	-0.934835
C	3.300352	-0.103520	-0.819989
C	6.147954	-0.799185	1.967858
C	4.189177	-1.978052	2.157701
C	6.311729	1.402033	0.185747
C	1.930987	-1.235608	0.609683
C	4.485140	2.002778	-1.065830
C	2.063317	0.544116	-0.831590
N	5.406864	-1.663926	2.812465
N	1.167909	-0.269376	-0.092711
N	5.712848	2.467744	-0.531503
C	7.017692	0.277111	2.242484
C	7.099114	1.372210	1.356064
C	3.245317	2.639696	-1.284775
C	2.040627	1.914010	-1.168163
C	2.885002	-2.210704	2.642666
C	1.761592	-1.841159	1.872497
H	7.528406	0.360383	3.197886
H	7.668165	2.240839	1.675819
H	3.177621	3.717798	-1.401651
H	1.108556	2.471440	-1.201202
H	2.712630	-2.538674	3.664082
H	0.783554	-1.903916	2.341614
H	5.463094	-1.595831	3.814740
H	0.307789	0.096381	0.279641
H	5.842264	3.427752	-0.260031

6C-3N$_{1,2,4}$

C	4.666592	-1.017917	-0.237731
C	6.045750	-0.988514	0.121538
C	3.642261	-0.822050	0.722850
C	6.424197	-0.865809	1.449943
C	4.053918	-0.761824	2.072141
C	5.414835	-0.888284	2.427159
C	4.568355	-0.476592	-1.519162
C	6.763327	-0.348571	-0.886635
C	2.449427	-0.164523	0.343053
C	7.407486	0.042640	1.839439
C	3.479146	0.149688	2.960434
C	5.726699	0.007456	3.427516
N	5.870976	-0.200563	-1.972324
N	6.984195	0.587135	3.097902
C	3.308612	-0.015580	-1.971636
C	2.276143	0.106297	-1.051172
N	4.510670	0.625071	3.801078
C	7.945093	0.340995	-0.525534
C	8.255641	0.558190	0.832428
H	3.166362	0.349970	-2.984948
H	1.342960	0.554404	-1.384518
H	8.548734	0.851819	-1.270587
C	1.685558	0.451679	1.388145
C	2.188705	0.655943	2.667169
H	6.048971	0.416137	-2.747170
H	7.634318	0.949650	3.779305
H	4.354939	1.175277	4.630161
H	1.642445	1.287868	3.361754
H	9.067429	1.242192	1.063530
H	0.734457	0.913415	1.133872

6C-3N$_{1,2,3}$

C	5.073977	-1.895678	-0.502603
C	6.156975	-1.263620	-1.161687
C	3.741268	-1.372718	-0.725453
C	5.960611	-0.256919	-2.118503
C	3.598080	-0.259615	-1.589941
C	4.673410	0.248015	-2.333917
C	5.366788	-2.587503	0.692269
C	7.335425	-0.943368	-0.471804
C	2.741182	-1.557062	0.253250
C	6.832842	0.784042	-1.898262
C	2.799695	0.836254	-1.231130
C	4.698521	1.621431	-2.255077
N	6.052267	1.994699	-1.932721
C	4.258032	-3.013682	1.496922
C	2.997902	-2.518977	1.286260
N	3.488793	2.008809	-1.615655
N	7.745962	0.338386	-0.902999
H	4.447613	-3.645248	2.361738
H	2.202552	-2.764118	1.986351
C	1.680485	-0.602078	0.318566
C	1.719643	0.616384	-0.351891
H	0.892413	-0.758949	1.051019
C	6.716603	-2.578439	1.160923
C	7.688312	-1.725793	0.646706
H	6.448378	2.873309	-2.241222
H	3.046055	2.911035	-1.697155
H	8.674924	0.701939	-0.756044
H	6.950227	-3.136672	2.064165
H	1.000493	1.391473	-0.102706
H	8.630173	-1.602635	1.173700

6C-4N$_{1,2,3,4}$

C	5.812326	-0.350412	-0.173683
C	5.931808	-1.163614	0.986273
C	4.535535	-0.015319	-0.725129
C	4.787644	-1.629125	1.669605
C	3.389991	-0.527810	-0.038213
C	3.518920	-1.340683	1.121193
C	6.780620	0.657564	-0.054048
C	6.759397	-0.534769	1.892612
C	4.406933	1.208262	-1.424072
C	4.828875	-1.204172	2.971052
C	2.304680	0.329617	0.196126
C	2.713496	-0.832755	2.118521
N	3.515927	-0.692772	3.302231
C	6.762687	1.755375	-0.949219
C	5.593530	1.979320	-1.654227
N	1.879218	0.137757	1.522136
N	6.081412	-0.503782	3.158365
H	7.568523	2.482547	-0.990907
H	5.521188	2.876678	-2.264004
C	3.102755	1.796840	-1.514807
C	2.063870	1.411127	-0.686637
H	2.975786	2.690590	-2.120952
H	1.161572	2.013504	-0.632489
N	7.374669	0.539367	1.214624
H	3.118547	-0.821227	4.225002
H	6.591779	-0.560072	4.031373
H	1.042901	0.536805	1.915777
H	8.174312	1.067960	1.521996

6C-4N$_{1,2,3,5}$

C	5.382008	-1.879416	-1.096340
C	6.066747	-1.898204	0.130738
C	3.949618	-1.736963	-1.119197
C	5.326298	-1.947524	1.337924
C	3.235361	-1.618066	0.085349
C	3.913354	-1.807891	1.315441
C	5.894025	-1.145833	-2.167309
C	7.155609	-1.040057	0.328534
C	3.626136	-0.920179	-2.203350
C	5.850897	-1.029653	2.219698
C	2.330679	-0.561956	0.251584
C	3.551323	-0.802404	2.183394
N	4.755063	-0.280758	2.763578
N	4.801463	-0.742988	-2.972012
N	2.504980	-0.060206	1.574485
N	7.041320	-0.509168	1.646686
C	2.471553	-0.105015	-2.130789
C	1.833809	0.094157	-0.897996
H	2.170151	0.517339	-2.968952
C	7.183802	-0.573905	-2.055917
C	7.808570	-0.499246	-0.802811
H	4.781181	0.132764	3.685896
H	4.890581	0.052771	-3.582486
H	1.788088	0.443235	2.074952
H	7.826805	-0.154714	2.171300
H	7.628509	-0.026565	-2.882534
H	1.080410	0.873956	-0.830543
H	8.698663	0.116589	-0.709224

6C-4N$_{1,2,4,5}$

C	4.444528	-1.245710	-1.016301
C	5.687680	-0.692818	-1.453300
C	3.232280	-0.883591	-1.680359
C	5.756509	-0.080799	-2.707447
C	3.300380	-0.272219	-2.934772
C	4.581199	-0.023871	-3.518450
C	4.296072	-0.925483	0.319605
C	6.317566	-0.099211	-0.353598
C	2.329186	-0.408202	-0.722750
C	6.453916	1.123710	-2.856080
C	2.465619	0.812857	-3.225931
C	4.537081	1.229720	-4.098292
N	5.739784	1.892250	-3.821535
N	2.964963	-0.554074	0.546152
N	3.215590	1.693362	-4.060017
C	1.293026	0.462405	-1.123982
C	1.361672	1.078329	-2.387071
H	0.533313	0.805562	-0.427424
N	5.491164	-0.358920	0.780004
C	7.264081	0.925240	-0.571182
C	7.332788	1.542365	-1.833561
H	5.971362	2.834104	-4.084127
H	2.590427	-0.204058	1.410364
H	2.900806	2.601354	-4.353414
H	5.642890	0.033715	1.692403
H	7.823083	1.370078	0.247242
H	0.652382	1.870377	-2.609977
H	7.941054	2.436827	-1.933898

6C-5N

C	5.486790	-1.461458	0.983571
C	5.535175	-1.146908	-0.387688
C	4.225082	-1.764409	1.613087
C	4.322665	-1.125901	-1.168442
C	3.063136	-1.267322	-0.508581
C	3.014392	-1.585109	0.875130
C	6.057345	-0.423747	1.693339
N	6.437090	0.560696	0.758470
C	6.135819	0.087510	-0.534862
C	2.132706	-0.853008	1.693880
C	2.226596	-0.238539	-0.983439
H	6.984330	1.384455	0.966717
C	4.291690	0.048231	-1.893671
C	4.127915	-1.022798	2.773343
N	5.297421	-0.169839	2.911696
N	5.454580	0.858039	-1.568619
N	2.970358	0.513911	-1.910114
N	2.801704	-0.590335	2.902668
H	5.801738	-0.200511	3.791328
H	6.016201	1.206679	-2.338053
H	2.676407	1.405078	-2.270455
H	2.470140	0.057698	3.595903
C	1.052199	-0.138231	1.126137
C	1.099917	0.173969	-0.234197
H	0.299361	0.342548	1.744381
H	0.382445	0.887198	-0.629929

6C-6N

C	5.383764	-1.542953	0.972683
C	5.437031	-1.222287	-0.423437
C	4.135579	-1.718242	1.664758
C	4.238938	-1.053867	-1.202887
C	2.990963	-1.238255	-0.513738
C	2.938970	-1.563789	0.881988

C	6.032831	-0.524948	1.652806
N	6.565418	0.369998	0.721787
C	6.120872	-0.023592	-0.545903
C	2.222465	-0.559356	1.511566
C	2.302469	-0.048218	-0.684157
N	1.747690	0.331241	0.542876
H	6.827616	1.316599	0.947781
H	1.461104	1.274471	0.752375
C	4.255243	0.160803	-1.870199
C	4.086421	-0.923812	2.800227
N	5.334738	-0.158942	2.846107
N	5.494992	0.843882	-1.497545
N	2.985627	0.827553	-1.583777
N	2.826773	-0.180468	2.750513
H	5.849919	-0.118453	3.718126
H	6.064780	1.250268	-2.230850
H	2.468425	1.248221	-2.347207
H	2.250574	-0.138854	3.583305

6C-4P$_{1,2,3,4}$

C	-2.414243	2.999078	-0.322494
C	-2.538936	2.928543	-1.742903
C	-1.142163	3.090423	0.283630
C	-1.386446	2.963554	-2.564753
C	0.007209	3.122296	-0.536163
C	-0.109729	3.050534	-1.957652
C	-3.591206	3.298864	0.340801
C	-3.740665	3.211257	-2.331234
C	-1.039403	3.436184	1.648210
C	-1.520675	3.325124	-3.876952
C	1.244696	3.548383	-0.086145
C	0.935360	3.450807	-2.742937
C	-3.500010	3.565002	1.729406
C	-2.264281	3.602058	2.361839
H	-4.390632	3.795811	2.307941
H	-2.225548	3.848967	3.419899
C	0.267865	3.732249	2.138511
C	1.370993	3.816551	1.299362
H	0.389681	3.982842	3.189378
H	2.320596	4.143005	1.715174
H	0.627583	3.034232	-5.264611
H	3.174184	2.976751	-1.644043
H	-3.834684	2.802217	-4.872832
H	-5.687052	2.507699	-0.865875
P	-4.886442	3.677842	-0.954015
P	-3.305252	3.814193	-4.030347
P	0.150627	3.993744	-4.333564
P	2.246474	4.050891	-1.582929

6C-4P$_{1,2,3,5}$

C	5.256584	1.354013	-2.142283
C	5.904157	0.361592	-1.413796
C	3.822892	1.375570	-2.221844
C	5.142521	-0.664642	-0.784470
C	3.069035	0.404476	-1.570984
C	3.725177	-0.643050	-0.862782
C	5.910353	2.529400	-2.501900
C	7.212363	0.498712	-0.958001
C	3.248480	2.569342	-2.649737
C	5.719838	-1.412099	0.206541
C	1.723235	0.581805	-1.262028
C	3.020178	-1.370725	0.058041
C	1.869162	2.725082	-2.436231
C	1.116314	1.751305	-1.745715
H	1.368259	3.644823	-2.726573
C	7.261619	2.643889	-2.136797
C	7.903813	1.648740	-1.369207
H	4.285693	-3.358870	1.114060
H	4.697834	3.605532	-4.425183
H	0.586894	-1.600587	-0.618964
H	8.203584	-1.716807	-0.202638
H	7.818946	3.547790	-2.367966
H	0.073881	1.969123	-1.529293
H	8.921993	1.835424	-1.038375
P	4.622413	3.787938	-3.016953
P	1.317156	-0.629129	0.117486
P	4.299531	-1.947913	1.259457
P	7.428293	-0.723233	0.453935

6C-4P$_{1,2,4,5}$

C	4.279060	0.276874	0.097766
C	3.047832	0.380776	-0.616769
C	5.518854	0.481521	-0.579198
C	3.060165	0.591632	-1.989891
C	5.531400	0.690608	-1.952567
C	4.304139	0.702130	-2.681266
C	4.253886	0.387911	1.458509
C	1.917451	0.620236	0.158676
C	6.601747	0.812020	0.229733
C	1.943209	1.057037	-2.676993
C	6.626919	1.246643	-2.606526
C	4.302305	1.216423	-3.946190
C	7.750451	1.289175	-0.424188
C	7.762670	1.503266	-1.819757
H	8.628071	1.586858	0.143555
C	0.754120	1.003711	-0.530134

C	0.766795	1.218562	-1.925589
H	2.143314	0.713851	-5.187704
H	6.462915	-0.278176	2.522709
H	6.531686	0.897754	-5.123840
H	2.075725	-0.458004	2.456696
H	-0.161622	1.227911	0.010501
H	8.648931	1.955648	-2.256879
H	-0.139885	1.597557	-2.389794
P	2.561660	1.748706	-4.308346
P	2.505467	0.790873	1.932701
P	5.948106	0.932112	1.985017
P	6.003529	1.889986	-4.254634

6C-5P

C	0.767313	-1.274859	0.872142
C	-0.626740	-1.100950	1.116345
C	1.279943	-1.104624	-0.444848
C	-1.499525	-0.709787	0.047817
C	-0.997028	-0.543128	-1.243053
C	0.395132	-0.761761	-1.508987
C	1.632808	-1.122666	1.922470
P	0.567310	-0.583698	3.340378
C	-1.008938	-0.841336	2.403633
C	0.947535	-0.191977	-2.622941
C	-1.661603	0.232531	-2.191644
P	-0.396093	0.769617	-3.468027
H	0.611783	-1.706411	4.208276
H	-0.722584	-0.118101	-4.529176
C	-2.690504	-0.108752	0.451766
C	2.607723	-0.798752	-0.582301
P	3.220777	-0.550895	1.151544
P	-2.650887	0.020190	2.322998
P	2.716451	0.130994	-2.181935
H	4.082139	-1.663226	1.342971
H	-3.521495	-1.050860	2.662736
H	3.426596	-0.769091	-3.019415
C	-3.426544	0.575154	-0.528664
C	-2.918945	0.743441	-1.832789
H	-4.353337	1.081631	-0.272211
H	-3.477987	1.372014	-2.520984

6C-6P

C	0.725818	-1.431346	0.866437
C	-0.674583	-1.276964	1.119754
C	1.236856	-1.257909	-0.459217
C	-1.563672	-0.948477	0.047183
C	-1.052549	-0.774834	-1.278476
C	0.347713	-0.929961	-1.531875
C	1.586111	-1.214993	1.910536
P	0.524863	-0.630864	3.313553
C	-1.054038	-0.924339	2.388005
C	0.873300	-0.270162	-2.611390
C	-1.766496	0.022711	-2.133565
P	-0.515618	0.751442	-3.291317
H	0.567834	-1.726461	4.215894
H	-0.809672	0.103167	-4.520043
C	-2.730247	-0.304545	0.365862
C	2.549721	-0.888388	-0.588968
P	3.155896	-0.602630	1.138939
P	-2.627205	0.034503	2.185091
P	-3.146295	0.727293	-1.116528
P	2.636589	0.085122	-2.163836
H	4.050828	-1.686286	1.342417
H	-3.603393	-0.844593	2.724241
H	-4.291818	0.074603	-1.644144
H	3.360059	-0.779389	-3.027455

6C-O

C	-1.866586	1.844400	0.160615
C	-0.487950	1.870948	0.159506
C	-2.586566	0.670713	0.166304
C	0.278498	0.727222	0.176667
C	-1.845934	-0.536956	0.234022
C	-0.415171	-0.508858	0.228255
C	-2.325534	3.099274	-0.158578
O	-1.217586	3.953446	-0.327255
C	-0.077360	3.143317	-0.155796
C	-3.973647	0.764203	-0.051904
C	1.660412	0.874758	-0.044839
C	0.332901	-1.697422	0.151109
C	-2.546215	-1.753794	0.148824
C	-3.702199	3.238973	-0.376967
C	-4.490010	2.073923	-0.291888
C	-4.662874	-0.491143	-0.085579
C	-3.975489	-1.680168	0.023269
C	-1.773660	-2.947916	0.132739
C	-0.392238	-2.920865	0.133937
C	1.758363	-1.567656	0.028248
C	2.398548	-0.352445	-0.078460
C	1.293553	3.337115	-0.368979
C	2.126092	2.203729	-0.282376
H	-4.166714	4.185242	-0.633738
H	-5.557154	2.181392	-0.468948
H	-5.741223	-0.511583	-0.223404
H	-4.531708	-2.613377	-0.026246
H	-2.286051	-3.905954	0.085775

H	0.157443	-3.858104	0.088961
H	2.351032	-2.478243	-0.019812
H	3.476980	-0.330211	-0.215358
H	1.721488	4.301175	-0.623138
H	3.188555	2.352765	-0.457367

6C-2O$_{1,4}$

C	5.553150	-1.629695	0.975190
C	5.532130	-0.544106	0.068266
C	4.393939	-2.129653	1.578043
C	4.350471	0.111759	-0.293585
C	3.203746	-1.556353	1.117682
C	3.182689	-0.470704	0.210940
C	6.767488	-1.599634	1.622669
O	7.558410	-0.576008	1.086892
C	6.734305	0.115246	0.190453
C	4.373867	1.483980	-0.615307
C	4.454823	-2.690962	2.869539
C	2.080589	-1.453301	1.906949
C	2.047477	0.261790	0.474735
O	1.296215	-0.380109	1.466748
C	6.837290	1.443617	-0.269259
C	5.672629	2.087134	-0.691144
C	6.909826	-2.287221	2.844714
C	5.768418	-2.844013	3.424399
C	3.210960	-2.763899	3.579539
C	2.044732	-2.135046	3.139959
C	1.972392	1.595509	0.025221
C	3.115363	2.166955	-0.536587
H	7.768840	1.997400	-0.213416
H	5.748558	3.134140	-0.973412
H	7.852635	-2.310032	3.381504
H	5.873592	-3.307112	4.402210
H	3.195773	-3.222995	4.564770
H	1.174159	-2.101196	3.786879
H	1.090388	2.205728	0.190244
H	3.071145	3.217581	-0.812035

6C-2O$_{1,3}$

C	4.617414	-0.712744	-1.873494
C	5.531448	-0.022151	-2.683668
C	3.231804	-0.482256	-1.920760
C	5.066624	0.907590	-3.578546
C	2.746073	0.489301	-2.855169
C	3.686929	1.148835	-3.664686
C	5.296252	-1.034585	-0.710300
O	6.619387	-0.572223	-0.780960
C	6.697514	0.177364	-1.973326
C	5.742258	2.087866	-3.812063
C	2.474544	-0.837356	-0.785094
C	1.497551	1.117288	-2.664495
C	3.547716	2.463737	-4.075772
C	7.477294	1.304532	-2.277406
C	6.995313	2.268470	-3.205266
C	4.568462	-1.465063	0.403659
C	3.171792	-1.406296	0.318153
C	1.112412	-0.375001	-0.754472
C	0.647407	0.555326	-1.648798
O	4.810893	3.046211	-4.262646
H	7.553623	3.191810	-3.319477
H	5.046238	-1.746429	1.336164
H	2.599241	-1.688477	1.198083
H	0.465569	-0.700711	0.056467
C	1.286075	2.368113	-3.310726
C	2.297311	3.080500	-3.967386
H	0.326716	2.861193	-3.175596
H	2.124379	4.102594	-4.287437
H	8.382301	1.534797	-1.724835
H	-0.359140	0.948865	-1.529451

6C-2O$_{1,2}$

C	3.675811	-2.421822	-2.420011
C	4.911597	-2.691993	-3.002378
C	3.556987	-2.279862	-1.033808
C	6.085171	-2.509305	-2.274942
C	4.758188	-2.341494	-0.235465
C	6.047409	-2.370020	-0.883817
C	2.977666	-1.625455	-3.315760
O	3.811570	-1.338406	-4.437430
C	5.006537	-1.973492	-4.146518
C	6.941135	-1.769505	-3.077388
C	2.386632	-1.635532	-0.569145
C	4.693104	-1.914614	1.101823
C	7.197301	-1.809779	-0.279738
O	6.270505	-1.427978	-4.289704
C	1.791790	-1.009961	-2.905102
C	1.488048	-1.101738	-1.542140
C	2.312600	-1.388368	0.837006
C	3.414766	-1.554840	1.637665
H	1.175957	-0.410894	-3.567101
H	1.396814	-0.983514	1.260713
H	3.353143	-1.288123	2.689968
C	5.920754	-1.645370	1.788277
C	7.119910	-1.562044	1.126117
H	5.875281	-1.379230	2.841553
H	8.005118	-1.222131	1.658121

C	8.241967	-1.346985	-1.136196
C	8.110804	-1.239665	-2.525278
H	8.843146	-0.689733	-3.106390
H	0.587903	-0.605644	-1.188968
H	9.127188	-0.915802	-0.676261

6C-3O$_{1,3,5}$

C	3.522650	-0.694235	-0.660880
C	4.842430	-1.098639	-0.322006
C	2.532782	-0.651259	0.320300
C	5.147487	-1.451522	0.992105
C	2.843441	-1.012332	1.659359
C	4.138576	-1.408337	1.992067
C	3.636517	0.334726	-1.583149
C	5.712061	-0.301319	-1.050308
C	1.655744	0.420876	0.380482
C	6.322302	-1.007681	1.579500
C	2.143779	-0.147388	2.486818
C	4.735283	-0.939973	3.152539
O	4.982213	0.478591	-1.964541
O	6.125409	-0.846137	2.962330
O	1.270712	0.639540	1.715097
C	2.641416	1.321320	-1.650861
C	1.639164	1.364945	-0.657323
C	2.660440	0.176136	3.750438
C	3.971665	-0.224918	4.087341
C	6.984020	-0.009310	-0.535510
C	7.292938	-0.366760	0.794982
H	2.721799	2.151546	-2.344762
H	0.981622	2.227176	-0.619430
H	2.145361	0.871279	4.405193
H	4.421745	0.174828	4.990026
H	7.671707	0.634714	-1.073637
H	8.208137	0.013831	1.236329

6C-3O$_{1,2,4}$

C	4.611805	1.078369	-0.537218
C	5.975529	0.634832	-0.578853
C	3.551844	0.216892	-0.131763
C	6.286744	-0.696201	-0.338432
C	3.898072	-1.142625	0.047431
C	5.218208	-1.603933	-0.188519
C	4.657729	2.384310	-0.086640
C	6.755221	1.638577	-0.032548
C	2.498925	0.750102	0.650939
C	7.248644	-1.017311	0.609660
C	3.434292	-1.836265	1.154405
C	5.543799	-2.415023	0.848961
O	5.977438	2.797399	0.110786
O	6.818103	-2.182301	1.325567
C	3.520087	2.991918	0.479403
C	2.448250	2.173387	0.801109
O	4.461862	-2.680481	1.658081
C	7.903003	1.310428	0.710851
C	8.140518	-0.036086	1.063600
H	3.525118	4.034027	0.781475
H	1.610329	2.611985	1.337032
H	8.508960	2.081917	1.174365
C	1.795488	-0.149480	1.515862
C	2.280728	-1.413966	1.833572
H	1.849305	-1.978747	2.653014
H	8.906574	-0.255773	1.799646
H	0.951227	0.230449	2.085114

6C-3O$_{1,2,3}$

C	5.061295	-1.972907	-0.583421
C	6.144600	-1.318159	-1.226977
C	3.721735	-1.445412	-0.806341
C	5.954161	-0.319565	-2.202746
C	3.590638	-0.312667	-1.652534
C	4.662366	0.188946	-2.417914
C	5.355394	-2.615837	0.640353
C	7.248480	-0.916102	-0.476518
C	2.746224	-1.587795	0.206431
C	6.744788	0.729210	-1.851266
C	2.884250	0.802302	-1.203996
C	4.715882	1.527817	-2.187613
O	6.011911	1.927797	-1.823013
C	4.247289	-3.058840	1.440882
C	2.991533	-2.563840	1.232150
O	3.582364	1.988646	-1.548694
O	7.653758	0.385151	-0.870442
H	4.441544	-3.685070	2.308036
H	2.196349	-2.800203	1.934825
C	1.750563	-0.576399	0.347468
C	1.847994	0.665091	-0.278038
H	0.995862	-0.702510	1.119216
C	6.677203	-2.517409	1.167251
C	7.612699	-1.605726	0.681893
H	6.897069	-3.027830	2.101116
H	1.226851	1.496677	0.036055
H	8.507396	-1.371765	1.248236

6C-4O$_{1,2,3,4}$

C	5.842397	0.512448	-0.488743
C	5.812854	-0.912410	-0.648335

C	4.641158	1.301307	-0.462877
C	4.589546	-1.620915	-0.706460
C	3.412943	0.560401	-0.550634
C	3.393675	-0.864549	-0.710615
C	6.791207	0.759310	0.499236
C	6.480257	-1.403161	0.432044
C	4.644118	2.478774	0.330599
C	4.545177	-2.438144	0.382848
C	2.425166	0.845473	0.387876
C	2.652145	-1.327794	0.332951
O	3.310314	-2.316883	1.057590
C	6.930096	2.017027	1.103582
C	5.873969	2.895507	0.935404
O	1.901177	-0.355633	0.937025
O	5.747343	-2.364725	1.120806
H	7.743265	2.246031	1.783621
H	5.897319	3.840359	1.471425
C	3.402284	2.944382	0.871960
C	2.304987	2.108485	0.985011
H	3.388600	3.890173	1.406666
H	1.467534	2.370543	1.622269
O	7.237869	-0.461107	1.074149

6C-4O$_{1,2,3,5}$

C	5.525871	0.124469	-1.587871
C	6.201816	-1.085921	-1.336456
C	4.074362	0.146998	-1.604832
C	5.471618	-2.308143	-1.311790
C	3.355187	-1.041618	-1.369695
C	4.045858	-2.286095	-1.328449
C	5.919048	1.266966	-0.904408
C	7.095853	-1.126334	-0.267357
C	3.700851	1.301480	-0.930532
C	5.832815	-2.923535	-0.154053
C	2.435631	-1.053674	-0.321781
C	3.638549	-2.889854	-0.179877
O	4.720332	-3.341588	0.585872
O	4.820069	2.107791	-0.689133
O	2.555906	-2.276536	0.403748
O	6.920369	-2.344619	0.454720
C	2.543431	1.339955	-0.131985
C	1.913141	0.132973	0.206717
H	2.266287	2.244407	0.399175
C	7.057757	1.269575	-0.078576
C	7.641997	0.043653	0.274271
H	7.350029	2.165015	0.459709
H	1.171030	0.126186	0.997746
H	8.364394	0.013949	1.082836

6C-4O$_{1,2,4,5}$

C	4.325023	-1.012870	-2.895999
C	5.616448	-0.415657	-2.696291
C	3.153792	-0.181669	-2.936763
C	5.768369	0.957497	-2.918058
C	3.305813	1.191454	-3.158488
C	4.628103	1.723925	-3.337940
C	4.142587	-1.836875	-1.830571
C	6.145983	-0.969239	-1.534140
C	2.317337	-0.605342	-1.908029
C	6.443737	1.718495	-1.968254
C	2.615302	2.082368	-2.341985
C	4.662740	2.857275	-2.588457
O	5.808821	2.991456	-1.844551
O	2.890401	-1.748962	-1.273969
O	3.440704	3.216498	-2.075715
C	1.424993	0.272835	-1.281066
C	1.578235	1.654663	-1.504087
H	0.766941	-0.060106	-0.485676
O	5.258737	-1.973934	-1.042645
C	7.049531	-0.261782	-0.731795
C	7.202444	1.120085	-0.954907
H	7.468769	-0.697201	0.168852
H	1.034502	2.350841	-0.874567
H	7.735489	1.714045	-0.220297

6C-5O

C	5.368888	-1.538830	1.088881
C	5.447614	-1.149635	-0.298234
C	4.080722	-1.600393	1.717538
C	4.245660	-1.018905	-1.043098
C	2.960595	-1.290313	-0.419350
C	2.873397	-1.489992	0.991997
C	6.133203	-0.630469	1.780933
O	6.861112	0.187351	0.931490
C	6.347864	-0.077346	-0.347458
C	2.090700	-0.486180	1.543597
C	2.271966	-0.127944	-0.629078
O	1.679213	0.394233	0.478647
C	4.084467	0.146036	-1.797608
C	4.076117	-0.472858	2.497542
O	5.300729	0.071091	2.734342
O	2.748355	0.652164	-1.626129
O	2.907604	0.200866	2.542027
C	5.138090	1.015259	-2.092782
C	6.324609	0.888224	-1.360082
H	4.978665	1.906283	-2.690726

H	7.066928	1.677158	-1.407451

6C-6O

C	5.408686	-1.484699	1.053069
C	5.482639	-1.180381	-0.361902
C	4.119930	-1.652902	1.693569
C	4.267227	-1.043194	-1.138900
C	2.978209	-1.209345	-0.498348
C	2.904843	-1.513669	0.916341
C	5.982882	-0.414362	1.680210
O	6.513063	0.521736	0.799947
C	6.094278	0.040160	-0.435324
C	2.241842	-0.457583	1.476206
C	2.349224	-0.003717	-0.639618
O	1.788454	0.466809	0.542659
C	4.277088	0.244141	-1.599426
C	4.057019	-0.669806	2.642056
O	5.228992	0.073854	2.740399
O	5.437303	0.937053	-1.269565
O	3.071972	0.909181	-1.398815
O	2.864151	0.046325	2.611740

6C-2S$_{1,4}$

C	5.617073	-1.098772	1.601795
C	5.595605	-0.027477	0.693227
C	4.443578	-1.631435	2.136477
C	4.401627	0.583449	0.308681
C	3.249290	-1.025646	1.744780
C	3.227251	0.045630	0.836198
C	6.875451	-1.555492	1.945851
S	8.095592	-0.575799	1.074214
C	6.836611	0.425811	0.286820
C	4.384049	1.693564	-0.550501
C	4.468939	-2.665763	3.085478
C	2.015692	-1.405466	2.239208
C	1.976637	0.575838	0.580132
S	0.762164	-0.349514	1.516632
C	6.862084	1.540001	-0.587573
C	5.669376	2.143848	-0.989078
C	6.943131	-2.616488	2.881966
C	5.772502	-3.147190	3.426342
C	3.188690	-3.067394	3.582316
C	1.995942	-2.463753	3.180597
C	1.914810	1.692771	-0.288915
C	3.085450	2.223671	-0.833066
H	7.802123	1.944745	-0.949900
H	5.731800	2.999689	-1.656345
H	7.898922	-3.024864	3.195747
H	5.867446	-3.953471	4.149178
H	3.131918	-3.868996	4.314304
H	1.061523	-2.813792	3.608497
H	0.964705	2.155864	-0.537144
H	2.996018	3.084235	-1.491125

6C-2S$_{1,2}$

C	5.214301	0.743362	-3.677320
C	4.035553	0.606157	-4.392137
C	5.174719	0.796475	-2.282125
C	2.790547	0.605201	-3.784489
C	3.905587	0.744214	-1.630924
C	2.700950	0.655396	-2.391492
C	6.317954	1.019894	-4.465482
S	5.778753	1.118466	-6.192032
C	4.084124	0.799229	-5.738738
C	1.736309	0.758643	-4.668125
C	6.377746	1.059954	-1.599767
C	3.834732	0.907004	-0.238142
C	1.420474	0.777185	-1.818923
S	2.410841	0.926421	-6.341048
C	7.537253	1.252023	-3.795477
C	7.546259	1.249567	-2.399654
C	6.282073	1.169815	-0.177993
C	5.067078	1.089471	0.465683
H	8.457444	1.459490	-4.332694
H	7.183132	1.347484	0.404014
H	5.033248	1.202141	1.546816
C	2.532535	0.944905	0.353631
C	1.377630	0.890057	-0.394809
H	2.458138	1.055272	1.432969
H	0.414566	0.961416	0.104806
C	0.312933	0.836921	-2.719482
C	0.444367	0.847442	-4.109111
H	-0.442623	0.951789	-4.726169
H	8.489699	1.447687	-1.897296
H	-0.687421	0.924118	-2.303086

6C-3S$_{1,3,5}$

C	4.207432	-1.191585	-1.141442
C	5.313448	-0.393054	-1.492922
C	2.918455	-0.755942	-1.422327
C	5.115499	0.830540	-2.120442
C	2.717417	0.485187	-2.058447
C	3.808402	1.272854	-2.404061
C	4.457673	-2.154785	-0.172341
C	6.492162	-0.685777	-0.818793
C	1.813471	-1.260897	-0.748939

C	6.085784	1.824525	-2.104743
C	1.443904	1.021873	-1.918376
C	3.681975	2.638039	-2.627450
C	3.345326	-2.756561	0.441808
C	2.035227	-2.313614	0.156317
C	1.275853	2.378526	-2.247101
C	2.384610	3.179163	-2.598488
C	7.526856	0.263574	-0.885417
C	7.325604	1.507490	-1.522834
H	3.470141	-3.515280	1.208073
H	1.216169	-2.753301	0.716834
H	0.307523	2.861177	-2.158886
H	2.215087	4.238629	-2.763323
H	8.473028	0.098229	-0.379683
H	8.126596	2.238635	-1.476450
S	6.215053	-2.130168	0.222171
S	5.322632	3.379737	-2.600565
S	0.412773	-0.167013	-1.042570

6C-3S$_{1,2,4}$

C	4.739301	-1.282805	-1.362999
C	6.086035	-1.164703	-0.936932
C	3.663828	-1.187343	-0.454078
C	6.380836	-0.995812	0.406395
C	3.981590	-1.032260	0.907246
C	5.322453	-0.986091	1.319966
C	4.568278	-1.071093	-2.721281
C	7.001090	-0.801738	-1.915231
C	2.361533	-0.900210	-0.906740
C	7.544576	-0.383052	0.845225
C	3.098117	-0.493509	1.827115
C	5.573170	-0.368636	2.511393
C	3.252014	-0.908873	-3.212344
C	2.185379	-0.841958	-2.322818
C	8.258172	-0.322152	-1.490412
C	8.526918	-0.101532	-0.124563
H	3.065997	-0.767816	-4.272680
H	1.192401	-0.655635	-2.723939
H	9.470893	0.363061	0.142265
C	1.406990	-0.532312	0.095042
C	1.754691	-0.306648	1.422490
H	0.382075	-0.331835	-0.206289
H	9.013431	-0.024916	-2.211078
H	1.004346	0.070692	2.110299
S	4.026616	0.086819	3.260939
S	7.277852	0.165735	2.553495
S	6.157916	-0.776576	-3.496387

6C-3S$_{1,2,3}$

C	3.553628	-0.032822	-0.934869
C	2.874029	0.929254	-1.709695
C	4.985159	-0.101465	-1.007446
C	3.578880	1.761220	-2.587227
C	5.671894	0.794866	-1.851699
C	4.964834	1.694399	-2.657930
C	2.851707	-0.654312	0.115305
C	1.621711	1.432297	-1.379981
C	5.726860	-0.792369	-0.030551
C	3.027754	2.974400	-2.878584
C	6.993491	1.174294	-1.652768
C	5.597273	2.850322	-3.010450
C	3.612546	-1.495228	0.993775
C	4.981716	-1.561058	0.924220
H	3.092726	-2.039034	1.778807
H	5.523797	-2.155837	1.655401
C	7.130273	-0.548484	0.024776
C	7.762111	0.433301	-0.733892
H	7.720822	-1.083952	0.763853
C	1.490749	-0.277840	0.310682
C	0.882334	0.763546	-0.384949
H	0.929273	-0.758110	1.108043
H	8.810872	0.652116	-0.559044
H	-0.117769	1.080617	-0.106423
S	7.300011	2.749367	-2.484719
S	4.351222	4.108279	-3.311742
S	1.384899	3.034035	-2.183893

6C-4S$_{1,2,3,4}$

C	-2.408560	2.720525	-0.326545
C	-2.517450	2.628117	-1.733951
C	-1.136982	2.805852	0.299030
C	-1.377242	2.642435	-2.547271
C	0.011044	2.810036	-0.536042
C	-0.116646	2.717487	-1.941675
C	-3.543170	3.260215	0.263708
C	-3.620420	3.214961	-2.289585
C	-1.042452	3.325168	1.607504
C	-1.504391	3.297645	-3.737037
C	1.188010	3.434386	-0.146120
C	0.829408	3.378298	-2.674676
C	-3.491917	3.629889	1.627205
C	-2.268307	3.626304	2.279139
H	-4.372048	3.998748	2.144797
H	-2.229487	3.984292	3.304664
C	0.255030	3.720085	2.060252
C	1.345074	3.808555	1.208030

H	0.366071	4.081676	3.079215
H	2.271575	4.244545	1.568680
S	-4.751371	3.689511	-1.001787
S	-3.217539	3.813585	-3.931211
S	0.108306	3.935223	-4.219671
S	2.127661	3.940662	-1.596954

6C-4S$_{1,2,3,5}$

C	5.261568	1.235306	-2.343333
C	5.913479	0.217335	-1.639270
C	3.835963	1.256909	-2.422347
C	5.148562	-0.824304	-1.075655
C	3.079631	0.260279	-1.796834
C	3.745731	-0.803044	-1.153779
C	5.860860	2.481738	-2.487304
C	7.122328	0.446037	-0.990355
C	3.293805	2.520311	-2.629724
C	5.657946	-1.380617	0.063470
C	1.813956	0.526252	-1.285003
C	3.096809	-1.342272	-0.079157
C	1.940627	2.735092	-2.299093
C	1.205644	1.750477	-1.619491
H	1.478319	3.705235	-2.451818
C	7.174908	2.656047	-2.008529
C	7.800068	1.650891	-1.253429
H	7.680495	3.611519	-2.107091
H	0.209888	2.001133	-1.267632
H	8.757911	1.872400	-0.793313
S	1.450886	-0.656685	0.036647
S	4.615216	3.698196	-2.916950
S	7.301075	-0.744620	0.361451
S	4.307724	-1.970117	1.082041

6C-4S$_{1,2,4,5}$

C	4.295615	0.043823	0.031111
C	3.063642	0.170520	-0.654563
C	5.531544	0.282566	-0.616691
C	3.074634	0.390010	-2.032051
C	5.543052	0.501829	-1.994141
C	4.317835	0.480328	-2.703368
C	4.259218	0.399071	1.350219
C	2.011444	0.622347	0.132280
C	6.513567	0.827207	0.201471
C	2.033925	1.067277	-2.655260
C	6.536795	1.271494	-2.586350
C	4.301432	1.229698	-3.846018
C	7.625950	1.441491	-0.409361
C	7.637268	1.662887	-1.796457
H	8.432790	1.861103	0.183144
C	0.867347	1.134628	-0.513146
C	0.878737	1.356354	-1.900318
H	0.007902	1.478291	0.053747
H	8.452740	2.243460	-2.216085
H	0.027397	1.861345	-2.345594
S	2.640110	1.754892	-4.212383
S	2.590823	0.788108	1.835510
S	5.869033	0.939370	1.885534
S	5.918365	1.904737	-4.161377

6C-5S

C	0.615057	-1.793772	0.828182
C	-0.770608	-1.662512	1.080392
C	1.146656	-1.535581	-0.467942
C	-1.645642	-1.345412	0.021048
C	-1.128796	-1.161825	-1.282783
C	0.273952	-1.219597	-1.524546
C	1.413162	-1.387283	1.862795
S	0.408624	-0.836110	3.236896
C	-1.085988	-1.080497	2.275990
C	0.695422	-0.377019	-2.550338
C	-1.796424	-0.223949	-2.022549
S	-0.730600	0.459074	-3.274959
C	-2.670420	-0.506316	0.357956
C	2.434819	-1.006573	-0.444048
S	3.015944	-0.897665	1.261136
S	-2.627644	-0.172531	2.121919
S	-3.154074	0.455851	-1.076587
C	2.059022	-0.037156	-2.641550
C	2.928171	-0.351778	-1.589065
H	2.428880	0.591234	-3.445602
H	3.938087	0.044916	-1.618045

6C-6S

C	0.659146	-1.851147	0.785498
C	-0.732753	-1.697946	1.036390
C	1.167789	-1.681201	-0.532016
C	-1.615996	-1.373798	-0.030566
C	-1.107228	-1.203048	-1.347982
C	0.284504	-1.357317	-1.599054
C	1.463643	-1.335656	1.765925
S	0.477895	-0.753130	3.143122
C	-1.021759	-1.061946	2.213796
C	0.794772	-0.454232	-2.492288
C	-1.690174	-0.178257	-2.043760
S	-0.514782	0.556908	-3.177871
C	-2.598891	-0.483060	0.308513

C	2.372101	-1.032457	-0.586818
S	2.996721	-0.731107	1.064483
S	-2.537883	-0.120685	2.061476
S	-3.033519	0.535844	-1.099010
S	2.500613	-0.077466	-2.096637

6C-4Se$_{1,2,3,4}$

C	-2.417667	2.959294	-0.339233
C	-2.530237	2.859585	-1.745954
C	-1.147453	3.053031	0.276021
C	-1.386086	2.863052	-2.559259
C	0.001187	3.052035	-0.549442
C	-0.122105	2.951644	-1.955066
C	-3.581138	3.295955	0.331179
C	-3.717081	3.180645	-2.337591
C	-1.044296	3.433204	1.629266
C	-1.512966	3.219714	-3.867528
C	1.234299	3.478834	-0.086941
C	0.918035	3.356465	-2.739767
C	-3.508280	3.580275	1.713226
C	-2.269422	3.626137	2.338169
H	-4.400273	3.816133	2.285553
H	-2.230894	3.893125	3.391130
C	0.266559	3.722255	2.118053
C	1.379238	3.765350	1.289093
H	0.389712	3.992019	3.163800
H	2.336238	4.070796	1.701083
Se	-5.009922	3.524209	-0.964240
Se	-3.392724	3.536028	-4.205101
Se	0.251338	3.674498	-4.521457
Se	2.398140	3.803917	-1.605295

6C-4Se$_{1,2,3,5}$

C	5.249472	1.382954	-2.109517
C	5.902277	0.349531	-1.433835
C	3.826471	1.404606	-2.188196
C	5.139722	-0.693121	-0.866232
C	3.071990	0.392523	-1.590792
C	3.735215	-0.671727	-0.943972
C	5.917811	2.564576	-2.403922
C	7.212539	0.477297	-0.991008
C	3.230533	2.605279	-2.552720
C	5.712538	-1.466683	0.099626
C	1.725191	0.560200	-1.295463
C	3.036287	-1.425875	-0.048360
C	1.843620	2.744240	-2.370299
C	1.094214	1.732943	-1.741050
H	1.334307	3.663237	-2.643161
C	7.279460	2.662267	-2.068507
C	7.923724	1.629977	-1.361719
H	7.843097	3.565234	-2.281440
H	0.041105	1.915613	-1.551162
H	8.954264	1.781469	-1.055893
Se	4.621416	3.881236	-2.979171
Se	1.198353	-0.848905	-0.057133
Se	4.303230	-2.358028	1.062119
Se	7.556562	-0.945347	0.295307

6C-4Se$_{1,2,4,5}$

C	4.280316	0.345855	0.084540
C	3.051393	0.443093	-0.610833
C	5.516025	0.554365	-0.573389
C	3.062472	0.659755	-1.987660
C	5.527201	0.771306	-1.950184
C	4.302483	0.778399	-2.659583
C	4.253286	0.487268	1.440642
C	1.909863	0.671299	0.144362
C	6.608919	0.883082	0.215842
C	1.932548	1.112403	-2.653955
C	6.631347	1.325251	-2.582400
C	4.296511	1.331388	-3.906137
C	7.776825	1.336293	-0.425050
C	7.787836	1.556043	-1.814737
H	8.665861	1.595388	0.141349
C	0.725868	1.018174	-0.532381
C	0.737224	1.237388	-1.922198
H	-0.199477	1.196077	0.006485
H	8.684766	1.975408	-2.259690
H	-0.179925	1.574819	-2.394771
Se	2.486864	1.710927	-4.419852
Se	2.434800	0.696104	2.017260
Se	6.027316	0.856636	2.071988
Se	6.078753	1.874528	-4.364617

6C-5Se

C	0.668071	-1.582636	0.799749
C	-0.710714	-1.439722	1.082246
C	1.164822	-1.360424	-0.514598
C	-1.607989	-1.121738	0.040287
C	-1.119694	-0.949131	-1.276095
C	0.273099	-1.044330	-1.550157
C	1.532371	-1.405436	1.841399
Se	0.523741	-1.030538	3.437064
C	-1.069837	-1.092841	2.351556
C	0.720643	-0.399240	-2.697368
C	-1.881227	-0.196209	-2.122544

Se	-0.809520	0.381944	-3.606411
C	-2.768922	-0.490363	0.378274
C	2.512366	-1.034543	-0.616585
Se	3.296345	-1.073531	1.161600
Se	-2.836571	-0.314424	2.300432
Se	-3.464818	0.382734	-1.194758
C	2.101996	-0.202894	-2.868595
C	2.994228	-0.519237	-1.832315
H	2.495262	0.281559	-3.756901
H	4.042349	-0.267101	-1.960081

6C-6Se

C	0.683521	-1.668855	0.818575
C	-0.706871	-1.514578	1.070552
C	1.190431	-1.499079	-0.498356
C	-1.590335	-1.190340	0.005360
C	-1.083317	-1.020550	-1.311604
C	0.307018	-1.174985	-1.563514
C	1.534015	-1.387513	1.849848
Se	0.507462	-0.959600	3.422483
C	-1.066575	-1.098634	2.320994
C	0.829770	-0.463813	-2.606148
C	-1.770893	-0.174889	-2.135107
Se	-0.588324	0.476847	-3.508755
C	-2.718872	-0.491745	0.328455
C	2.482250	-1.070124	-0.613562
Se	3.267199	-0.937876	1.140547
Se	-2.799528	-0.262060	2.239155
Se	-3.347700	0.455513	-1.226346
Se	2.719248	-0.217819	-2.324477

6. Reference

1. Grimme, S.; Hansen, A.; Ehlert, S.; Mewes, J. r^{2}SCAN-3c: A "Swiss army knife" composite electronic-structure method. *J. Chem. Phys.* **2021**, *154*, 064103.
2. Furness, J. W.; Kaplan, A. D.; Ning, J.; Perdew, J. P.; Sun, J. Accurate and Numerically Efficient r^{2}SCAN Meta-Generalized Gradient Approximation. *J. Phys. Chem. Lett.* **2020**, *11*, 8208-8215.
3. Caldeweyher, E.; Bannwarth, C.; Grimme, S. Extension of the D3 dispersion coefficient model. *J. Chem. Phys.* **2017**, *147*, 034112.
4. Caldeweyher, E.; Ehlert, S.; Hansen, A.; Neugebauer, H.; Spicher, S.; Bannwarth, C.; Grimme, S. A generally applicable atomic-charge dependent London dispersion correction. *J. Chem. Phys.* **2019**, *150*, 154122.
5. Caldeweyher, E.; Mewes, J.; Ehlert, S.; Grimme, S. Extension and evaluation of the D4 London-dispersion model for periodic systems. *Phys. Chem. Chem. Phys.* **2020**, *22*, 8499-8512.
6. Kruse, H.; Grimme, S. A geometrical correction for the inter- and intra-molecular basis set superposition error in Hartree-Fock and density functional theory calculations for large systems. *J. Chem. Phys.* **2012**, *136*, 154101.
7. Neese, F. The ORCA program system. *Wiley Interdiscip. Rev. Comput. Mo*l. *Sci.* **2012**, *2*, 73–78.
8. Neese, F. Software update: the ORCA program system, version 5. *Wiley Interdiscip. Rev. Comput. Mo*l. *Sci.* **2022**, *12*, e1606.
9. Helmich-Paris, B.; de Souza, B.; Neese, F.; Izsák, R. An improved chain of spheres for exchange algorithm. *J. Chem. Phys.* **2021**, *155*, 104109.
10. Rickhaus, M.; Mayor, M.; Juríček, M. Chirality in curved polyaromatic systems. *Chem. Soc. Rev.* **2017**, *46*, 1643-1660.
